# CONFPASS: Fast DFT Re-Optimizations of Structures from Conformation Searches

**DOI:** 10.1021/acs.jcim.3c00649

**Published:** 2023-07-10

**Authors:** Ching Ching Lam, Jonathan M. Goodman

**Affiliations:** Yusuf Hamied Department of Chemistry, University of Cambridge, Lensfield Road, Cambridge CB2 1EW, U.K.

## Abstract

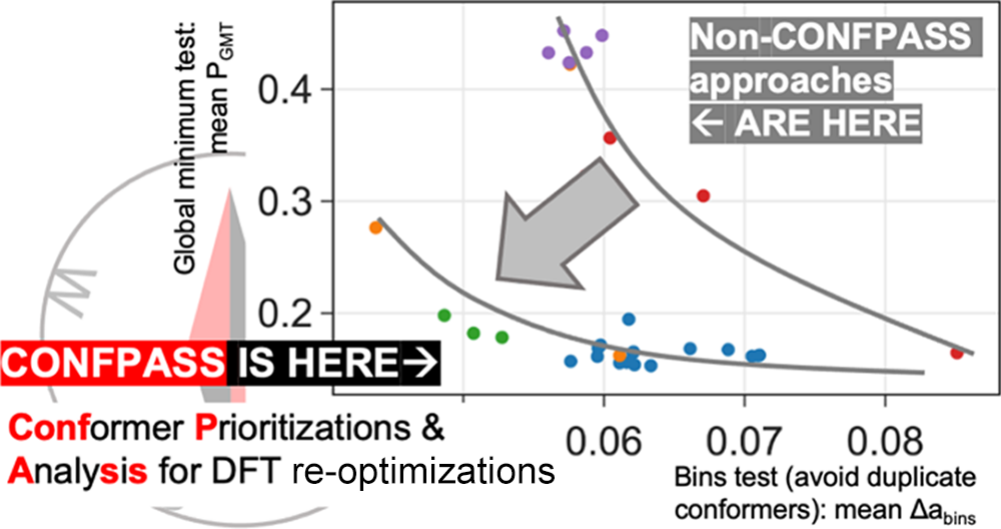

CONFPASS (**Conf**ormer **P**rioritizations and **A**naly**s**i**s** for DFT re-optimizations) has been developed to extract dihedral angle descriptors from conformational searching outputs, perform clustering, and return a priority list for density functional theory (DFT) re-optimizations. Evaluations were conducted with DFT data of the conformers for 150 structurally diverse molecules, most of which are flexible. CONFPASS gives a confidence estimate that the global minimum structure has been found, and based on our dataset, we can have 90% confidence after optimizing half of the FF structures. Re-optimizing conformers in order of the FF energy often generates duplicate results; using CONFPASS, the duplication rate is reduced by a factor of 2 for the first 30% of the re-optimizations, which include the global minimum structure about 80% of the time.

## Introduction

**Figure 1:**
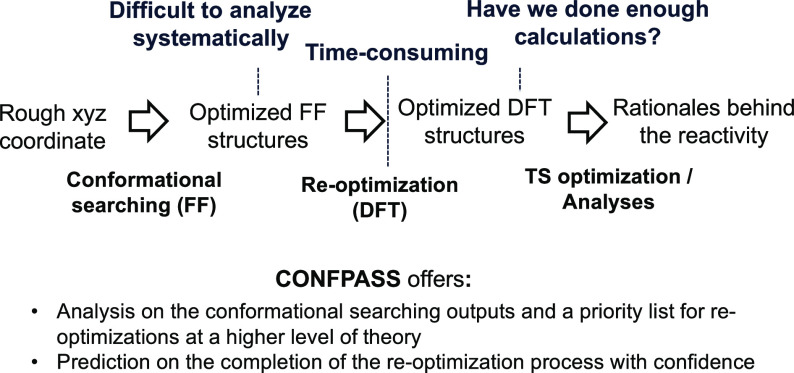
Computational workflow to understand chemical reactivities.

Molecular structure and conformational space are important to organic chemists in understanding the structure and the reactivity of a molecule.^1,2^ How many conformations do we need to consider to get a clear idea of the properties of a molecular system? This is central to the analysis of structure and reactivity. Recently, the potential of conformational flexibility as a tool in controlling the yield of the desired product and designing better catalysts has also been explored.^3^ Several reports have highlighted the benefits of conformational flexibility in catalyst designs, which include enhancing non-covalent interactions and improving substrate generality.^4−6^ However, the reactivity of a conformationally flexible molecule is difficult to investigate due to the vast conformational space and the mechanistic complexity.^7−9^ Computational analyses are valuable in exploring the chemistry behind the conformational flexibility prior to expensive experiments (Figure 1).^10−13^

To understand the reactivity of a structurally complex and flexible molecule, computational chemists often start with conformational searching calculations using a fast method, such as a force field (FF). The FF structures are re-optimized using density functional theory (DFT), which is more accurate but comparatively slow. Further analyses, such as transition state (TS) optimizations and non-covalent interaction analyses, can then be conducted to rationalize the reactivity and selectivity.^14^

DFT optimizations of the FF level structures are a bottleneck in this process. Conformer generators, such as MacroModel,^15^ OMEGA,^16,17^ and RDKit,^18^ can analyze the results of conformational searches and remove duplicate structures and high-energy conformers.^19−21^ The search for even better approaches is ongoing. CREST (Conformer-Rotamer Ensemble Sampling Tool)^22,23^ is a state-of-the-art program that performs conformational searching calculations with the iterative meta-dynamics with genetic crossing (iMTD-GC), based on a semi-empirical method (GFN2-xTB). At the end of the process, conformers are identified from rotamer ensembles based on root-mean-square deviation (RMSD), energy, and rotational constant thresholds. Even using such procedures, the conformational searching output files of structurally flexible molecules may include hundreds of relevant structures. Optimizing all the conformers from the output files at the DFT level requires a huge amount of computational time. Sometimes, distinct FF structures may end up having the same conformation at the DFT level. However, re-optimizing just a few conformers with low FF energy is also insufficient. Thorough explorations of the conformational space are critical for understanding reactivities. The correlations between the FF or semi-empirical potential energy surface (PES) and the DFT PES are difficult to predict for individual molecules, which have been reported in benchmarking studies.^24−26^ As is well known, a relatively high energy conformer (e.g., Δ*U*(FF) = 4 kcal mol^–1^) at the FF level might be the global minimum at the DFT level.

To take a simple example: a molecule with 10 FF conformations. If the lower-energy five are re-optimized using DFT, is it necessary to re-optimize the other conformations? In this paper, we present CONFPASS (**Conf**ormer **P**rioritizations and **A**naly**s**i**s** for DFT re-optimizations) a program that provides an answer to this question.

**Figure 2:**
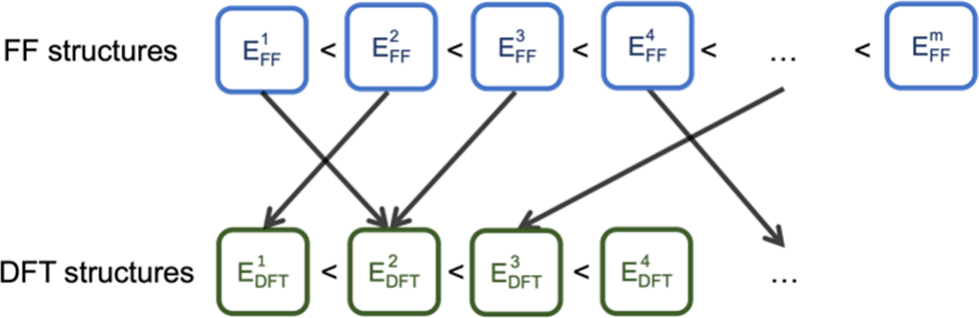
Sometimes, FF structures ordered by energy are re-optimized at a DFT level to a different order and a different number of structures.

CONFPASS starts with the output from an FF-based conformational searching program, with the conformers sorted in order of increasing FF energy. If these are all re-optimized at the DFT level (Figure 2) to generate a new list, ordered by DFT energy, the new list may well contain fewer structures, as different FF structures may optimize to the same DFT structure. While structures with high FF energies may re-optimize to the structure with energies closer to the DFT global minimum, structures with very high FF energies may be safely discarded.

Several strategies can be used for deciding on the order for DFT re-optimization. A simple but time-consuming approach is to re-optimize the structures according to the energy at the FF level from the lowest to the highest. We call this the *ascent approach*. This can be made faster by just selecting every *n*th conformer from the FF output file, the *every n*th *approach*. When *n* = 1, this is the same as the *ascent approach*. We can also randomly pick a sample of conformers for re-optimizations, the *random approach*. For large and flexible systems, the last two approaches risk missing out key parts of the conformational space. Several studies have proposed methods to balance speed with confidence in the outcome. For example, Johannessen and co-workers process conformational sampling outputs with principal component analysis and clustering algorithm to assist Raman optical activity spectral analyses of vancomycin, a glycopeptide.^27^ Merz and co-workers have developed AutoGraph, which clusters conformers and produces conformational graphs for metabolites, such as oxidized nicotinamide adenine dinucleotide.^28^ We previously built an automated pipeline to select representative conformers for DFT re-optimizations using a conformation labeling system based on dihedral angle values specifically aimed at studying the reactivity of diarylprolinol silyl ether catalysts.^29^ CONFPASS builds on our earlier approach to generate a general pipeline for diverse organic structures.

Our new procedure, CONFPASS, chooses the optimal order in which to re-optimize the FF structures based on a clustering approach and then assesses how much confidence can be had in incomplete calculations as the re-optimization progresses. Thorough evaluations based on a dataset of structurally diverse molecules have shown that CONFPASS is effective in assisting organic chemists explore conformational space and structures.

## Methodologies

### Data Gathering

This study is based on a set of 822 molecules, 711 of which come from a dataset gathered by Hutchison and co-workers.^30^ An additional 91 molecules are hydrocarbons derived from the Hutchison molecules by substituting heteroatoms with carbon atoms. Twenty molecules are taken from the work of Grayson and co-workers.^24^ The Grayson dataset molecules are mostly organic catalysts with hydrogen-bonding donating characteristics. Additionally, a small number of molecules, including three radical structures, come directly from the literature.^31−34^ The structures are listed in the SI. Conformational searching calculations were conducted on the 822 molecules in MacroModel (v11.7)^15^ with the Merck molecular force field (MMFF).^35^ Conformers with energies more than 10 kcal mol^–1^ above the global minimum were omitted. For radicals, ground state pseudo-structures were used for conformational searching calculations.

**Figure 3:**
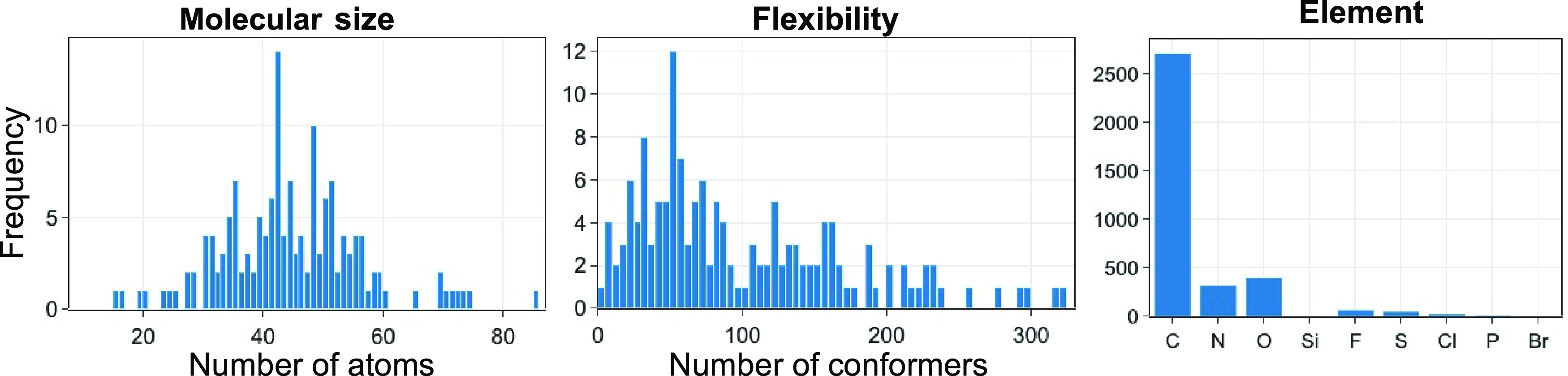
Profile of the 150 molecules in the DFT dataset. The 150 molecules include the 20 Grayson dataset molecules, 3 radical molecules, and 127 randomly picked molecules from the Hutchison dataset. The histograms show that the 150 organic molecules are generally flexible and structurally diverse in terms of molecular size.

Out of the initial pool of 822 molecules, a subset of 150 was selected for the construction of the DFT dataset (Figure 3; Table S1). The 150 molecules include the 20 Grayson dataset molecules and 3 radical molecules. The remaining 127 molecules were randomly selected. Out of the 150 molecules, 22 are charged, with 2 of them being zwitterions. For this subset, we re-optimized all the FF structures from the conformational searching calculations at the DFT level. DFT optimizations were performed with Gaussian 16 (Revision B.01).^36^ Here, the B3LYP-D3/6-31G(d) level of theory was chosen for the optimization and frequency calculations.^37−40^ Single-point energy calculations were conducted at the ωB97X-D/6-311G(d,p) level of theory.^41^ Unless specified, the “DFT level” in the text below refers to this level of theory. The 150 molecules are diverse in terms of molecular size and flexibility. The total number of conformers ranges from 2 to 321, and the average number of atoms is 44. About 15% of the re-optimized structures were found to be duplicates.

### Generating the Priority List

Dihedral angle data for each conformation are used as descriptors for the clustering process.

#### Dihedral Angle Descriptors

A list of bonds and bond order matrixes with and without H atoms are extracted from each structure. Bonds under the following categories are eliminated:

A. Bonds to monovalent atoms (F, Cl, Br, I, H);
B. Bonds to CX_3_ groups (X = F, Cl, Br, I, H);
C. Bonds in small rings and with a bond order >1.

The details of the bonds in group C are given in the SI. For these bonds, the dihedral angle (θ) values across all the conformers should have a small standard deviation (σ) and range (η) value. The σ and η parameters were calculated for 9017 sets of dihedrals with a bond order >1 in the center bond. The 9017 sets of dihedrals were identified using RDKit from 711 molecules in the Hutchison dataset.^30^ Box plot analyses were performed on the statistical parameter, and the outliers were inspected (Figures S6 and S7). Certain dihedrals with bonds containing heteroatoms or neighboring to heteroatoms with conjugated characteristics may have abnormally large σ and η. Therefore, these selective bonds are retained during the bond elimination step.

The remaining bonds are used to generate a list of dihedral angles, by selecting additional atoms, preferring heteroatoms to carbon and carbons to hydrogen.

The elimination criteria do not cover all possible fixed dihedrals. For example, dihedrals within the fused ring and most of dihedrals with conjugated C–X (X = heteroatoms) bonds are fixed. For each dihedral in the list of potentially rotatable dihedrals, dihedral angle values (θ) are calculated for every conformer. The standard deviation and range parameter of these dihedral angle values (σ(θ) and η(θ)) are also computed (Table 1). For dihedrals with values of θ close to ±180°, the standard deviation and range are computed from absolute θ (i.e., σ(|θ|) and η(|θ|)). If these parameters are within the limits given in Table 1, the dihedral is then labeled as fixed and the associated dihedral angle data are not used as a descriptor.

**Table 1:** Criteria for Identifying Fixed Dihedrals Based on Standard Deviation (σ) and Range (η) Parameters of Dihedral Angle Values (θ) and Absolute Dihedral Angle Values (|θ|)^a^

η(θ)	σ(θ)	η(θ)	σ(|θ|)	η(|θ|)
<300°	<8.7°	<2.4°		
≥300°	>359.2°		<6.6°	<1.5°

a The parameters were derived from box plot analyses on 7262 sets of dihedrals, which satisfy the criteria in C. fixed bonds (Figure S7). Dihedrals with a η(θ) ≥ 300° are identified as trans-oriented dihedrals.

Finally, a post-processing procedure is introduced to remove the artifact of θ values as a 1D measurement. A 179° and a −179° dihedral may be numerically different but are structurally similar. In these cases, the θ values from the negative quadrant are shifted to join up with the data points in the positive quadrant. The process ensures the continuity of θ values within the same cluster (Figure S9).

The output of the above analyses and calculations is an array of dihedral angle data, ready for the clustering process.

#### Clustering Algorithms

The clustering process is performed with the scikit-learn packages.^42^ The pipeline was initially developed using the K-means clustering algorithm. After finalizing other parts of the pipeline, a benchmarking test was performed. Several clustering algorithms were tested using the default hyperparameters, including mini batch K-means clustering, agglomerative clustering, BIRCH, and Gaussian mixture. Overall, changes in the clustering algorithms do not alter the performance significantly (Figure S10). However, the time required is noticeably different between clustering methods. The pipeline using the agglomerative clustering algorithm requires the least computational resource. The average time from extracting descriptors to the end of the clustering process is approximately a second (Figure S11). Hyperparameter tuning on the agglomerative clustering process was performed. Combinations of the linkage criterion and affinity metrics were tested in a grid searching style. Altering the hyperparameters does not change the result significantly (Figure S12). The agglomerative clustering method with the default hyperparameters (affinity = Euclidean and linkage = Ward) was chosen for the clustering approach pipeline. The inputs required are the list of dihedral angles and *n_clusters*, the number of clusters that are required, which we discuss in the next section.

#### Priority List Generation Methods

**Figure 4:**
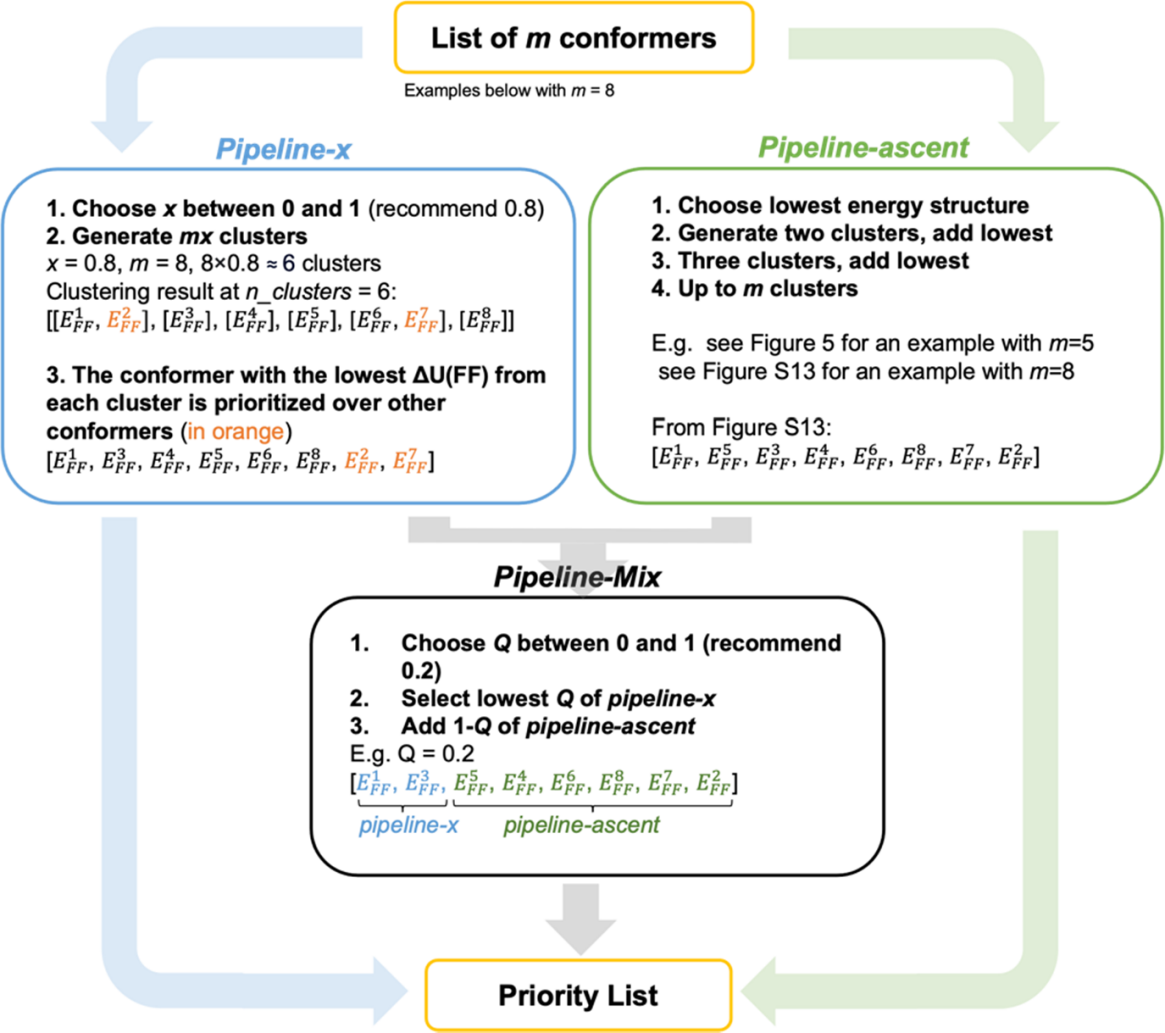
Different methods for generating priority lists from the clustering results. *m* is the total number of conformers in the conformational searching output file.

We are now ready to select conformations from the FF-re-optimized list for re-optimization by DFT methods and order them into a priority list for re-optimization. Three different approaches were used: *pipeline-x*, *pipeline-ascent*, and *pipeline-mix* (Figure 4).

The conformer with the lowest Δ*U*(FF) from each cluster is prioritized over other conformers. For the first of these, *pipeline-x*, the *n_clusters* value is chosen as a fraction, *x*, of the number of conformers. Too small a value for *x* would group diverse structures together; too large a value would ignore similarities between structures. A good choice of *x* might lead to a distinct cluster for each unique DFT conformation. Since 85% of the re-optimized structures in our dataset are unique, we selected a slightly smaller value, 80% of the total number of conformers, as a good choice for *n_clusters* (*x* = 0.8).

**Figure 5:**
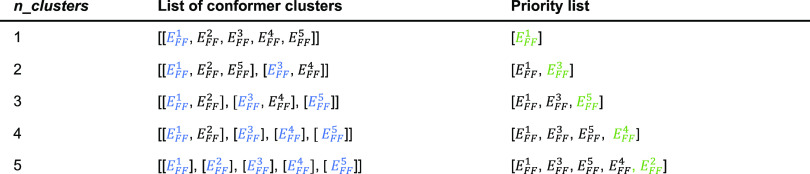
Illustration of the *pipeline-ascent* priority list generating method for a molecule with five conformers: from the clustering result (i.e., lists of conformer clusters) to the final priority list. At each new value of *n_clusters*, we add the lowest-energy structures from every cluster without a member on the priority list. The blue coloring indicates the most stable conformer from each cluster by FF energy in the list of conformer clusters. The green coloring indicates a conformer that has not appeared before and is added to the priority list at the corresponding *n_clusters* value.

For the *pipeline-ascent* method (Figures 5 and S13), the clustering process is repeated with all possible values of *n_clusters* from one to the total number of conformations (*m*), which gives *m* list of conformer clusters. From clustering results with *n_clusters* = 1 to *n_clusters* = *m*, the lowest-energy conformers from every cluster are introduced into the priority list while omitting those that are already in the priority list.

The *pipeline-mix* method involves truncating the *pipeline-x* and *pipeline-ascent* priority list. The *Q* parameter is the ratio of the *pipeline-x* content in the new *pipeline-mix* priority list. A new priority is assembled by picking the lowest *Q* of the *pipeline-x* priority list. Then, the list is completed in order of the *pipeline-ascent* priority list while omitting conformers that have already been chosen.

In this report, the clustering approach with the *pipeline-x* method is referred to as the *pipeline-x approach*. A similar logic applies to the *pipeline-ascent* and *pipeline-mix approaches*.

The above sections describe the procedure of CONFPASS in generating the priority list for conformer re-optimizations at a higher level of theory.

## Results and Discussion

### Evaluating CONFPASS Priority Lists

#### Evaluation Methods

##### DFT List

We utilized the DFT dataset, where all FF structures were re-optimized at the DFT level, to test and investigate how close CONFPASS gets to the complete analysis after re-optimizing only a fraction of the FF structures. FF structures that lead to duplicate conformers at the DFT level should be least prioritized.

**Figure 6:**
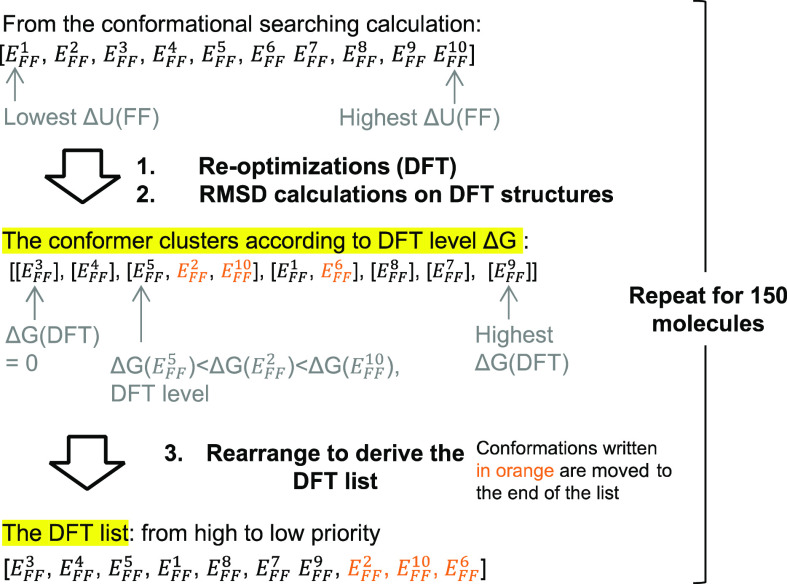
Process flowchart for obtaining the DFT list for evaluation. In the above example, conformers *E*_FF_^5^, *E*_FF_^2^, and *E*_FF_^10^ have the same structure after re-optimization at the DFT level with RMSD values less than 0.005 Å. Conformer *E*_FF_^5^ has the lowest Δ*G*(DFT) among the three and thus is prioritized over conformers *E*_FF_^2^ and *E*_FF_^10^. The same logic applies to the [*E*_FF_^1^, *E*_FF_^6^] cluster.

A workflow was built and used to derive the DFT list from the conformational searching output of a molecule (Figure 6). The conformers are given an index according to the internal energy at the FF level (Δ*U*(FF)). First, DFT optimizations were conducted on all conformers in accordance with the DFT dataset described in “Methodologies: Data Gathering”. RMSD calculations were performed with the GetBestRMS function under the rdkit.Chem.rdMolAlign module to compare the DFT structure on all non-H atoms of each pair of conformers.^18^ If the RMSD value is less than 0.005 Å, the pair of conformers would be considered as having the same structure. DFT conformers with the same structure were grouped into the same clusters. The clusters and the conformers within the clusters were arranged based on their Δ*G* values at the DFT level (Δ*G*(DFT)), which led to conformer clusters at the DFT level. The range of Δ*G*(DFT) within a cluster is usually less than 0.1 kcal mol^–1^. The DFT lists were constructed from conformer clusters at the DFT level. In the DFT lists, conformers with the lowest Δ*G*(DFT) within each cluster were prioritized over others. The above process was repeated for 150 molecules in the DFT dataset.

Proposed priority lists from FF structures were compared to the DFT list and conformer clusters at the DFT level, for evaluation. The global minimum test and bins test were established and conducted to measure how well priority generation approaches prioritize the most stable conformers and unique conformer structures, respectively.

##### Global Minimum Test

**Figure 7:**
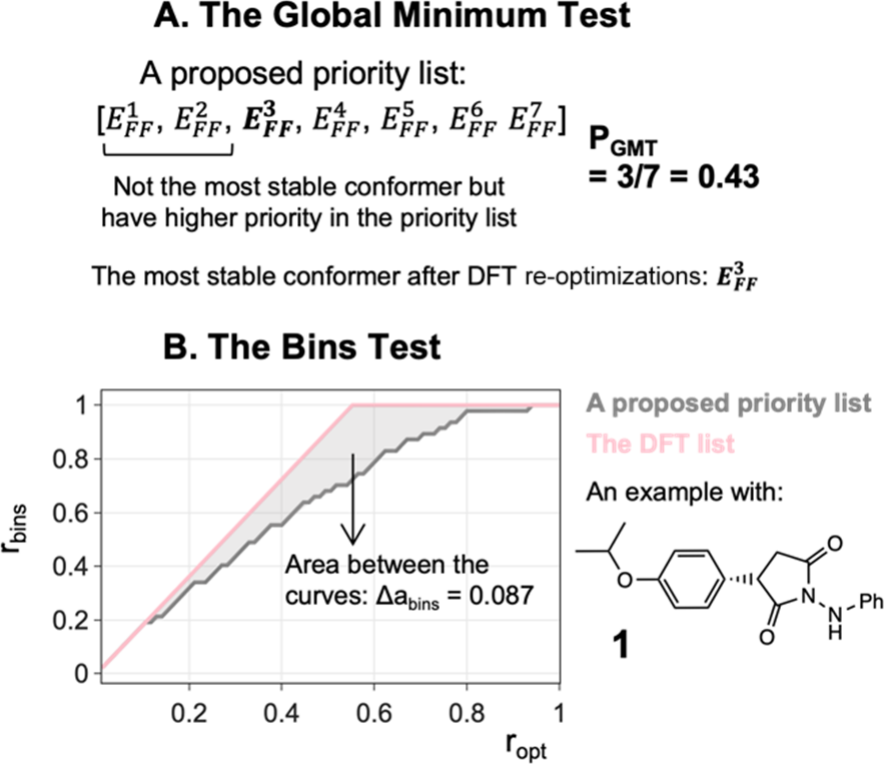
Tests for evaluating the performance of priority generation approaches in prioritizing the most stable conformer ((A) the global minimum test) and unique conformer structures ((B) the bins test).

The ratio of the number of DFT re-optimized conformers to the total number of conformers at the FF level is called *r*_opt_. CONFPASS tries to minimize the value of *r*_opt_, which corresponds to finding the global minimum. We call this value *P*_GMT_ (Figure 7A).

##### Bins Test

Each distinct structure at the DFT level is put in a separate “bin”. The bins test, Δ*a*_bins_, is a measure of how well the priority list avoids prioritizing duplicate conformers. The 150 molecules in the DFT dataset have 15,342 conformers at the FF level. In the 15,342 re-optimization calculations at the DFT level, 2280 (or 14.9%) did not lead to a unique structure. There are 13,062 unique structures, thus 13,062 bins, in the complete dataset.

The number of populated bins for a particular selection of conformers from partial optimizations divided by the total number of distinct structures at the DFT level is labeled *r*_bins_ (Figure S15). A plot of *r*_bins_ vs *r*_opt_ (Figure 7B) shows how *r*_bins_ increases with *r*_opt_, the fraction of the total number of structures that have been re-optimized. For the optimal order, *r*_bins_ should increase linearly with *r*_opt_ and plateau at *r*_bins_ = 1. The plot of *r*_bins_ vs *r*_opt_ for a proposed priority list is always under the line of the DFT list. The bins test, Δ*a*_bins_, is the area between the optimal order and the actual result in an *r*_bins_ vs *r*_opt_ plot. A proposed priority list with a small Δ*a*_bins_ value is desirable as conformers with unique structures are prioritized and duplicate conformers are avoided like in the DFT list. CONFPASS tries to minimize Δ*a*_bins_.

##### Overall Test

The overall parameter (*P*_overall_, eq 1) accounts for both the global minimum test (*P*_GMT_) and bins test (Δ*a*_bins_) parameters.
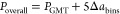
1

Theoretically, Δ*a*_bins_ can take up a value from 0 to 1. Preliminary studies show that 98.6% of the Δ*a*_bins_ values sampled are less than 0.2 (Figure S16B), which is also in line with the percentage of duplicate structures in the DFT dataset, 14.9%. Hence, Δ*a*_bins_ is scaled by a factor of 5. A low overall parameter value is desirable as this implies a good performance of the proposed priority list in prioritizing the global minimum and unique conformations.

We conducted hyperparameter tuning tests to validate our choice of default *x* and *Q* values with *P*_overall_. Analyses (see Figure S14) showed that the best performance comes when *x* = 0.8 and *Q* = 0.2 for the *pipeline-x* and *pipeline-mix* approaches.

#### Results

**Figure 8:**
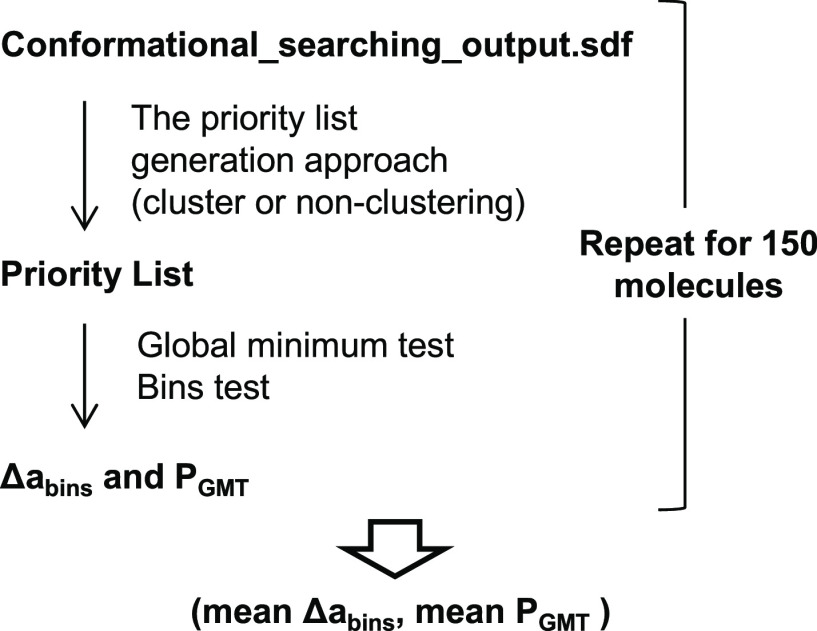
Workflow for evaluating pipeline and non-pipeline priority list generation approaches using the DFT dataset.

A standard procedure has been followed to compare the effectiveness of different priority list generation approaches (Figure 8). First, a priority list was generated from analyses of the conformational searching output file with non-pipeline or pipeline approaches. The proposed priority list was compared with the DFT list via the global minimum test and bins test, where *P*_GMT_ and Δ*a*_bins_ were calculated. The above process was repeated for the 150 molecules in the DFT dataset. A pair of (mean Δ*a*_bins_, mean *P*_GMT_) can be obtained for every priority list generation approach.

The mean *P*_GMT_ vs mean Δ*a*_bins_ plot illustrates the difference in performance between pipeline and non-pipeline approaches (Figure 10).

In the non-pipeline approaches, the *random approach* gives a lower mean Δ*a*_bins_ and performs better in avoiding prioritizing duplicate conformers compared to the *every n*th *approach*. However, the *every n*th *approach*, particularly the *ascent approach* (*n* = 1), gives a better chance of obtaining the global minimum with fewer re-optimizations. Increasing the *n* value reduces the global minimum test performance but improves the bins test performance.

**Figure 9:**
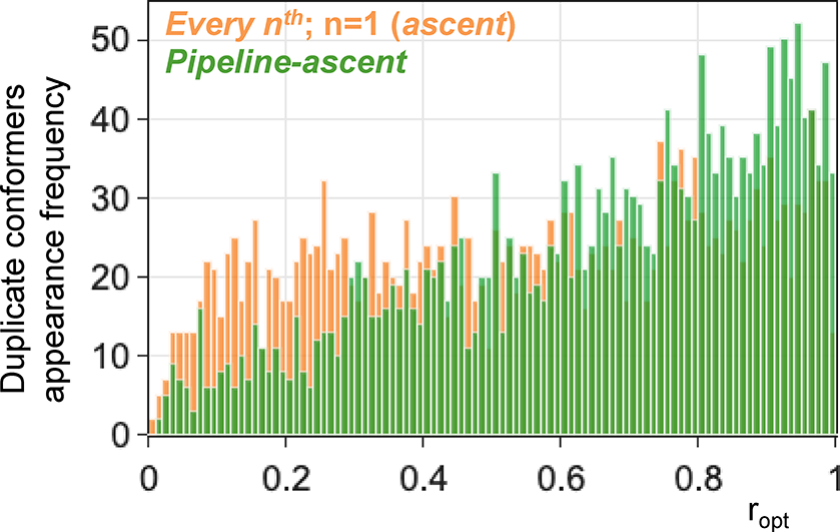
Histogram of duplicate conformer appearance frequency over *r*_opt_. The *pipeline-ascent approach* arranges the duplicate conformers toward the back of the priority list.

In the pipeline approach, the *pipeline-ascent approach* has the best bins test performance compared to all other approaches. Δ*a*_bins_(*pipeline-ascent*) is almost halved compared to Δ*a*_bins_(*ascent*). Additional histogram analyses of duplicate conformer appearance frequency over the re-optimization process in terms of *r*_opt_ have been conducted (Figure 9). The chance of getting duplicate conformers with the *pipeline-ascent* priority lists is approximately halved during the first 30% of the re-optimizations compared to the *ascent approach*. The mean *P*_GMT_ of the *pipeline-x approach* is similar to the *ascent approach*. Mean *P*_GMT_ = 0.14 implies that approximately 14% of the conformers in the priority need to be re-optimized before obtaining the global minimum structure. However, the mean Δ*a*_bins_ value of the *pipeline-x approach* is noticeably lower than the *pipeline-ascent approach*. The *pipeline-mix approach*, with the parameter *Q* set to 0.2, improves the bins test parameter without significantly reducing the global minimum test performance. With this setting, the global minimum structure can be obtained in the first 30% of the re-optimizations about 80% of the time (Figure S26).

We also investigated whether further steps improve the performance (SI Section 6.B). The aim is to extract and use the energy and structural data from the re-optimized conformers to improve the proposed priority list. The ideas that have been attempted include:

1) Sequential clustering process with certain FF structures replaced by the re-optimized DFT structures in the input SDF;
2) Varying the *x* parameter according to the percentage of structural duplicates in the group of re-optimized conformers when using the *pipeline-x* method;
3) Prioritizing conformers from the same clusters as the most stable DFT re-optimized structure;
4) Using clusters-distance array from the K-means clustering process to make the prioritization decisions.

**Figure 10:**
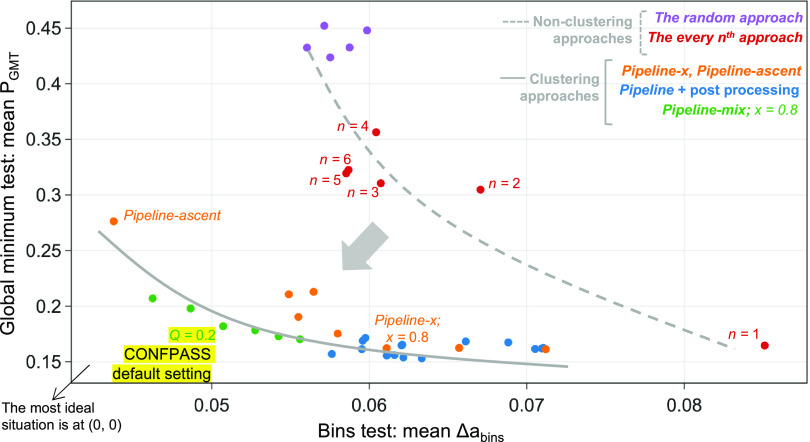
Global minimum test (*P*_GMT_) and bins test (Δ*a*_bins_) results are presented together in a scatter plot. The tests for the *random approach* have been repeated 5 times, which contributes to the 5 data points under this category. The corresponding hyperparameters (i.e., *n*, *x*, or *Q*) are labeled for selective approaches.

Overall, only marginal improvements in the global minimum test were observed (Figure 10, marked as post-processing). This demonstrates the effectiveness of the standard pipeline approaches.

**Figure 11:**
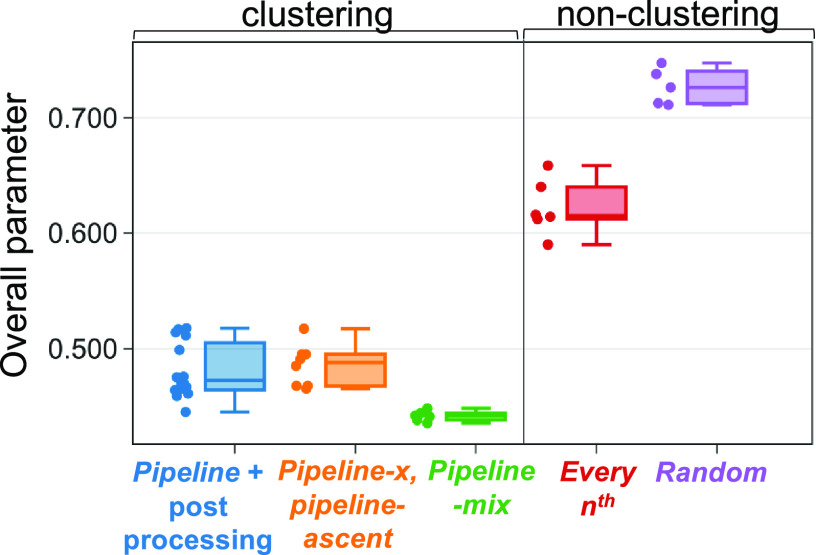
Overall parameter results for the pipeline and non-pipeline approaches are compared with the box plot.

Implementing the pipeline approach in generating the priority list led to a shift in data points toward the bottom left corner in the mean *P*_GMT_ vs mean Δ*a*_bins_ plot. The clustering process with dihedral angles as descriptors offered by the pipeline effectively groups conformationally similar conformers, which led to the noticeable improvement in the bins test parameter by the pipeline approaches. Differences in performance are also reflected in the mean overall parameter (Figure 11), calculated from the mean Δ*a*_bins_ and mean *P*_GMT_. The overall parameters for all other pipeline approaches are noticeably lower compared to the non-clustering approaches. Overall, the clustering approaches outperform the non-pipeline approach in prioritizing the global minimum structure and without duplications from different FF structures re-optimizing to the same structure.

If the aim of the study is to find the conformer with the global minimum energy at the DFT level, *pipeline-x* is the best approach. On the other hand, the *pipeline-ascent approach* should be used if only structural diversity is needed. This approach receives the lowest Δ*a*_bins_ in the bins test. Finally, the *pipeline-mix approach* should be used if the user wishes to avoid re-optimizing duplicate structures and wants to obtain a global minimum structure with less computational resources. The *pipeline-mix approach* with *x* = 0.8 and *Q* = 0.2 outperforms all other approaches in terms of the overall parameter and is chosen as the default setting for the clustering approach pipeline in CONFPASS.

The pipeline approach development, test, and evaluations are based on the MMFF to ωB97X-D/6-311G(d,p)//B3LYP-D3/6-31G(d) structural and energy data for 150 molecules. DFT datasets at other levels of theory are generated to test the generality of the clustering approach pipeline. Grayson and co-workers have conducted DFT re-optimizations with M06-2X functionals with an implicit solvent model (IEFPCM/benzene) on all the conformers on 20 organic molecules from conformational searching calculations with various FFs (incl. MMFF, MM2, and OPLS3e).^24^ The data from the work of Grayson and co-workers were processed and formatted for the tests. We also re-optimized the conformers of the 20 molecules at the expensive ωb97xd/6-311++g(d,p)//ωb97xd/6-31g(d) level of theory from the MMFF structures. In terms of the overall parameter, similar results were obtained when comparing the 4 subsets of 20 molecules and the extensive DFT dataset of 150 molecules (Figure S21). Additionally, we have repeated all the tests with Δ*H*(DFT), Δ*U*(DFT), and Δ*U*(B3LYP-D3/6-31G(d)//B3LYP-D3/6-31G(d)) values instead of Δ*G*(DFT). Using alternative sets of energy did not change result patterns, and the same conclusions can be drawn (Figure S24).

We recommend using the *pipeline-mix approach* with *x* = 0.8 and *Q* = 0.2, the default setting for CONFPASS. This usually gives a distribution of low-energy conformations and also finds the global minimum conformation quickly.

### Predicting the Completion of the Re-Optimization Process

Every DFT re-optimization gives us information about the conformational space of a molecule. Do we have enough information to decide we have gone far enough along the priority list and it is time to stop re-optimizations? To investigate this, we used our dataset of exhaustive DFT re-optimizations on FF conformation searches of 150 molecules (a total of 15,342 FF conformers) to build a machine learning model for answering the question.

As we re-optimize FF structures of a molecule following a priority list, we can calculate the mole amount relative to the current global minimum of the new conformer, χ_new_, for each new re-optimized structure based on all conformers re-optimized so far (eq 2; Δ*G*_new_ is the Δ*G* of the new conformer compared to the global minimum in the re-optimized structure so far).
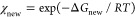
2

**Figure 12:**
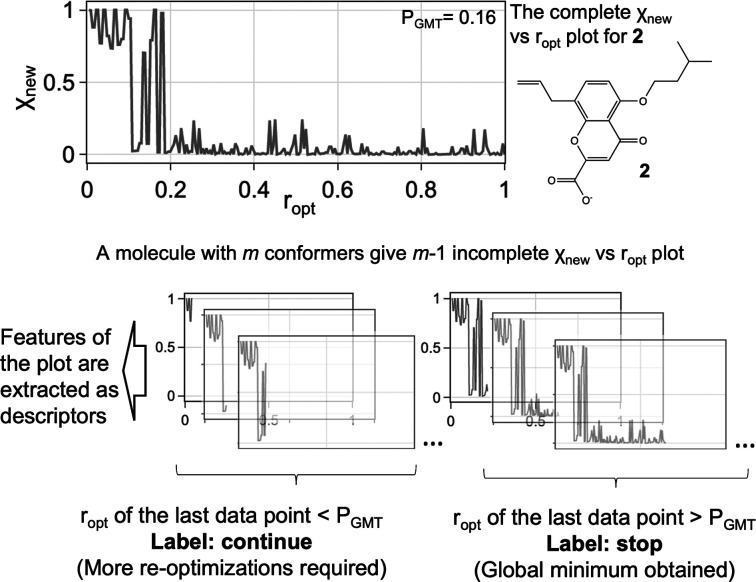
Mole amount of the last optimized conformer relative to the current global minimum (χ_new_) vs ratio of optimized conformer (*r*_opt_) plot. Each complete χ_new_ vs *r*_opt_ plot gives *m* sets of a descriptor array and label, where *m* is the total number of conformers from conformational searches.

If the new structure is a duplicate of an earlier one, we set χ_new_ to zero. We also know the proportion of the FF conformers that have been re-optimized, which we call *r*_opt_. After exhaustive DFT re-optimizations of every conformer, we can plot the χ_new_ against *r*_opt_ for the molecule (an example in Figure 12). The global minimum structure corresponds to the point with the highest *r*_opt_ value with χ_new_ = 1 on the complete χ_new_ vs *r*_opt_ plot.

How do we decide when to stop? With an exhaustive χ_new_ vs *r*_opt_ plot, we can determine *P*_GMT_, the value of *r*_opt_ at which the global minimum is found. Usually, however, we rarely re-optimize every conformer from the conformational searching output, so we do not know *P*_GMT_. Partial re-optimizations of the FF structures can provide an incomplete χ_new_ vs *r*_opt_ plot. We decided to employ a machine learning model to investigate if the data from an incomplete χ_new_ vs *r*_opt_ plot are sufficient to decide whether the re-optimization process structure can be terminated because the global minimum has probably been found.

Our DFT dataset can provide the data for the model training. In addition to the full χ_new_ vs *r*_opt_ plot, each molecule should give *m* – 1 incomplete χ_new_ vs *r*_opt_ plot, where *m* is the total number of conformers. The label (i.e., continue or stop) is assigned to each χ_new_ vs *r*_opt_ plot by comparing the *r*_opt_ value of the last data point to the corresponding *P*_GMT_. Features of a χ_new_ vs *r*_opt_ plot were extracted as descriptors:

1. Percentage of χ_new_ = 0, percentage of χ_new_ = 1, percentage of χ_new_ ≤ 0.1, percentage of χ_new_ ≤ 0.2, and percentage of χ_new_ ≤ 0.5 of the entire and the last 40% of the given χ_new_ vs *r*_opt_ plot.
2. The highest *r*_opt_ value at which χ_new_ = 1.
3. The *r*_opt_ value of the last data point in the plot.
4. The total number of data points in the plot.

The χ_new_ vs *r*_opt_ plot pattern depends on the order of the conformers in the priority list. Using priority lists generated under a specific setting, 150 molecules within the DFT dataset are expected to yield 15,342 descriptor arrays. We have considered nine different priority list generation approaches to generate as many χ_new_ vs *r*_opt_ plots as possible for building up the training data: *pipeline-x* (*x* = 0.8), *pipeline-ascent*, three sets of *pipeline-mix* (*x* = 0.8; *Q* = 0.15, 0.20, 0.25), and four sets of *every n*th (*n* = 1–4) priority list. This leads to 15,342 × 9 = 138,078 sets of a descriptor array and label. Given a list of χ_new_ and *r*_opt_ values for a partial re-optimization, should the optimization continue? We have the answer for the 138,078 examples from exhaustive re-optimization. Can this training set be used to generate a reliable guide to how many structures in a new conformation search need to be re-optimized?

We evaluated various classification methods from scikit-learn:^42^ random forest (RF), K-nearest neighbor classification (KNN), logistic regression (LR), and Gaussian Naive Bayes (GaussianNB). To assess the performance of the models in predicting the completion of the re-optimization process, we followed a rigorous cross-validation methodology. For each molecule, we took out the relevant data points associated with the molecule, which corresponds to less than 1% of the entire dataset. Models were trained on the filtered dataset. Input descriptor arrays derived from the default CONFPASS priority list generation approach (*pipeline-mix* with *x* = 0.8 and *Q* = 0.2) of the molecule were used for testing. Each molecule contributes *m* sets of prediction results from the designated model, where *m* is the total number of conformers. The above processes gave 15,342 prediction results for each classification method. The default hyperparameter settings were used. The LR model gives the highest accuracy, 0.88. The GaussianNB model has an accuracy of 0.86. KNN and RF models show similar performance with an accuracy of around 0.81. We also built a local model from χ_new_ vs *r*_opt_ plots based on the *pipeline-mix* priority list (*x* = 0.8 and *Q* = 0.2) by itself. The mean accuracy of the local model was very similar to that of the global model.

**Figure 13:**
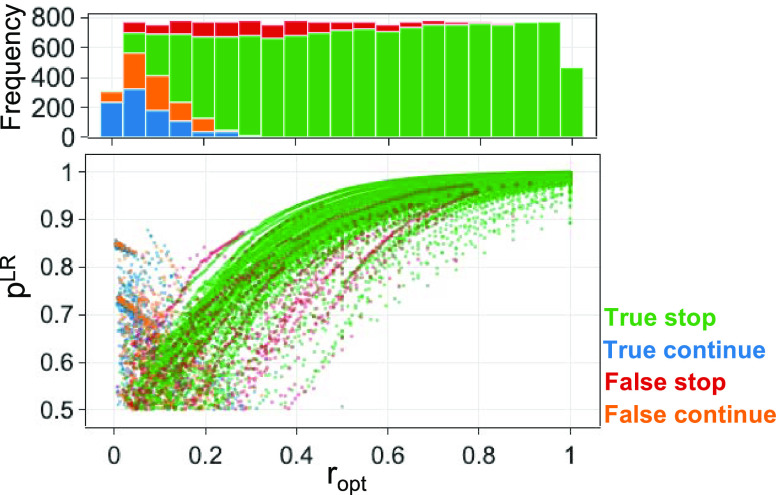
*p*^LR^ vs *r*_opt_ plot from the cross-validation test of the global LR model using input descriptor arrays derived from the default CONFPASS priority list generation approach. *p*^LR^ is the probability value from the LR model. A histogram is given for the true and false predictions by their *r*_opt_ values.

For the CONFPASS package, we have chosen the global LR model. The LR model generates a probability value, *p*^LR^, giving its confidence in each classification. Figure 13 presents a plot of *p*^LR^ vs *r*_opt_ from the cross-validation test of the global LR model. More than 50% of the false predictions have an *r*_opt_ value of less than 0.2. The outcome is within the expectation because the predictions rely on a descriptor array derived from a relatively limited amount of data.

Using the results from the cross-validation test, we found that the *p*^LR^ correlates with the percentage of true predictions in our dataset of 150 molecules (%Conf; i.e., percentage confidence to terminate the re-optimization process) via a sigmoid curve (eq 3; SI Section 7.C). The mean absolute error of the fit was 2.6%.
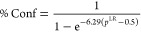
3

Given the conformational searching results at the FF level, a chosen priority list generation setting, and outputs of partial re-optimizations at the DFT level, CONFPASS generates the χ_new_ vs *r*_opt_ plot and suggests whether further re-optimization is needed with a confidence value, %Conf. All the false predictions above *r*_opt_ = 0.45 are attributed to instances where the model predicts “stop” when it should not. Thus, 1 – %Conf when *r*_opt_ > 0.45 is also equivalent to the percentage of false stop predictions.

**Figure 14:**
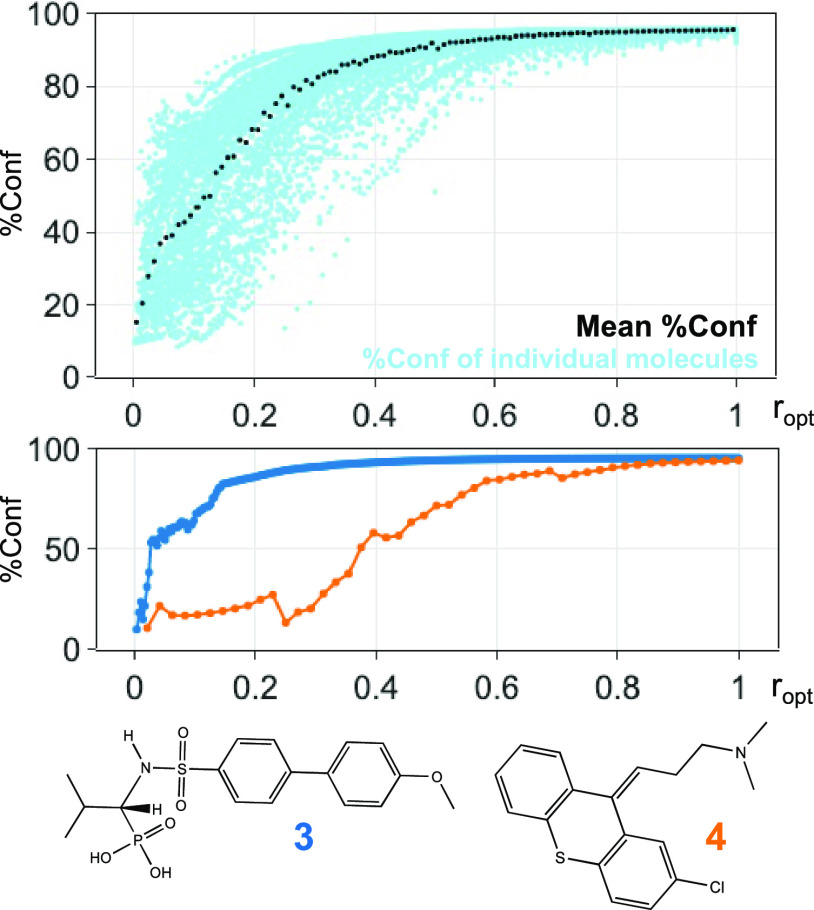
Percentage confidence (%Conf) vs *r*_opt_ plot. The light blue data points come from individual molecules in the DFT dataset. These data points were separated into 100 bins of equal size according to the *r*_opt_ values. The mean %Conf was found for each bin and plotted above in black. Examples of the %Conf vs *r*_opt_ plot for individual molecules are also presented.

We calculated %Conf values from the *p*^LR^ of the global LR model predictions from the cross-validation test. In Figure 14, the mean %Conf values from the test are plotted against *r*_opt_ for all 150 molecules. %Conf = 50% is the margin between stop and continue predictions. On average, 14% (*r*_opt_ = 0.135) of the conformers need to be optimized to achieve %Conf = 50%. When *r*_opt_ = 0.5 (50% optimized), the average %Conf is 91%. For the most flexible molecules in the dataset (i.e., molecules with >170 conformers), %Conf at *r*_opt_ = 0.5 is over 95% (SI Section 7.E). On average, %Conf no longer deviates below 90% after re-optimizing 47% of the conformers. When %Conf >90%, conformers contributing to approximately 86% of the Boltzmann distribution population should be covered (SI Section 7.E).

The DFT dataset comprises distinct molecules that vary in terms of structure and flexibility. Specifically, for certain molecules like **3**, a %Conf score of 90% is achieved by re-optimizing about 25% of the priority list. Conversely, for **4**, 70% of the conformers need to be re-optimized to exceed 90% %Conf.

### CONFPASS at Work

**Figure 15:**
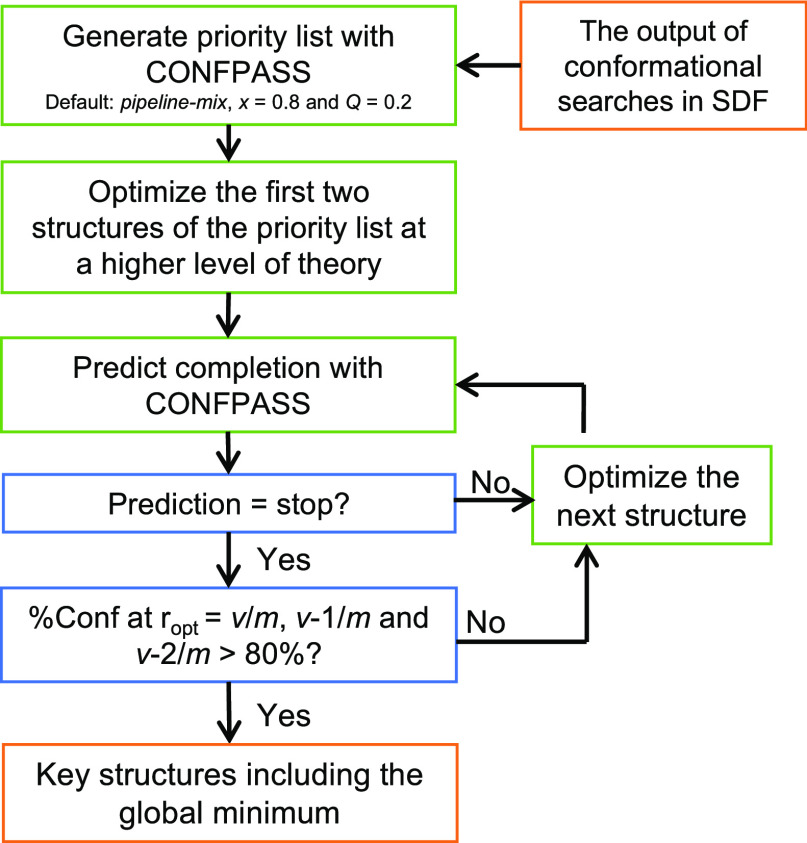
Usage recommendations of CONFPASS. *m* is the total number of conformers and *v* is the number of re-optimized conformers. We recommend that the cut-off for terminating the re-optimization process should be higher than 80%. Depending on the computer system, the user of CONFPASS may also wish to re-optimize FF structures in batches.

**Figure 16:**
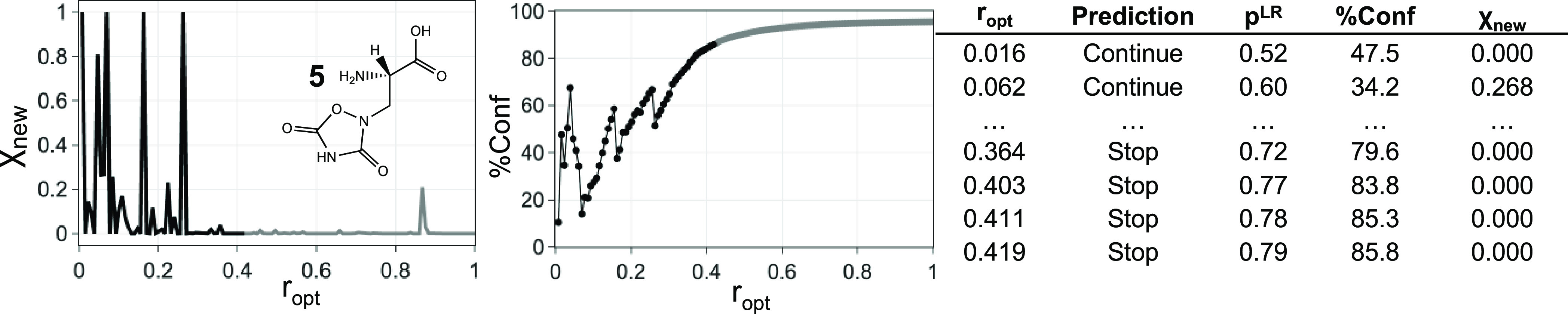
CONFPASS at work: applications of CONFPASS in exploring the conformational space of **5** from the Hutchison dataset. The black data points on the plots are known to the user at their wish after re-optimizing 42% of the conformers following the default CONFPASS priority list (*pipeline-mix*, *x* = 0.8 and *Q* = 0.2). The complete data table is presented in Table S12.

**5** is a molecule collected by Hutchison and co-workers but outside our DFT dataset. Here, we will use **5** as an example to demonstrate the application of CONFPASS in exploring the conformational space (Figures 15 and 16).

Conformational searches of **5** give 128 conformers. We generated a priority list with CONFPASS using the default setting (*pipeline-mix*, *x* = 0.8 and *Q* = 0.2) and re-optimized the first two structures of the conformers following the priority list. At *r*_opt_ = 2/129, the LR model predicts that further calculations are required. On our computer system, it is more convenient to re-optimize conformers in batches. Thus, the next 5% of the conformers from the priority list were sent for DFT re-optimizations. After incorporating the updated results, a new prediction is generated using the LR model. The above procedure is iterated until the %Conf of the newly generated prediction surpasses 80%. At *r*_opt_ = 0.42 (i.e., 54/129), the %Conf exceeds 80%, indicating that the prediction of achieving the global minimal structure is highly probable. We also determined %Conf for *r*_opt_ = 52/129 and *r*_opt_ = 53/129 using *p*^LR^ from the prediction. Both values are greater than 80%, suggesting that a reliable and consistent outcome has been achieved. The time required for DFT calculations can be reduced by running them in parallel or using cloud resources. This reduces the wall-clock time but not the computational resource requirement. CONFPASS makes it possible to use the available computational resources more effectively.

### Comparison with CREST

CONFPASS is designed for obtaining all low-energy structures at a higher level of theory with a confidence level that all relevant structures have been found. CREST^22,23^ is often used for conformational studies and has a different aim: to generate representative structures, which is very useful for predicting spectroscopically relevant structures and finding macroscopic properties.

We performed conformational searching calculations using CREST and re-optimized all the conformers from the output files at the DFT level for a random selection of 20 molecules from the master dataset (i.e., the CREST-DFT dataset). 11 of the 20 CREST-DFT dataset molecules overlap with the DFT dataset, which allows us to compare the performance between CREST and MacroModel as the starting point of DFT re-optimization processes. We compared DFT structures re-optimized from the CREST and MacroModel output for the 11 molecules in terms of the Boltzmann distribution population. In most cases, the same global minimum and low-energy structures were identified upon DFT re-optimizations from the CREST and MacroModel output. However, conformational searches with MacroModel were more than 8 times faster compared to CREST. Although more conformer structures were found with CREST compared to MacroModel on average, 53% of the DFT re-optimized structures in the CREST-DFT dataset were duplicate structures. This means that CREST is much slower overall because it has to do many more DFT optimizations to get to the same outcome. Re-optimizing all conformers from CREST searches needs 1.6 times more CPU time than re-optimizing all the conformers from MacroModel. Using CONFPASS to give greater than 90% confidence, with FF structures from MacroModel as the starting point, is more than 3.8 times faster than re-optimizing all conformers from CREST outputs (SI Section 8.D).

CONFPASS is flexible and able to accept conformational searching output files from any program that can generate SDF. Thus, CONFPASS can also accept output files from CREST. With the CREST-DFT dataset, the global minimum and bins tests were performed, and overall parameters were calculated by various priority list generation methods. Similar results were obtained compared to the result from the DFT dataset. We then ran tests with the LR model for predicting the completion of the re-optimization process with the CREST-DFT dataset. On average, CONFPASS gives more than 90% confidence after re-optimizing 42% of the conformers following the default priority list, which covers 90% of the Boltzmann distribution population (SI Section 8.C). The above investigation demonstrates the robustness of CONFPASS in accepting output files from calculations at different levels of theory and conformational searching methods. The prioritization and LR model prediction from CONFPASS can help obtain the global minimum and key low-energy structures with a reduced computational cost.

If the aim is to find representative DFT geometries, CREST is the appropriate choice. If you need the lowest-energy DFT structures and a confidence level that you have found them, then CONFPASS gives this information faster and more effectively.

## Conclusions

In conclusion, we have established a practical framework for analyzing conformational searching output and generating priority lists for re-optimizing conformers at the DFT level. The automated clustering approach pipeline extracts dihedral angle values as descriptors from conformers, performs the agglomerative clustering process, and generates the priority list. Thorough evaluations were conducted through the global minimum test and the bins test based on a large dataset of 150 structurally diverse molecules. The priority list from the pipeline intuitively guides the DFT re-optimization process and reduces the chance of getting duplicate conformers by up to 51% for the first 30% of the calculations. Machine learning models for predicting the completion of the re-optimization process were trained and evaluated. Users can finish their DFT re-optimization process at an earlier stage with the confidence that the global minimum has been obtained based on the *p*^LR^ value.

This project led to the development of the CONFPASS program, which covers the clustering approach pipeline for generating priority lists and tests for predicting the completion of the re-optimization process. CONFPASS is fully automated and offers rapid analyses. We have tested CONFPASS with FF structures from MacroModel and semi-empirical structures from CREST, which demonstrates the robustness of the program.

In the future, the CONFPASS program will be further developed to cope with organometallics and multimeric systems. Adapting the program for finding transition states is also an interesting aspect to investigate.

## Confpass

Based on the above studies, the CONFPASS program was developed to analyze conformational searching calculation outputs and guide the DFT re-optimizations. CONFPASS is fully automated, written entirely in Python and appliable for organic molecules, including radicals. The following packages were used: the Python (3.8.12) Standard Library, Pandas (1.3.4), Numpy (1.21.2), Sklearn (1.0.1),^42^ RDkit (2019.09.3),^18^ and Natsort (8.0.2). We also used the work of Jensen to convert xyz coordinates to a RDkit mol object.^43,44^

The CONFPASS program generates a priority list along with the input files to DFT calculations from the conformational searching result in SDF format. After partial re-optimizations of the FF structures at the DFT level, CONFPASS uses a machine learning model to predict if the global minimum structure has been obtained with a percentage confidence value.

The CONFPASS program is available to download on GitHub (github.com/Goodman-lab/CONFPASS), with a detailed user guide and examples. The scripts can be executed as a single command line or imported and used in Python scripts. The analyses are rapid and take on average few seconds to process an SDF or a Gaussian 16 output file folder.

## Supporting Information Available

CONFPASS program; computational methodologies; datasets; clustering approaches; performance metrics; evaluation; predicting the completion of the re-optimization process; using conformational searching outputs from CREST as the starting point; and additional figures and tables (PDF)

## Notes

All the data for this paper are available on Cambridge Apollo Repository: DOI: 10.17863/CAM.88197
