# Supplementary material for: CONFPASS: Fast DFT Re-Optimizations of Structures from Conformation Searches
Source: J Chem Inf Model. 2023 Jul 10;63(14):4364–75. doi: 10.1021/acs.jcim.3c00649 (PMC10369492; doi:10.1021/acs.jcim.3c00649)
Supplement: Supplementary file 1 — ci3c00649_si_001.pdf [file ci3c00649_si_001.pdf]

Supporting information

CONFPASS: fast DFT re-optimizations of structures from conformation searches

Ching Ching Lam[1] and Jonathan M. Goodman[1,*]

1. Yusuf Hamied Department of Chemistry, University of Cambridge, Lensfield Road, Cambridge, CB2 1EW
*jmg11@cam.ac.uk

Table of Contents

1. The CONFPASS Program 4
2. Computational Methodologies...7
3. The Data Sets 9
4. The Clustering Approaches..15
5. Performance Metrics 23
6. Evaluation 25
7. Predict the Completion of the Re-optimization Process 37
8. Using conformational searching outputs from CREST as the starting point...................46
9. Reference 57

Glossary

**DFT**	Density Functional Theory
**FF**	Force Field
**CREST**	Conformer-Rotamer Ensemble Sampling Tool
**CONFPASS**	Conformer Prioritizations & Analysis for DFT re-optimizations
m	Total number of conformers from conformational searches at FF level
n_clusters	Number of clusters
x	Key parameter in the pipeline-x method: *n_clusters*=*m***x*
Q	Key parameter in the pipeline-mix method: the ratio of the *pipeline-x* content in the new *pipeline-mix* priority list
**RMSD**	Root-mean-square deviation
$\mathbf{P_{GMT}}$	The proportion of conformers need to be optimized in order to obtain the most stable conformer at the DFT level
$\mathbf{r_{bins}}$	The ratio of populated bins for a particular selection of conformers from partial optimizations; the number of bins is the number of distinct structures at DFT level
$\mathbf{r_{opt}}$	The ratio of the number of DFT re-optimized conformers over the total number of conformers at FF level
$\mathbf{\Delta a_{bins}}$	The area between the DFT list and proposed priority list curve in a rbins vs ropt plot
$\mathbf{P_{overall}}$	$P_{overall} = P_{GMT} + 5(\Delta a_{bins})$
**B3LYP**	Becke exchange with Lee-Yang-Parr correlation
**B3LYP-D3**	B3LYP with D3 dispersion correction
**M06**	Minnesota 06 functional
**ωB97X-D**	Head-Gordon's Long-range corrected hybrid density functionals with dispersion corrections
$\boldsymbol{\chi}$	Mole amount; χ=exp(-ΔG/RT)
$\boldsymbol{\chi_{new}}$	Mole amount of the last optimized conformer
$\mathbf{r_{opt,conf=c}}$	The r_{opt} at which %Conf no longer deviates below c in the %Conf vs r_{opt} plot
**RF**	Random forest
**KNN**	K-nearest neighbor classification
**LR**	logistic regression
**GaussianNB**	Gaussian Naive Bayes
**θ**	Dihedral angle value
**σ**	Standard deviation
**μ**	Mean
**η**	Range
**%Conf**	Percentage confidence: equivalent to the percentage of true prediction

1. The CONFPASS Program

CONFPASS stands for '**Conf**ormer **P**rioritizations & **A**naly**s**i**s** for DFT re-optimizations' (available to download via: github.com/Goodman-lab/CONFPASS). The package is written entirely in Python and developed under the following environment:

- The Python (3.8.12) Standard Library (os, sys, itertools, collections, pickle, random, optparse, traceback, json)
- Pandas (1.3.4)
- Numpy (1.21.2)
- Sklearn (1.0.1)[1]
- RDkit (2019.09.3)[2]
- Natsort (8.0.2)

We used the script from https://github.com/jensengroup/xyz2mol/blob/master/xyz2mol.py (which is based on the work of Bull. Korean Chem. Soc. 2015, Vol. 36, 1769-1777) to convert xyz coordinates to a RDkit mol object.

This package is suitable for processing organic molecules, including radical species, and is currently under development for processing organometallics and inorganic compounds. More details can be found in the user guide, where examples are given on the execution of the package as a Python module. The package can also be imported and used in python scripts. Documentation and tutorials are provided in a Jupyter notebook. Example SDFs from conformational searching calculations and output file folders are also given.

The directory tree of the CONFPASS package (v19042023):

```
confpass/
├─ __init__.py
├─ __main__.py
├─ BinsTest_rmsCheck.py
├─ cal_dihedral_v2.py
├─ clustering_dih_v7.py
├─ confpass.py
├─ correcting_dihedral_v1.py
├─ dihedral_parameter_v2.py
├─ get_dft_output_v2.py
├─ GetPriority_v3.py
├─ isolate_key_dihedral_v5.py
├─ LR_model_24042023_x10.sav
├─ MolFrac_ML.py
├─ to_g16_input_v2.py
└─ xyz2mol.py
```

The directory tree of the user guides, SDFs and output file folders:

```
test_demo/
├─ CONFPASS_tutorial_v4.ipynb
├─ omegacsd_VORJER.sdf
├─ test_15.sdf
├─ test_15/
│   ├─ …(Gaussian .out files)
│   │   ├─ spe/
│   │   │   ├─ …(Gaussian _spe.out files)
├─ radical/
│   ├─ radical_gu_liu1/
```

```
│     │     ├─ …(Gaussian .out files)
│     │     ├─ spe/
│     │     │   ├─ …(Gaussian _spe.out files)
│     ├─ radical_gu_liu1.sdf
```

The architecture of the CONFPASS program:

A. Class conp

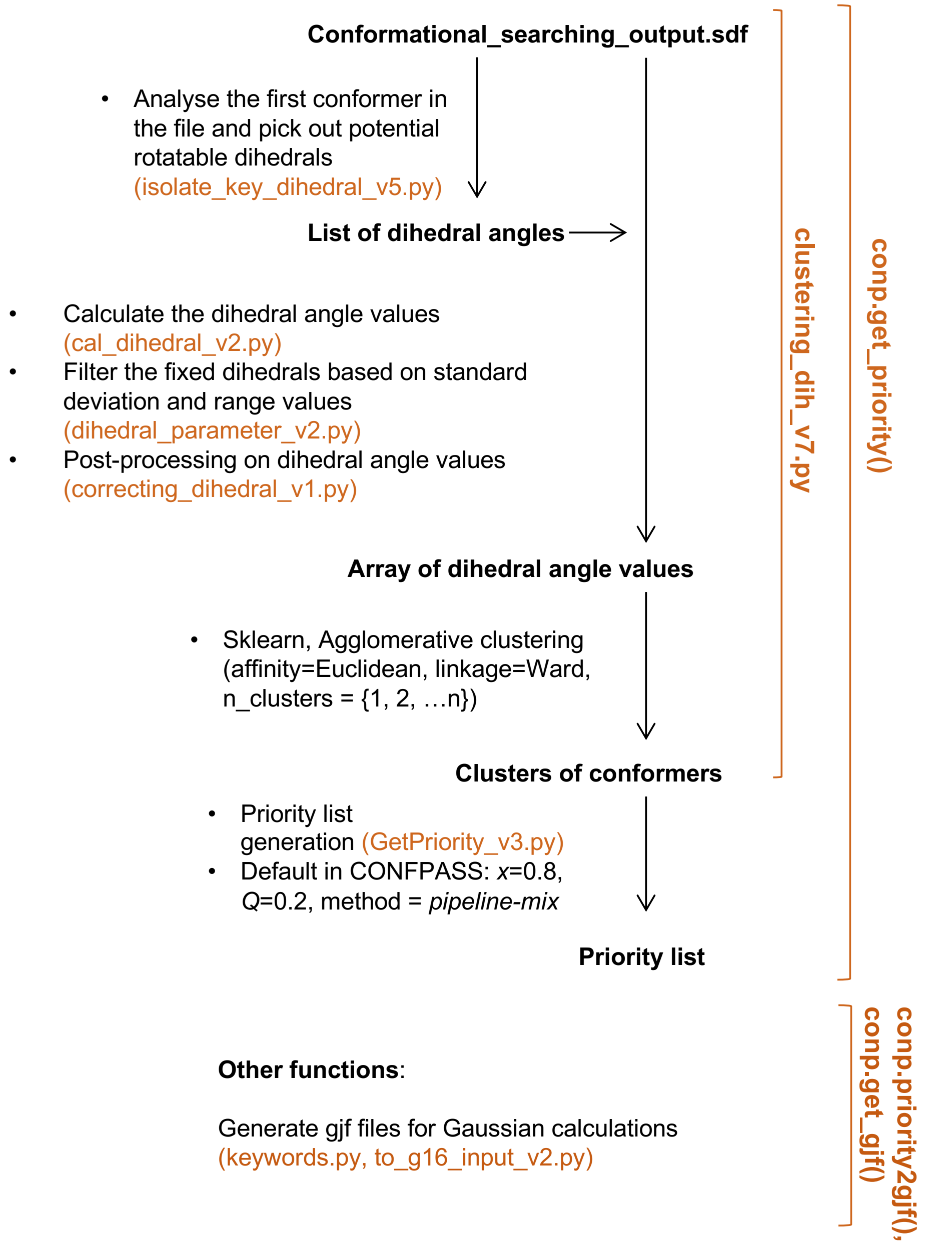

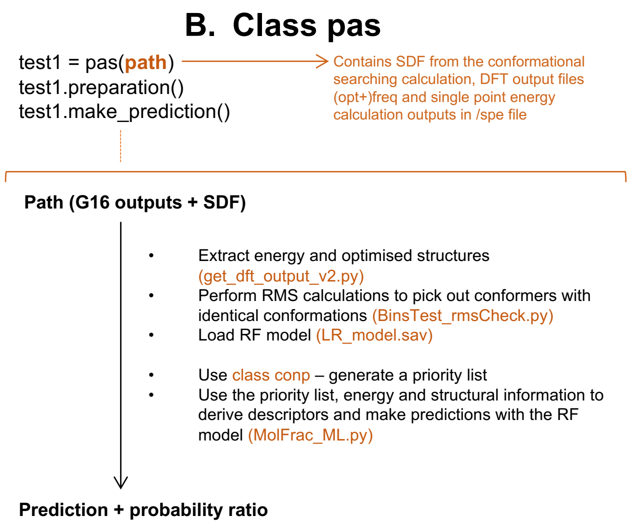

Figure S1. The architecture of the CONFPASS program. There are two parts in the package: A. class conp (pre-re-optimizations: clustering and priority list generation) and B. class pas (post-re-optimizations: predict the completeness of the re-optimization process)

2. Computational Methodologies

A. Conformational searching calculations

A standard workflow was followed when performing the conformational searching calculations for 822 molecules (Figure S2). Most of the steps in the workflow have been automated with python or bash scripts. Inputs to the workflow were SMILE strings and the outputs were the SDF and log file of the conformational searching process.

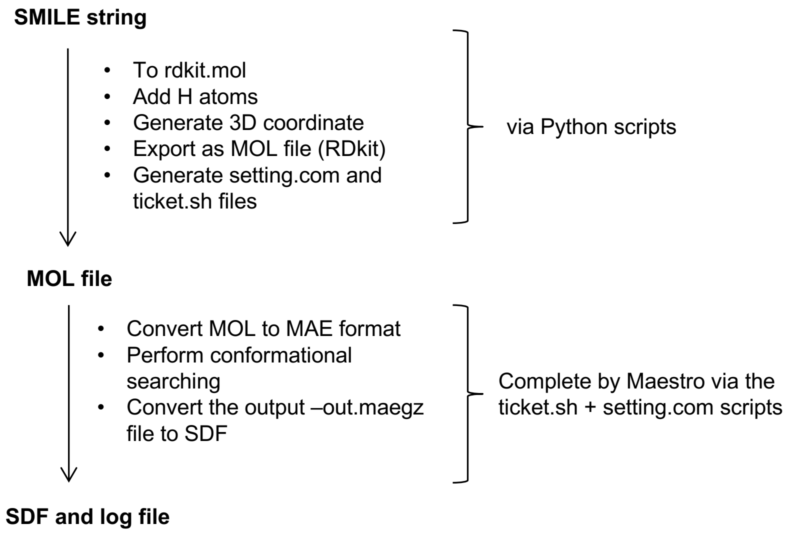

Figure S2. The workflow for conducting conformational searching calculations

Conformational searching calculations were conducted in MacroModel (v11.7) with MacroModel (release 2019-01).[3] The Merck molecular force field (MMFF)[4] was used with the mixed torsional / low-mode sampling method and a setting of 2000 steps as the maximum number of steps. Conformers within an energy window of 41.6 kJ mol^{-1} (i.e an equivalent of 10 kcal mol^{-1}) were saved for further analyses.

A conformational searching calculation was considered complete when the following criteria were met:
1. No new global minimum structure was found during the last 0.25*total number of steps (*ie* the last 500 steps in the case of a calculation with 2000 steps).
2. The unique structures over the total number of steps should be less than 0.7.

The above criteria can be checked via inspections of the log file. If the calculation were considered incomplete, conformational searching calculations would be repeated with the global minimum structure from the previous calculation and with an increasing number of steps (*ie* up to 4000 steps).

B. DFT calculations

For a selection of 150 molecules, all the conformers from the conformational searching calculations were optimized at the density functional theory (DFT) level. DFT-optimized structures were verified through frequency analyses. All the geometries were confirmed to correspond to a minimum on the potential energy surface (PES).

Here is a summary of the calculations and the associated levels of theory:
- MMFF → ωb97xd/6-311g(d,p)//B3LYP-D3/6-31g(d): the 150 molecules
- MMFF → ωb97xd/6-311++g(d,p)//ωb97xd/6-31g(d): the 20 molecules from the Grayson data set, which are part of the 150 molecules (*ie* the Grayson molecules)

In addition, we have also processed and formatted the existing data set from Grayson *et al.*[5] for benchmarking at different levels of theory.

- MMFF → M06-2X/def2-TZVPP/IEFPCM/benzene//M06-2X/6-31G(d)/IEFPCM/benzene: 20 Grayson molecules
- MM2→ M06-2X/def2-TZVPP/IEFPCM/benzene//M06-2X/6-31G(d)/IEFPCM/benzene: 17 Grayson molecules
- OPLS3e → M06-2X/def2-TZVPP/IEFPCM/benzene//M06-2X/6-31G(d)/IEFPCM/benzene: 20 Grayson molecules

We also evaluated the CONFPASS workflows by considering the CREST conformational searching program (SI Section 8):

- GFN2-xTB → ωb97xd/6-311++g(d,p)//ωb97xd/6-31g(d): the 20 randomly selected molecules from the master data set

C. Data analyses

Data analyses were conducted with Python (3.8.12). Graphs were created with Matplotlib (3.3.2) or Plotly (5.1.0).

Relevant scripts are available upon request.

3. The Data Sets

The conformational searching outputs and the DFT data set are available in the Apollo repository of the Cambridge University Library (DOI: 10.17863/CAM.88197). The 822 molecules with data at the FF level are diverse in molecular size and fixability (Figure S3). Other than the 20 Grayson molecules, the other 130 molecules in the DFT data set (Figure S4) were chosen randomly from the 822 molecules with a bias for the more flexible system. Properties of non-H atoms, including aromaticity and hybridization, are examined by RDkit and reported below as bar charts.

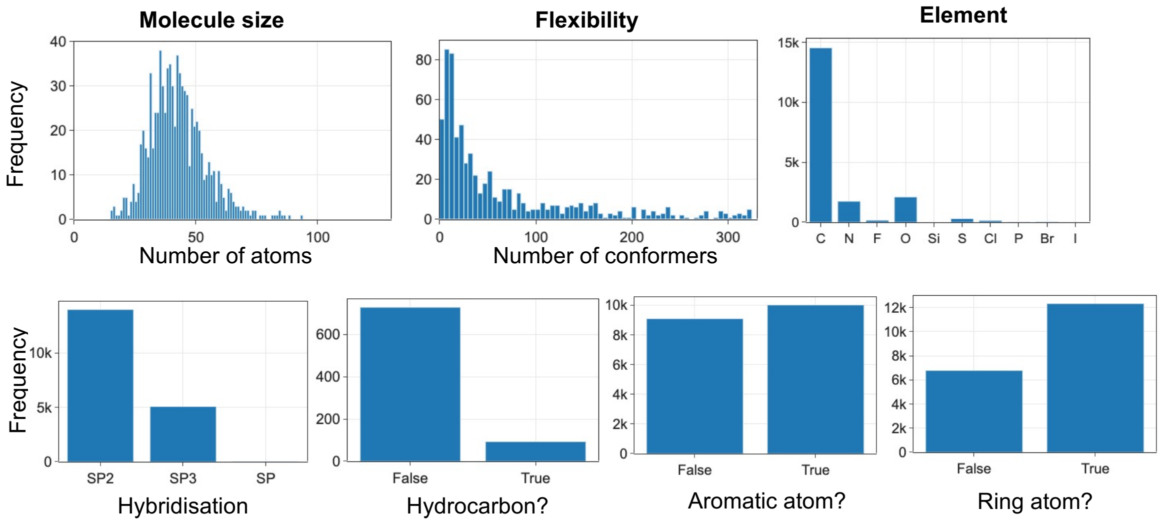

Figure S3. An overview of the 822 molecules in the conformational searching output data set. The bar charts summarize the nature of the atoms in the molecules.

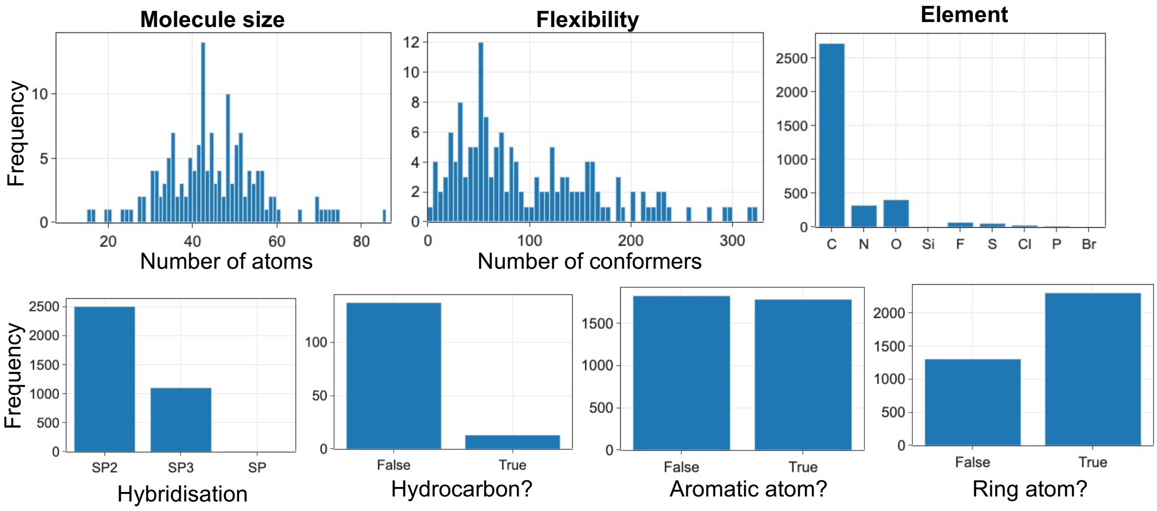

Figure S4. An overview of the 150 molecules in the DFT data set. The bar charts summarize the nature of the atoms in the molecules.

In the DFT data set, there are three radical molecules. The structure of the radicals and pseudo structures for conformational searching are given in Figure S5.

Name	Structure	Pseudo structure
radical_wong1[6]	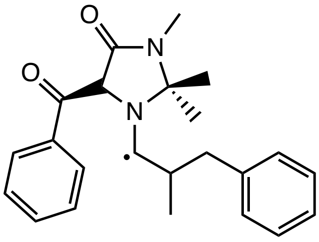	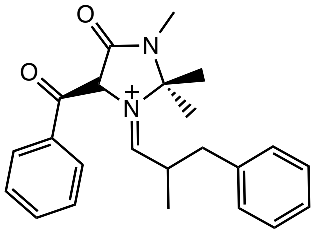
radical_gu_liu1[7]	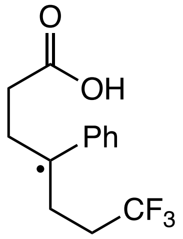	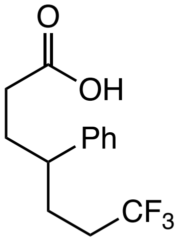
radical_baran3[8]	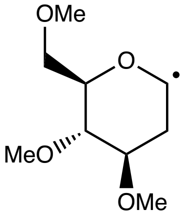	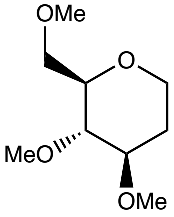

Figure S5. The structure and pseudo structure of the radical molecules in the DFT data set. The pseudo structures were used for the conformational searching as MMFF cannot cope with radical species.

Table S1. The SMILES representation of molecules in the DFT dataset

	Name	SMILES
0	omegacsd_WELGAV	Cc1nc(C)c2c(n1)N(Cc1ccc(-c3ccccc3-c3nnn[nH]3)cc1)C(=O)CC2
1	omegapdb_1h1p	Nc1nc(OCC2CCCCC2)c2nc[nH]c2n1
2	omegapdb_1xn0	COc1ccc([C@H]2CNC(=O)C2)cc1OC1CCCC1
3	grayson_cinchronidine	C[C@H]1C[N@@H+]2CC[C@H]1C[C@H]2[C@H](O)c1ccnc2ccccc12
4	omegapdb_2btr	CC(C)c1c[nH]/c(=N\C(=O)Cc2cccnc2)s1
5	omegacsd_JOTDAX	S=C(Nc1ccccc1)c1c(Nc2ccccc2)ss/c1=N\c1ccccc1
6	omegacsd_PMAANO01	CC1(C)C[C@](C)(N(O)c2ccccc2)ON1c1ccccc1
7	astex_1xm6	CCCOc1cc([C@]2(C)CNC(=O)O2)ccc1OC
8	omegapdb_1gs5	CC(=O)N[C@@H](CCC(=O)O)C(=O)O
9	omegacsd_CIBPEI_het	C=C(C)c1ccccc1C(=C)Cc1ccc2ccccc2c1
10	astex_1q1g_het	C=c1cccc2c1=CC=C2[C@@H]1C[C@H](CCC)[C@@H](C)[C@H]1C
11	omegapdb_1zvx	COc1ccc(-c2ccc(S(=O)(=O)N[C@@H](C(C)C)P(=O)(O)O)cc2)cc1
12	omegacsd_FBPAZD_2	O=C(CCCN1CCC2(CC1)C(=O)NCN2c1ccccc1)c1ccc(F)cc1
13	omegapdb_2bgd	COc1ccc(-c2ccccc2)cc1N1CC(=O)NS1(=O)=O
14	astex_1ywr_het	C=C1C(c2ccc(C)cc2)=C(c2cccc(C[C@@H](C)c3ccccc3)c2)C(C)=C1C1CCCCC1
15	astex_1tt1_het	C=C(C)CC1[C@@H](C(=C)C)CC[C@@H]1C(=C)C
16	grayson_bach	C[C@@]12CNC(=O)[C@@](C)(C1)C[C@](C)(c1nc3cc(C(=O)c4ccccc4)ccc3o1)C2
17	grayson_sulphon	O=S(=O)(Nc1ccccc1)c1cccc(S(=O)(=O)Nc2ccccc2)c1
18	grayson_takemoto	CN(C)[C@@H]1CCCC[C@H]1NC(=S)Nc1cc(C(F)(F)F)cc(C(F)(F)F)c1
19	omegacsd_KOFKUL	CC(C)c1nc(NC(=O)OCc2ccccc2)oc1-c1ccccc1
20	omegacsd_CABCUD	Cc1ccc(S(=O)(=O)NC(=O)NN2CCCCCC2)cc1
21	omegacsd_SIHDIW	CC(C)[C@H]1CSC(=S)N1C(=O)[C@H](SC1CCCCC1)[C@H]1CCC(=O)N1C
22	omegacsd_DAFTOT	Cc1ncc(CNC(=O)CCc2c[nH]c3ccccc23)c(N)n1
23	omegacsd_LANXED	C[C@@H](CS(=O)(=O)c1ccccc1)[C@H](c1ccccc1)N1CCCC1
24	omegapdb_2ewb	C[C@H](CS)C(=O)N1C[C@@H](Sc2ccccc2)C[C@H]1C(=O)O
25	grayson_berkessel_2	CN(C)[C@@H]1CCCC[C@H]1NC(=O)Nc1cc(C(F)(F)F)cc(C(F)(F)F)c1
26	radical_wong1	C[C@@H]([CH]N1[C@H](C(=O)c2ccccc2)C(=O)N(C)C1(C)C)Cc1ccccc1
27	omegacsd_DADDUH_het	Cc1ccc(Cc2ccc(Cc3ccc(C)cc3C)cc2)c(C)c1
28	omegapdb_1q91	Cc1cn([C@H]2C[C@H]3O[C@](c4ccccc4)(P(=O)(O)O)OC[C@H]3O2)c(=O)[nH]c1=O
29	grayson_boscalid	O=C(Nc1ccccc1-c1ccc(Cl)cc1)c1cccnc1Cl
30	omegacsd_AOPCHY	CC(=O)Oc1ccc(C(=C2CCCCC2)c2ccc(OC(C)=O)cc2)cc1
31	omegacsd_CETZOQ	COC(=O)C1=C(N2CCCCC2)[C@@H]2CCCCC[C@]12C(=O)OC
32	grayson_mikami	O=S(=O)(N[C@@H](c1ccccc1)[C@@H](NS(=O)(=O)C(F)(F)F)c1ccccc1)C(F)(F)F
33	omegapdb_1h2k	O=C(O)CNC(=O)C(=O)O
34	omegapdb_2bts	CC(C)c1cnc(Nc2ccc(S(N)(=O)=O)cc2)s1
35	astex_1owe	NC(=[NH2+])c1ccc2cc(C(=O)Nc3ccccc3)ccc2c1
36	omegapdb_1sa4	Cn1cncc1[C@@](N)(c1ccc(Cl)cc1)c1ccc2c(c1)c(-c1cccc(Cl)c1)cc(=O)n2C
37	omegacsd_HALOPE	O=C(NCCN1CCC(n2c(=O)[nH]c3cc(Cl)ccc32)CC1)c1ccc(F)cc1
38	omegapdb_1xap	C/C(=C\c1ccc(C(=O)O)cc1)c1ccc2c(c1)C(C)(C)CCC2(C)C
39	radical_baran3	COC[C@H]1O[CH]C[C@H](OC)[C@@H]1OC
40	grayson_sasai	CN(Cc1cc2ccccc2c(-c2c(O)ccc3ccccc23)c1O)c1cccnc1
41	test_17	CC(C)[Si](C)(C)c1cc2ccccc2c2c1OP(=O)(O)Oc1c([Si](C)(C)C(C)C)cc3ccccc3c1-2
42	omegacsd_DIGSIV	C[C@H]1CC(=O)c2c(nn(C)c2COCc2ccccc2)C1
43	omegacsd_LACPAG	C[S@]1(=O)=NC(=O)[C@@](O)(CC(=O)OCc2ccccc2)C1
44	omegacsd_FURSEM01	NS(=O)(=O)c1cc(C(=O)O)c(NCc2ccco2)cc1Cl
45	omegacsd_DEBBER	c1ccc(C(Nc2cccc3cccnc23)Nc2cccc3cccnc23)nc1

46	astex_1ig3	Cc1ncc(C[n+]2csc(CCO)c2C)c(N)n1
47	grayson_nakano	N=C(N)N1Cc2c(-c3ccc(-c4ccccc4)cc3)cc3ccccc3c2-c2c(c(-c3ccc(-c4ccccc4)cc3)cc3ccccc23)C1
48	omegacsd_GIHWUP	CCn1c(-c2ccc(OC)c(OC)c2)c/c(=N\c2c(C)cc(C)cc2C)n(C)c1=O
49	radical_gu_liu1	O=C(O)CC[C](CCC(F)(F)F)c1ccccc1
50	omegacsd_YENLEI	CCOC(=O)c1ccccc1Oc1nc(OC)nc(OC)n1
51	omegapdb_1g6c	Cc1ncsc1CCOP(=O)(O)O
52	grayson_inoue	O=C1N[C@@H](Cc2cnc[nH]2)C(=O)N[C@H]1Cc1ccccc1
53	omegapdb_2f7p	O[C@H]1[C@@H](O)[C@H](S)[C@@H](NCc2ccccc2)[C@H]1O
54	omegapdb_1h1s	NS(=O)(=O)c1ccc(Nc2nc(OCC3CCCCC3)c3nc[nH]c3n2)cc1
55	omegacsd_CIHJAE_2	CCOC(=O)c1c(-c2ccccc2Cl)cc(-c2ccccc2)n1C(=O)OCC
56	omegacsd_FUHLID	CC(C)N(C(=S)/N=c1\snc(Sc2ccccc2)n1C)C(C)C
57	omegacsd_HEPYAC	O=C(Cc1ccc(Cl)c(Cl)c1)N1CCc2occc2[C@@H]1C[N@H+]1CC[C@H](O)C1
58	omegacsd_NADYIA	COc1ccc(N2C(=O)[C@@H](OC)[C@H]2[C@@H]2CCCN2C(=O)OC(C)(C)C)cc1
59	omegacsd_CATXIE	O=C([O-])c1ccccc1C(=O)NCCc1[nH]c2c([nH+]1)CCCC2
60	omegacsd_KINJIA	CCOC(=O)C1=C(C)NC(C)=C(C(=O)OCC)C1c1ccc(-c2ccc(C)cc2)o1
61	omegacsd_LACPAG_noheter	C=C(CCc1ccccc1)C[C@]1(C)CC=C(C)C1
62	omegacsd_MBZTZT10	S=c1[nH]nc(CCN2CCOCC2)n1Cc1ccccc1
63	astex_1mzc	CC[C@@]1(c2cccc(Oc3cc([C@](C)([NH3+])c4cncn4C)ccc3C#N)c2)CCCCN(C)C1=O
64	test_20	CN(C)[C@@H]1CCCC[C@H]1NC(=S)Nc1cc(C(F)(F)F)cc(C(F)(F)F)c1
65	omegacsd_SAVXOC	COC1=C(C(=O)/N=c2\ccccn2C)N(C)S(=O)(=O)c2ccccc21
66	omegapdb_1m5f	Cc1onc(C(=O)O)c1C[C@H](N)C(=O)O
67	omegacsd_FANRER	COc1cc2[nH+]c(N3CCN(C(=O)[C@@H]4COc5ccccc5O4)CC3)nc(N(C)C)c2cc1OC
68	omegapdb_1gz8	CC(C)C(=O)COc1nc(N)nc2nc[nH]c12
69	omegacsd_YIHJAA	CN(C(=O)Cc1ccc(Cl)c(Cl)c1)[C@H](C[NH+]1CCCC1)c1ccccc1
70	omegacsd_FPAMCA	O=C(O)c1ccccc1Nc1cccc(C(F)(F)F)c1
71	omegacsd_DADDUH	Clc1cnc(Oc2ccc(Oc3ncc(Cl)cc3Cl)cc2)c(Cl)c1
72	omegacsd_PMBSAN10	COc1ccc(N(C(C)C)S(=O)(=O)c2ccc(OC)cc2)cc1
73	omegapdb_1rf6	O=C(O)C[NH2+]CP(=O)(O)O
74	omegacsd_BUHNIB_het	CCc1c(C)cc(CCc2ccccc2)cc1[C@H]1CC[C@H](C)CC1
75	astex_1l7f	CCC(CC)[C@H](NC(C)=O)[C@@H]1[C@H](O)[C@@H](C(=O)O)C[C@H]1NC(N)=[NH2+]
76	astex_1g9v	Cc1cc(C)cc(NC(=O)Cc2ccc(OC(C)(C)C(=O)O)cc2)c1
77	omegapdb_1syh	N[C@@H](Cn1c2c(c(=O)[nH]c1=O)CCC2)C(=O)O
78	grayson_ricci	O[C@H]1Cc2ccccc2[C@H]1NC(=S)Nc1cc(C(F)(F)F)cc(C(F)(F)F)c1
79	omegapdb_1wvj	N[C@@H](CC1=CCCCc2onc(O)c21)C(=O)O
80	omegacsd_DERZAB	CNC(=O)N(C)c1nn(Cc2cccc(F)c2)c(=S)s1
81	omegacsd_LEMXIK	C[C@@H]1C[C@H]2CCC[C@@H](OC(=O)Nc3ccc(Cl)cc3)[C@H]2[C@H]1OC(=O)Nc1ccc(Cl)cc1
82	omegacsd_GANFIK	O=C(NCCc1cn(C(=O)C(F)(F)F)c2ccccc12)C(F)(F)F
83	omegacsd_CIBPEI	O=C(O)c1ccccc1C(=O)Nc1ccc2ccccc2c1
84	omegacsd_BUHNIB	CSc1c(Cl)nc(NCc2ccccc2)nc1N1CCN(C)CC1
85	omegacsd_LINLAV	Cc1ccc(C)c(N[C@H](C)c2nc3ccccc3c(=O)n2Cc2ccccc2)c1
86	omegapdb_1k2u	CCS/C(N)=N/c1ccc(C(F)(F)F)cc1
87	omegapdb_1syh_nohetero	C=C(C)[C@@H](C)Cc1c(C)cc(C)c2c1CCC2
88	omegacsd_YULZOU	CN(CCN(C)c1ccccc1S)c1ccccc1S
89	omegacsd_ACTHBZ_het	C=C(C)Cc1ccc(-c2ccc(CC(=C)C)c(CC)c2)cc1
90	grayson_urea_cinchrona	COc1ccc2nccc([C@H](NC(=O)Nc3cc(C(F)(F)F)cc(C(F)(F)F)c3)[C@@H]3C[C@@H]4CC[N@H+]3C[C@@H]4C)c2c1
91	omegacsd_YAYDEH	FC(F)(F)c1ccc(OC2(c3ccccc3)CC[NH2+]CC2)cc1
92	omegacsd_GASPUL_2	Cn1c([C@@](O)(C[C@@](C)(O)c2ccccc2)c2ccccc2)cc2ccccc21

93	grayson_segphos_2	c1ccc(P(c2ccccc2)c2ccc3c(c2-c2c(P(c4ccccc4)c4ccccc4)ccc4c2OCO4)OCO3)cc1
94	omegacsd_ECBHMP	CCOC(=O)c1[nH]c(/C=C2\CCC(=O)N2CC)c(C)c1C
95	omegacsd_SIHDES	CC(C)[C@H]1CSC(=S)N1C(=O)[C@H](C)[C@H]1CCC(=O)N1C
96	omegacsd_CEJTIU_het	C=C(CC(=C)Cc1ccc(C(C)C)cc1)Cc1ccccc1
97	omegacsd_WEBCOV	O[C@@H](C[NH2+]Cc1ccccc1)[C@H]1CCc2cc(F)ccc2O1
98	omegapdb_2f71	CNC(=O)[C@@H]1Cc2ccc(NS(=O)(=O)O)cc2CN1C(C)=O
99	astex_1owe_het	C=C(C)c1ccc2cc(C(=C)Cc3ccccc3)ccc2c1
100	astex_1tt1	C=C(C)[C@H]1C[NH2+][C@H](C(=O)O)[C@H]1CC(=O)O
101	omegacsd_DUXYOK_2	COc1cc2c(cc1OC)S(=O)(=O)N(c1ccc(C)cn1)C(=O)CC2
102	omegacsd_SIZDOU	O=C(N[C@H]1C=C[C@@H](S(=O)(=O)c2ccccc2)C[C@H]1C(=O)c1ccccc1)OCc1ccccc1
103	omegapdb_1dfo	Cc1ncc(COP(=O)(O)O)c(CNCC(=O)O)c1O
104	omegacsd_PMEPEN	CC1(C)S[C@H]2[C@@H](NC(=O)COc3ccccc3)C(=O)N2[C@@H]1C(=O)O
105	grayson_superquat	CC(C)[C@H]1N(C(=O)Cc2ccccc2)C(=O)OC1(C)C
106	omegapdb_1m5b	Cn1nnc(-c2onc(O)c2C[C@H](N)C(=O)O)n1
107	omegapdb_2brc	CCc1cc(-c2[nH]nc(C)c2-c2ccc3c(c2)OCCO3)c(O)cc1O
108	omegapdb_1l7g	CCC(CC)[C@H](NC(C)=O)[C@@H]1[C@H](O)[C@@H](C(=O)O)C[C@H]1NC(=N)N
109	omegacsd_KECDUR	Cc1cc(C)c(C(=O)C[C@H](C)[C@H]2CCN(C)C2=S)c(C)c1
110	omegacsd_GAVMIZ	CC(C)Oc1ccc([C@@H]2CC(=O)N(Nc3ccccc3)C2=O)cc1
111	omegacsd_HAVLUL	O=C(O)CN1C(=O)N(Cc2ccc(Cl)cc2Cl)C(=O)C1(c1ccccc1)c1ccccc1
112	astex_1w2g	Cc1cn([C@H]2C[C@H](O)[C@@H](CO)O2)c(=O)[nH]c1=O
113	B_Me_EE	C/C=C/C=[N+]1\CCC[C@H]1C(O[Si](C)(C)C)(c1cc(C(F)(F)F)cc(C(F)(F)F)c1)c1cc(C(F)(F)F)cc(C(F)(F)F)c1
114	omegacsd_GIHTUM	CN1O[C@H](COc2ccc(Cl)cc2)C[C@@]1(Cn1ccnc1)c1ccc(Cl)cc1
115	omegapdb_2byh	CC(=O)c1ccc(NC(=O)c2cn[nH]c2-c2cc(Cl)c(O)cc2O)cc1
116	omegapdb_1yvx	Cc1ccc(C(=O)N(c2cc(-c3ccccc3)sc2C(=O)O)C(C)C)cc1
117	omegacsd_DIAVER	COc1ccc(Cc2cnc(N)nc2N)cc1OC
118	omegacsd_YUKKUK	Cc1noc(NS(=O)(=O)c2cccc3c(N(C)C)cccc23)c1C
119	grayson_antilla	O=P1(O)Oc2c(c(-c3ccccc3)cc3ccc4ccccc4c23)-c2c(-c3ccccc3)cc3ccc4ccccc4c3c2O1
120	omegapdb_1uy8_2	CCCCn1c(Cc2cccc(OC)c2)nc2c(N)ncnc21
121	omegapdb_1s8j	C/C=C(C)/C=C/C=C(C)/C=C/C1=C(C)CCCC1(C)C
122	test_19	C=CCOC(=O)C1=C(O)C[C@H](c2ccc(Cl)nc2)[C@@H]([N+](=O)[O-])[C@H]1OC
123	omegapdb_1c1u	N=C(N)c1ccc2[nH]c(Cc3nc4ccccc4[nH]3)nc2c1
124	omegacsd_BEXVOP	COc1cc(Cc2cnc(N)nc2N)cc(OC)c1OC
125	omegacsd_TEDBUZ	Cc1c(NC(=O)NC(=O)c2ccccc2)c(=O)n(-c2ccccc2)n1C
126	omegapdb_1hyo	C[P@@](=O)(O)CC(=O)CC(=O)O
127	grayson_yamamoto	C1CN[C@H](C[NH+]2CCCC2)C1
128	grayson_hexynoate	COC(=O)CCC(=O)C#Cc1ccccc1
129	omegapdb_2j47	CC(=O)N[C@H]1c2[nH]c(C(=O)Nc3ccccc3)c[n+]2[C@H](CO)[C@@H](O)[C@@H]1O
130	omegacsd_CIHFAA	CC(=O)NS(=O)(=O)c1ccc(NC(=O)c2ccccc2C(=O)O)cc1
131	astex_1lpz	Cc1cccc2c1cc(C(=O)NCc1cc(Cl)cc(Cl)c1)n2Cc1cccc(C(N)=[NH2+])c1
132	omegacsd_CMAPTX	CN(C)CC/C=C1/c2ccccc2Sc2ccc(Cl)cc21
133	astex_1s3v	COc1cc(N(C)C[C@@H]2CCc3[nH+]c(N)nc(N)c3C2)cc(OC)c1OC
134	grayson_terada	O=P1(O)Oc2c(-c3ccccc3)cc3ccccc3c2-c2c(c(-c3ccccc3)cc3ccccc23)O1
135	omegacsd_MRMPYR	C[C@]1(c2ccccc2)CC(=O)N(CN2CCOCC2)C1=O
136	astex_1yvf_het	C=C(C)/C(=C/c1ccc(Cc2ccccc2)cc1)CC(=C)c1ccccc1
137	omegacsd_PEKCEO	C[C@H](OC(=O)c1ccc(Br)cc1)c1cccc([C@H](C)OC(=O)c2ccc(Br)cc2)n1
138	omegacsd_ABXBPC_2	C=CCc1ccc(OCCC(C)C)c2c(=O)cc(C(=O)[O-])oc12
139	omegacsd_CALLEG	Cc1ncc(C[n+]2csc(CCO)c2C)c(=O)[nH]1
140	omegapdb_1ofd	O=C(O)CCC(=O)C(=O)O
141	omegacsd_CEWFUF	CN(Cc1nc(-c2ccccc2)no1)c1nc(-c2ccccc2)no1

142	omegapdb_2ggd	O=C(O)C1=C[C@@H](OP(=O)(O)O)[C@@H](O)[C@H](O)C1
143	omegacsd_YOWYAK	COC(=O)CSc1c([N+](=O)[O-])ncn1Cc1ccccc1
144	omegacsd_NADZIB	CC(C)(C)OC(=O)N1CCC[C@H]1[C@@H]1[C@H](O)C(=O)N1Cc1ccccc1
145	omegacsd_COKTAX	COc1ccccc1N1CCN(C[C@H](O)C2=COc3ccccc3O2)CC1
146	astex_1jla	CC(C)c1c(Cc2ccccc2)n(COCc2ccccc2)c(=O)[nH]c1=O
147	omegapdb_1nqu	O=c1[nH]c(=O)c2[nH]c(=O)c(=O)n(C[C@H](O)[C@H](O)[C@H](O)CO)c2[nH]1
148	grayson_johnston	c1ccc2nc(N[C@@H]3CCCC[C@H]3Nc3ccc4ccccc4[nH+]3)ccc2c1
149	grayson_gobel_3	C[C@H]1COc2cc3ccc(-c4c(O)cccc4O)cc3cc2-c2cccc3cccc(c23)/C(N)=[NH+]\1

4. The Clustering Approaches

A. Dihedral angles as descriptors

The global minimum structure in the SDF is analyzed to identify potentially rotatable dihedral angles. As covered in the main text, the below steps are followed:

Step1: Get a list of bonds and bond order matrices with and without H atoms
Step2: Identify bonds of interest by eliminations
Step3: Extend the bonds of interest to dihedral angles

For step 2, a bond is eliminated if it meets any of the following criteria:

A. Bonds to monovalent atoms (F, Cl, Br, I, H);
B. Bonds to CX_3 groups (X=F, Cl, Br, I, H);
C. Bonds in small rings and with bond order > 1
   a. 3-membered ring bonds
   b. Selective bonds with bond order > 1: *ie* bonds that belong to any of the categories below are retained:
      i. Bonds with bond order = 1
      ii. C-X bonds with bond order = 1.5; (X = heteroatoms)
      iii. X-X bonds with bond order = 2.0; (X = heteroatoms)
      iv. C=N bonds (not within a ring)
      v. C-C(-Y) bonds with bond order = 1.5 (Y = O, S or N, C-X bond order = 2)

The bonds in the list C.b. above are potentially fixed and their associated dihedrals are unlikely to be rotatable. The list comes from a preliminary study with 9017 sets of dihedrals from 711 molecules in the Hutchison dataset.[9] These dihedrals have a bond order > 1 in the center bond. Box analyses were conducted on the standard deviation (σ) and range (η) parameters for dihedral angle (θ) values across all the conformers for the 9017 sets of dihedrals (Figure S6).

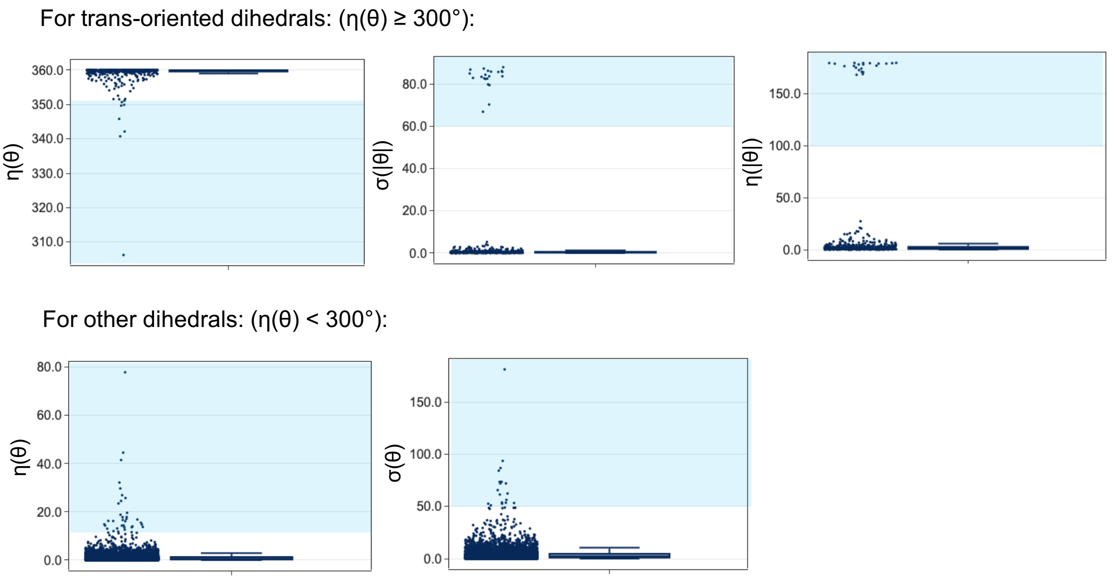

Figure S6. The odd cases: Box plot analyses on standard deviation (σ) and range (η) parameters of dihedral angle values (θ) and absolute dihedral angle values (|θ|). The box plots are based on 9017 sets of dihedral values. Dihedrals with a η(θ) ≥ 300° are identified as trans-oriented dihedrals. The outliers for inspections are in the blue region (*ie* for trans-oriented dihedrals, η(θ) < 350°, σ(|θ|) > 60° and η(|θ|) > 100°; for other dihedrals, η(θ) > 15°, σ(θ) > 50°).

Odd cases from the blue region in Figure S6. are inspected in close examinations and can be categorized into three types. Examples and descriptions are given in Figure S7. Bonds that correspond to these three categories are retained in step 2 (*ie* eliminations of bonds).

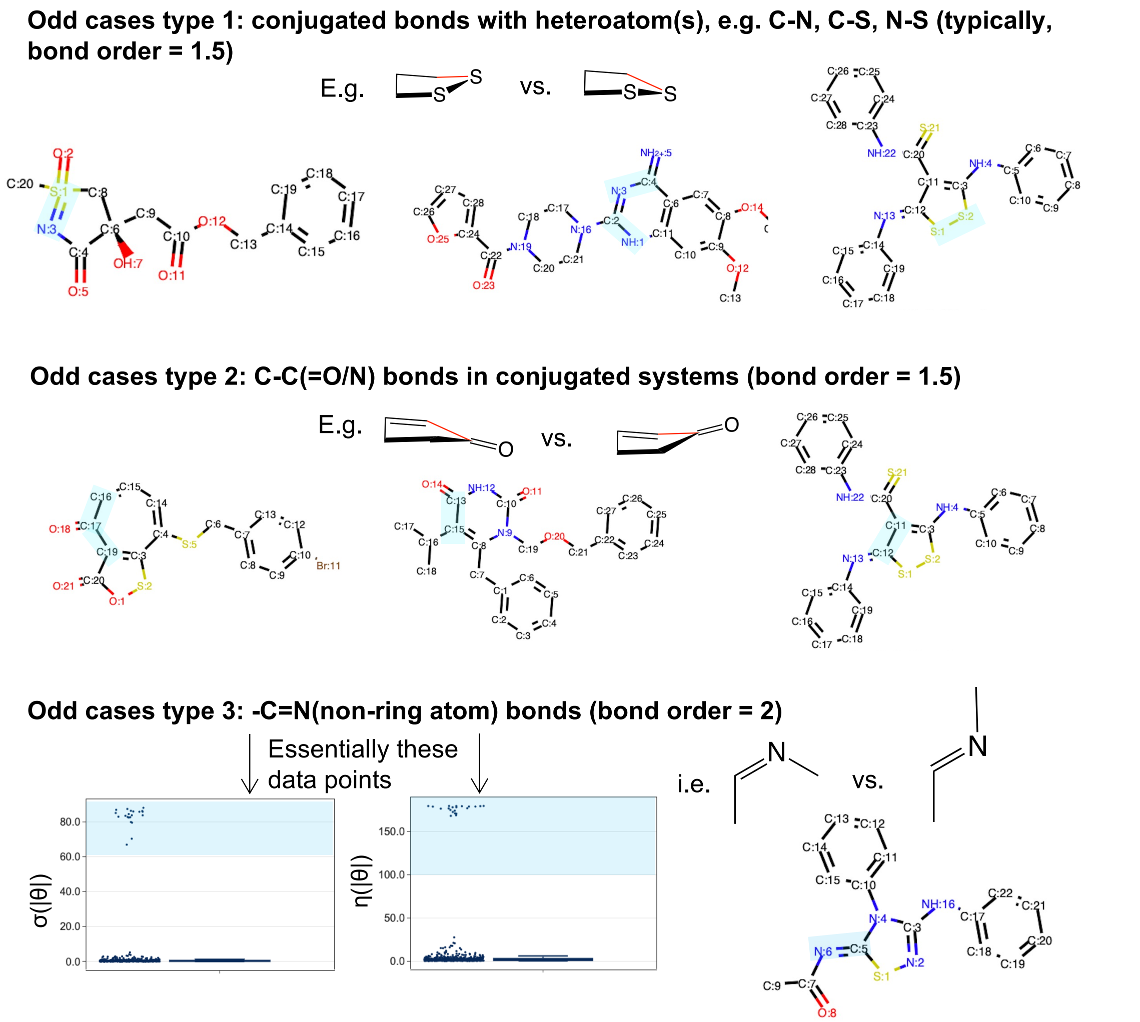

Figure S7. Inspecting the odd cases: examples and descriptions. η(θ) and σ(θ) values (or η(θ) > 350°, σ(|θ|) > 60° and η(|θ|) for trans-oriented dihedrals) for the odd cases are abnormally large or small. These data points are identified as outliers, *ie* beyond the upper or lower fence, in box plot analyses. Examples of molecules that contain these bonds are given. Most type 3 odd cases correspond to data points in the blue region of the σ(|θ|) and η(|θ|) box plots of trans-oriented dihedrals.

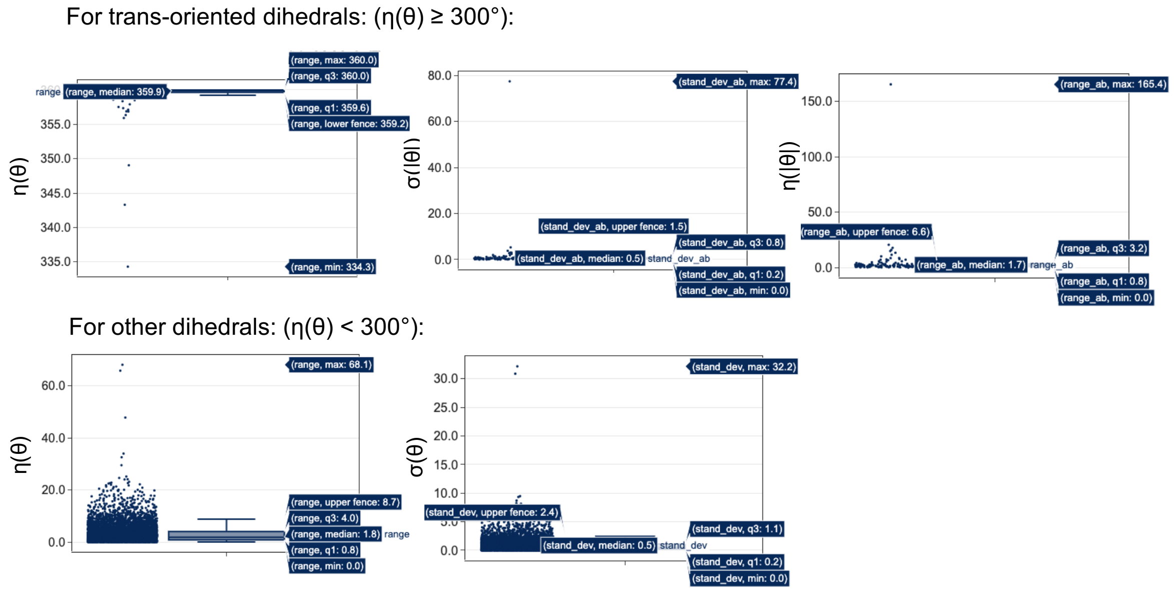

Figure S8. Criteria for identifying fixed bonds: Box plot analyses on standard deviation (σ) and range (η) parameters of dihedral angle values (θ) and absolute dihedral angle values (|θ|). The box plots are based on 7262 sets of dihedral values.

Using the criteria in C. fixed bonds, 7262 out of 9017 dihedrals are identified as having a fixed centre bond and thus are fixed dihedrals. Box plot analyses were conducted on σ and r parameters of θ and |θ| values for the 7262 dihedrals (Figure S8). The results from this set of box plot analyses led to the criteria for identifying fixed dihedrals during the filtering step after dihedral angle calculations (Table 1 in the main text).

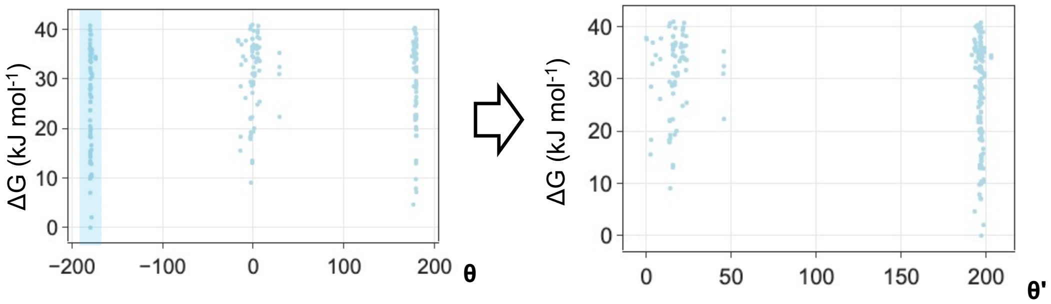

Figure S9. Post-processing on dihedral angle values (θ). The θ values from the negative quadrant are shifted to join up with the data points in the positive quadrant. The axis is then adjusted so that there are no negative values. The process ensures the continuity of θ values within the same cluster. The shifted data points are highlighted in blue.

B. Comparing clustering algorithms

The agglomerative clustering method with the default setting (affinity=Euclidean and linkage=Ward) is chosen for the clustering approach pipeline. Benchmarking tests and hyperparameter tuning calculations have been performed in making this decision.

Several clustering algorithms were benchmarked using the scikit-learn (sklearn) default setting, including K-Means, mini-batch K-means clustering, agglomerative clustering, BIRCH and Gaussian mixture. The clustering algorithms were tested in combination with various priority list generation methods for 150 molecules in the DFT data set. Mean P_{GMT}, Δa_{bins} and overall parameter were calculated. The result is presented in Figure 4.5. The box plot analyses show that altering the clustering algorithms does not significantly change the pipeline's performance.

The time required per molecule, from extracting descriptors to the end of the clustering process, has been calculated (Figure S11). The time required is noticeably different between clustering methods. The clustering approach pipeline using the agglomerative clustering algorithm requires the least computational resources.

Additionally, we have also investigated the DBSCAN and the spectral clustering algorithm. For the DBSCAN method, the esp value is an important hyperparameter. The esp value determines the maximum distance between two samples for them to be accepted as neighbors of each other. The specific range of applicable esp value depends on the molecule because the distributions of dihedral angle descriptors are molecule dependent. Thus, it is difficult to implement the DBSCAN algorithm in the pipeline and retain the level of generality without extensive hyperparameter tuning studies. The spectral clustering algorithm with the default setting failed to return an outcome or gave out user warming during the process for several molecules in the DFT data set. The spectral clustering algorithm requires more computational time than other methods from sklearn (Figure S11). Hence, further hyperparameter tuning tests for debugging are not worthwhile for the spectral clustering model.

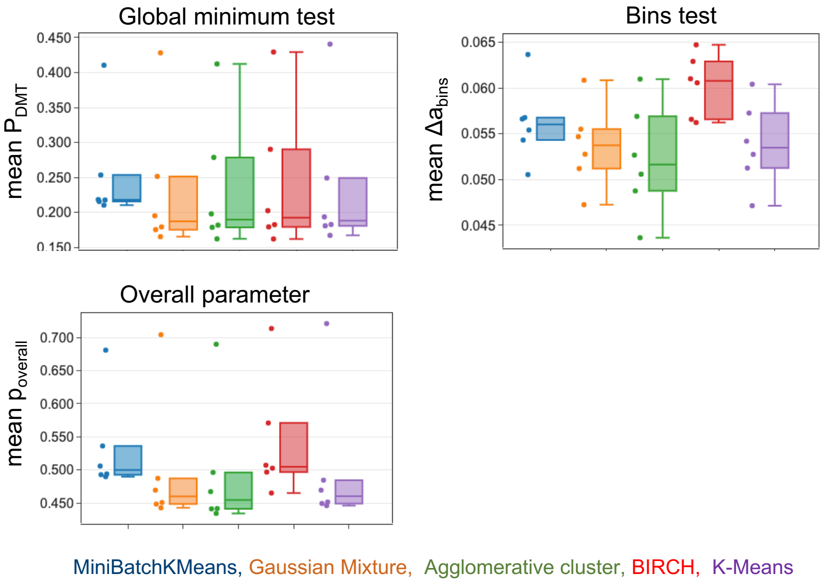

Figure S10. The benchmarking test on clustering algorithms: box plot analyses on performance metrics. The box plots in the graphs are color-coded according to the clustering methods. The clustering algorithms were tested in combination with various priority list generation methods, including *pipeline-x*, *pipeline-ascent*, *pipeline-random* and *pipeline-mix* (Q = 0.15, 0.20 and 0.25; x = 0.8). Hence, there are 6 data points in each box plot.

Method	Total calculation time (150 molecules)	Time per molecule
K-Means	01:59:11	00:00:48
BIRCH	00:07:55	00:00:03
Agglomerative cluster	00:02:18	00:00:01
Spectral Clustering*	04:29:51	00:01:48
MiniBatchKMeans	00:52:51	00:00:21
GaussianMixture	00:42:12	00:00:17

* Underestimated

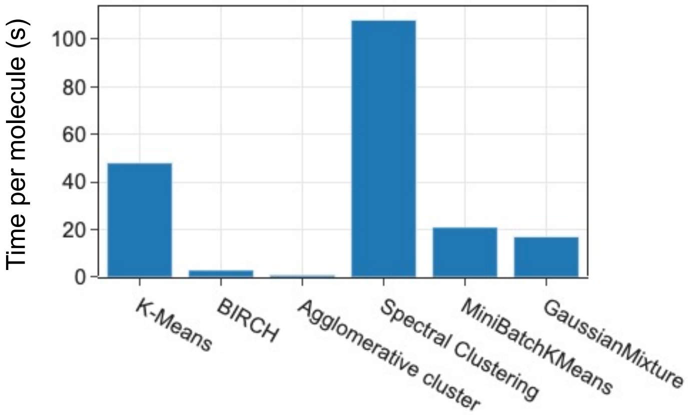

Figure S11. The benchmarking test on clustering algorithms: computational time. The total calculation time and time per molecule (or SDF) are reported above. The values for the spectral clustering method have been underestimated as the algorithm failed to return an outcome for several molecules in the DFT data set.

For the agglomerative clustering method, various linkage and affinity hyperparameter combinations have been tested (Figure S12). The test has been repeated on the clustering approach with two priority list generation methods, *pipeline-x* and *pipeline-ascent*. Mean P_{GMT}, Δa_{bins} and overall parameter were calculated. Altering the hyperparameters does not change the performance metrics significantly.

Linkage (The linkage criterion determines which distance to use between sets of observation**)**

Affinity (Metric used to calculate the linkage**)**

	**Ward**	**average**	**complete**
**Euclidean**	E_W	E_A	E_C
**l1**	NA	l1_A	l1_C
**l2**	NA	l2_A	l2_C
**Manhattan**	NA	M_A	M_C
**cosine**	NA	C_A	C_C

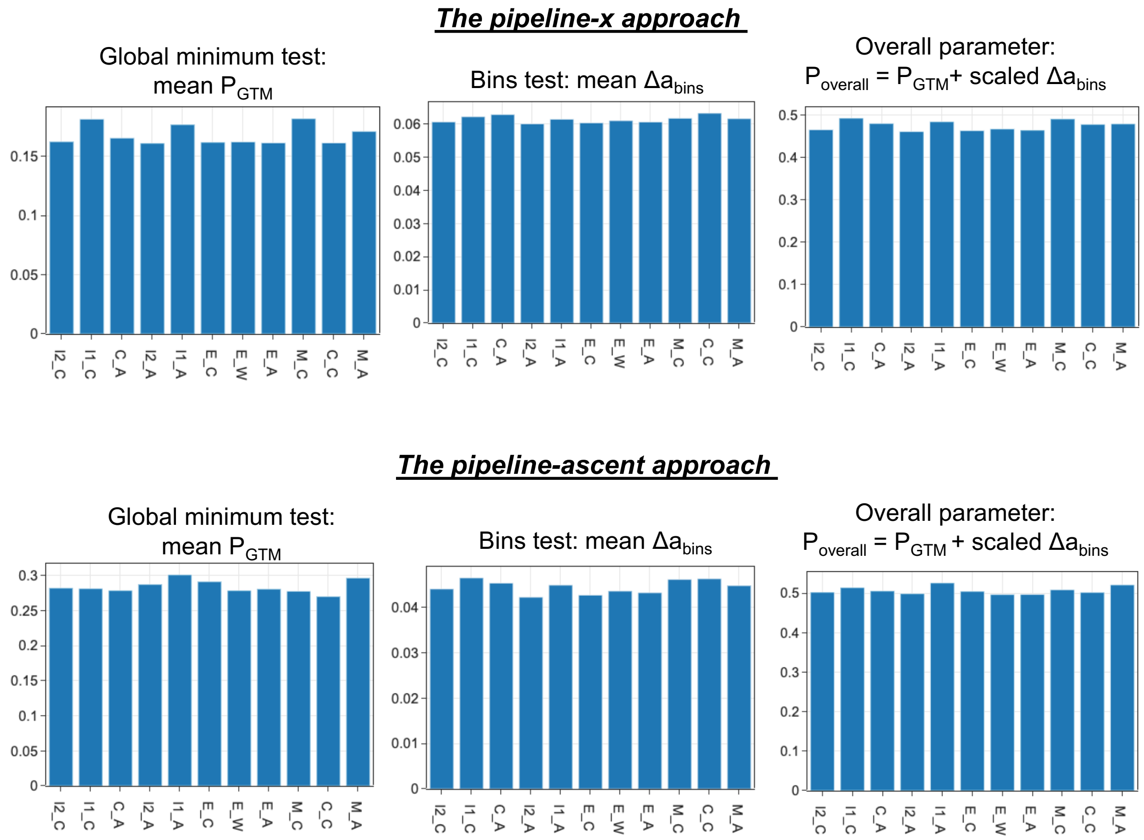

Figure S12. Hyperparameter tuning tests for the agglomerative clustering method. Abbreviations are given for combinations of linkage and affinity settings as shown in the table. The Ward linkage method only works with Euclidean affinity setting

C. Priority list generation methods

A. *Pipeline-ascent*

n_clusters	List of conformer clusters	Priority list
1	$[[E_{FF}^{1}, E_{FF}^{2}, E_{FF}^{3}, E_{FF}^{4}, E_{FF}^{5}, E_{FF}^{6}, E_{FF}^{7}, E_{FF}^{8}]]$	$[E_{FF}^{1}]$
2	$[[E_{FF}^{1}, E_{FF}^{2}, E_{FF}^{3}, E_{FF}^{6}, E_{FF}^{7}, E_{FF}^{8}],[E_{FF}^{5}, E_{FF}^{4}]]$	$[E_{FF}^{1}, E_{FF}^{5}]$
3	$[[E_{FF}^{1}, E_{FF}^{2}], [E_{FF}^{3}, E_{FF}^{4}],[E_{FF}^{5}, E_{FF}^{6}, E_{FF}^{7}, E_{FF}^{8}]]$	$[E_{FF}^{1}, E_{FF}^{5}, E_{FF}^{3}]$
4	$[[E_{FF}^{1}, E_{FF}^{2}],[E_{FF}^{3}], [E_{FF}^{4}],[E_{FF}^{5}, E_{FF}^{6}, E_{FF}^{7}, E_{FF}^{8}]]$	$[E_{FF}^{1}, E_{FF}^{5}, E_{FF}^{3}, E_{FF}^{4}]$
5	$[[E_{FF}^{1}, E_{FF}^{2}, E_{FF}^{5}], [E_{FF}^{3}], [E_{FF}^{4}], [E_{FF}^{6}, E_{FF}^{7}], [E_{FF}^{8}]]$	$[E_{FF}^{1}, E_{FF}^{5}, E_{FF}^{3}, E_{FF}^{4}, E_{FF}^{6}, E_{FF}^{8}]$
6	$[[E_{FF}^{1}, E_{FF}^{2}], [E_{FF}^{3}], [E_{FF}^{4}], [E_{FF}^{5}], [E_{FF}^{6}, E_{FF}^{7}], [E_{FF}^{8}]]$	$[E_{FF}^{1}, E_{FF}^{5}, E_{FF}^{3}, E_{FF}^{4}, E_{FF}^{6}, E_{FF}^{8}]$
7	$[[E_{FF}^{1}, E_{FF}^{2}], [E_{FF}^{3}], [E_{FF}^{4}], [E_{FF}^{5}], [E_{FF}^{6}],[E_{FF}^{7}], [E_{FF}^{8}]]$	$[E_{FF}^{1}, E_{FF}^{5}, E_{FF}^{3}, E_{FF}^{4}, E_{FF}^{6}, E_{FF}^{8}, E_{FF}^{7}]$
8	$[[E_{FF}^{1}], [E_{FF}^{2}], [E_{FF}^{3}], [E_{FF}^{4}], [E_{FF}^{5}] [E_{FF}^{6}], [E_{FF}^{7}], [E_{FF}^{8}]]$	$[E_{FF}^{1}, E_{FF}^{5}, E_{FF}^{3}, E_{FF}^{4}, E_{FF}^{6}, E_{FF}^{8}, E_{FF}^{7}, E_{FF}^{2}]$

B. *Pipeline-random*

n_clusters	List of conformer clusters	List of a randomly chosen conformer within each cluster
1	$[[E_{FF}^{1}, E_{FF}^{2}, E_{FF}^{3}, E_{FF}^{4}, E_{FF}^{5}, E_{FF}^{6}, E_{FF}^{7}, E_{FF}^{8}]]$	$[E_{FF}^{3}]$
2	$[[E_{FF}^{1}, E_{FF}^{2}, E_{FF}^{5}, E_{FF}^{6}, E_{FF}^{7}, E_{FF}^{8}],[E_{FF}^{3}, E_{FF}^{4}]]$	$[E_{FF}^{3}, E_{FF}^{5}]$
3	$[[E_{FF}^{1}, E_{FF}^{2}], [E_{FF}^{3}, E_{FF}^{4}],[E_{FF}^{5}, E_{FF}^{6}, E_{FF}^{7}, E_{FF}^{8}]]$	$[E_{FF}^{3}, E_{FF}^{5}, E_{FF}^{2}, E_{FF}^{4}]$
4	$[[E_{FF}^{1}, E_{FF}^{2}],[E_{FF}^{3}], [E_{FF}^{4}],[E_{FF}^{5}, E_{FF}^{6}, E_{FF}^{7}, E_{FF}^{8}]]$	$[E_{FF}^{3}, E_{FF}^{5}, E_{FF}^{2}, E_{FF}^{4}, E_{FF}^{1}, E_{FF}^{7}]$
5	$[[E_{FF}^{1}, E_{FF}^{2}, E_{FF}^{5}], [E_{FF}^{3}], [E_{FF}^{4}], [E_{FF}^{6}, E_{FF}^{7}], [E_{FF}^{8}]]$	$[E_{FF}^{3}, E_{FF}^{5}, E_{FF}^{2}, E_{FF}^{4}, E_{FF}^{1}, E_{FF}^{7}, E_{FF}^{6}, E_{FF}^{8}]$
6	$[[E_{FF}^{1}, E_{FF}^{2}], [E_{FF}^{3}], [E_{FF}^{4}], [E_{FF}^{5}], [E_{FF}^{6}, E_{FF}^{7}], [E_{FF}^{8}]]$	$[E_{FF}^{3}, E_{FF}^{5}, E_{FF}^{2}, E_{FF}^{4}, E_{FF}^{1}, E_{FF}^{7}, E_{FF}^{6},E_{FF}^{8}]$
7	$[[E_{FF}^{1}, E_{FF}^{2}], [E_{FF}^{3}], [E_{FF}^{4}], [E_{FF}^{5}], [E_{FF}^{6}],[E_{FF}^{7}], [E_{FF}^{8}]]$	$[E_{FF}^{3}, E_{FF}^{5}, E_{FF}^{2}, E_{FF}^{4}, E_{FF}^{1}, E_{FF}^{7}, E_{FF}^{6},E_{FF}^{8}]$
8	$[[E_{FF}^{1}], [E_{FF}^{2}], [E_{FF}^{3}], [E_{FF}^{4}], [E_{FF}^{5}], [E_{FF}^{6}], [E_{FF}^{7}], [E_{FF}^{8}]]$	$[E_{FF}^{3}, E_{FF}^{5}, E_{FF}^{2}, E_{FF}^{4}, E_{FF}^{1}, E_{FF}^{7}, E_{FF}^{6},E_{FF}^{8}]$

Figure S13. Illustrating the *pipeline-ascent* and *pipeline-random* priority list generating method for a molecule with eight conformers: from the clustering result (*ie* lists of conformer clusters) to the final priority list. In the pipeline-ascent method, the lowest energy structures from every cluster are picked into the priority list. In the *pipeline-random* method, conformers are randomly picked from each cluster. The *pipeline-random* approach gives comparable results to the *random approach* in terms of the overall parameter. The blue coloring indicates the most stable conformer from each cluster by FF energy in the list of conformer clusters. The purple coloring indicates a randomly chosen conformer from each cluster. The green coloring indicates a conformer that has not appeared before and is added to the priority list at the corresponding *n_clusters* value.

Tests were performed to determine the best x and Q value for the *pipeline-x* and *pipeline-mix approach*. The default setting (*ie* the setting in Figure S1) was used except for x and Q.

The results are shown in the overall parameter vs x and overall parameter vs Q plot below (Figure S14). Preliminary tests with incomplete DFT data set show that the overall parameter is the lowest when x = 0.8. For the *pipeline-mix approach*, Q = 0.2 gives the lowest overall parameter when x value was kept at 0.8. We repeated the test with the entire DFT data set at a later stage of the project and similar results were obtained.

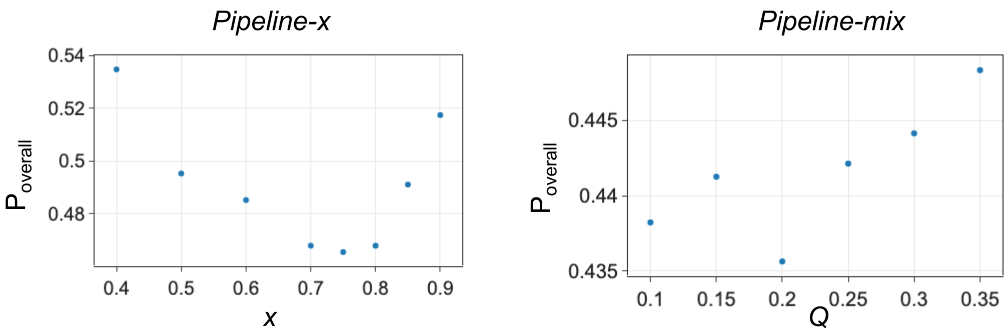

Figure S14. Parameter tuning for the *pipeline-x* and *pipeline-mix approach*: the overall parameter vs x and overall parameter vs Q plot

5. Performance Metrics

A. The Bins Tests

Conformer clusters at the DFT level:

$[[E^3_{FF}], [E^4_{FF}], [E^5_{FF}, E^2_{FF}, E^{10}_{FF}], [E^1_{FF}, E^6_{FF}], [E^8_{FF}], [E^7_{FF}], [E^9_{FF}]]$

The DFT priority list:

$[E^3_{FF}, E^4_{FF}, E^5_{FF}, E^1_{FF}, E^8_{FF}, E^7_{FF}\ E^9_{FF}, E^2_{FF}, E^{10}_{FF}, E^6_{FF}]$

n	Optimized conformers	Conformer clusters at the DFT level	n_{bins}	r_{bins}
10	$[E^3_{FF}, E^4_{FF}, E^5_{FF}, E^1_{FF}, E^8_{FF}, E^7_{FF}$ $E^9_{FF}, E^2_{FF}, E^{10}_{FF}, E^6_{FF}]$	$[[E^3_{FF}], [E^4_{FF}], [E^5_{FF}, E^2_{FF}, E^{10}_{FF}], [E^1_{FF}, E^6_{FF}], [E^8_{FF}], [E^7_{FF}], [E^9_{FF}]]$	7	7/7
8	$[E^3_{FF}, E^4_{FF}, E^5_{FF}, E^1_{FF}, E^8_{FF}, E^7_{FF}$ $E^9_{FF}, E^2_{FF}]$	$[[E^3_{FF}], [E^4_{FF}], [E^5_{FF}, E^2_{FF}], [E^1_{FF}], [E^8_{FF}], [E^7_{FF}], [E^9_{FF}]]$	7	7/7
6	$[E^3_{FF}, E^4_{FF}, E^5_{FF}, E^1_{FF}, E^8_{FF}, E^7_{FF}]$	$[[E^3_{FF}], [E^4_{FF}], [E^5_{FF}], [E^1_{FF}], [E^8_{FF}], [E^7_{FF}]]$	6	6/7
4	$[E^3_{FF}, E^4_{FF}, E^5_{FF}, E^1_{FF},]$	$[[E^3_{FF}], [E^4_{FF}], [E^5_{FF}], [E^1_{FF}]]$	4	4/7
2	$[E^3_{FF}, E^4_{FF}]$	$[[E^3_{FF}], [E^4_{FF}]]$	2	2/7

n_{bins} = number of populated bins in the ideal list for a particular selection of conformers
r_{bins} = n_{bins} / total number of bins in the conformer clusters at DFT level

Figure S15. The derivation of r_{bins} values. r_{bins} is the ratio of populated bins for a particular selection of conformers from partial optimizations. n_{bins} is the number of populated bins in the conformer cluster at the DFT level. The orange coloring indicates duplicate conformers at DFT level.

B. Overall Parameter

The overall parameter ($P_{overall}$, eq. 1) accounts for both the global minimum test (P_{GMT}) and bins test (Δa_{bins}) metrics.

$$P_{overall} = P_{GMT} + (5\ \Delta a_{bins}) \quad (1)$$

The scaling factor for Δa_{bins}, 5, is derived from a preliminary study. Analyses are conducted on Δa_{bins} values of individual molecules for a selection of priority list generation approaches (incl. the *random, every nth, pipeline-x, pipeline-ascent, pipeline-random* and *pipeline-mix approach*). 98.6% of data points in Figure S16B have a value less than 0.2. Thus, a scaling factor of 5 is chosen to give similar weighting to both P_{GMT} and Δa_{bins}.

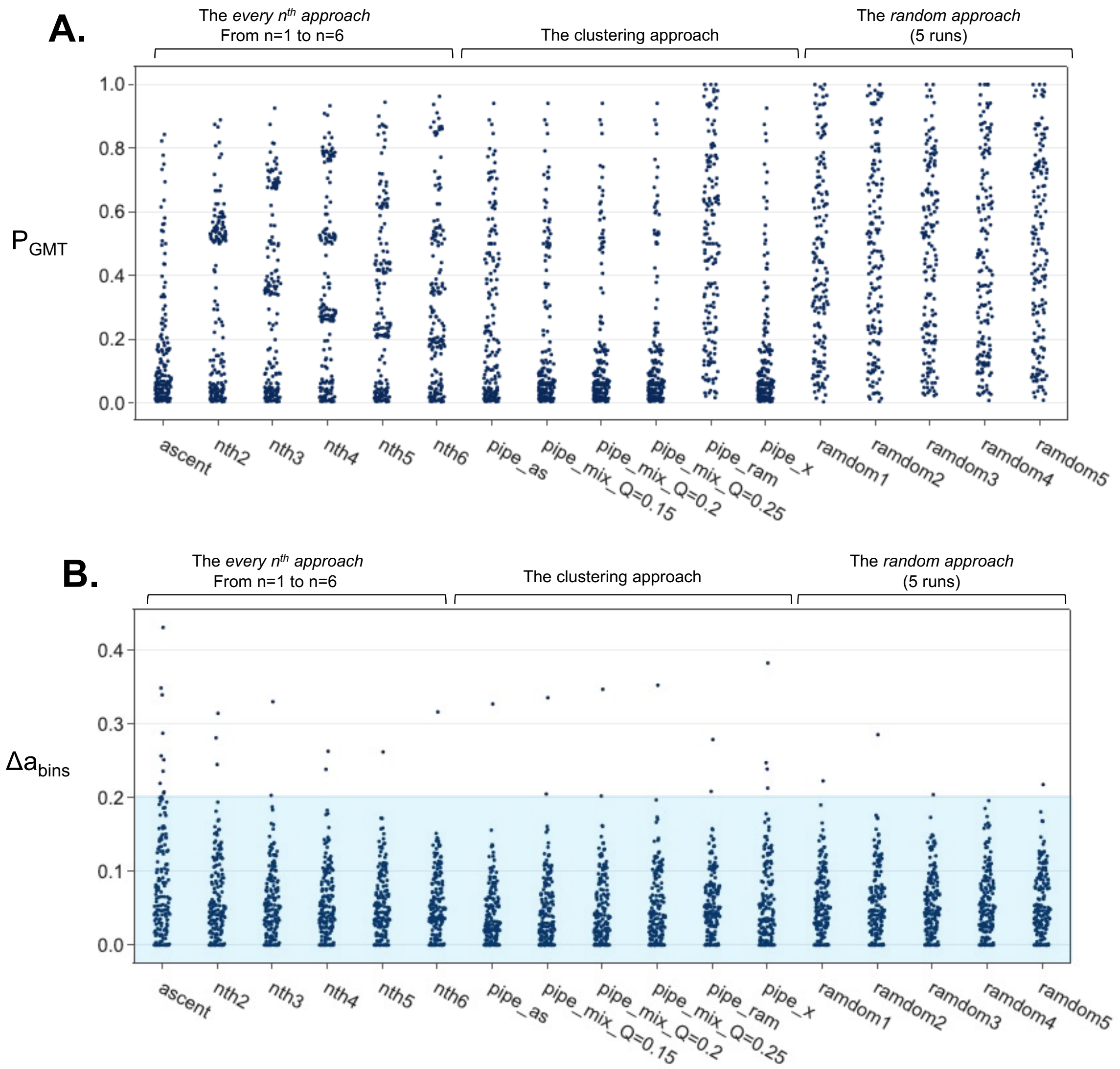

Figure S16. Distributions of A. P_{GMT} and B. Δa_{bins} values for the 150 molecules with different priority list generation approaches. 98.6% of data points have a value less than 0.2 (*ie* the light blue region in plot B).

6. Evaluation

A. Additional plots

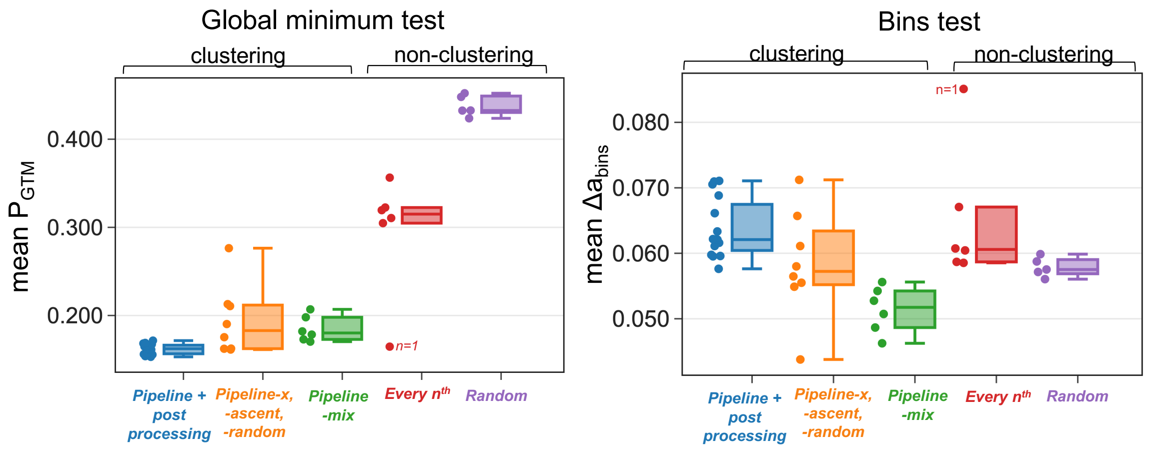

Figure S17. Comparisons between different clustering and non-clustering priority list generation approaches. The global minimum test (P_{GMT}) and bins test (Δa_{bins}) results are presented in the box plots. This figure can be cross-referenced with Figure 10.A in the main text. The tests for the *random approach* have been repeated 5 times, which contributes to the 5 data points in the category. For the *every n^{th} approach*, the tests have been repeated at n = {1, 2, … 6}, which leads to the 6 data points in the category.

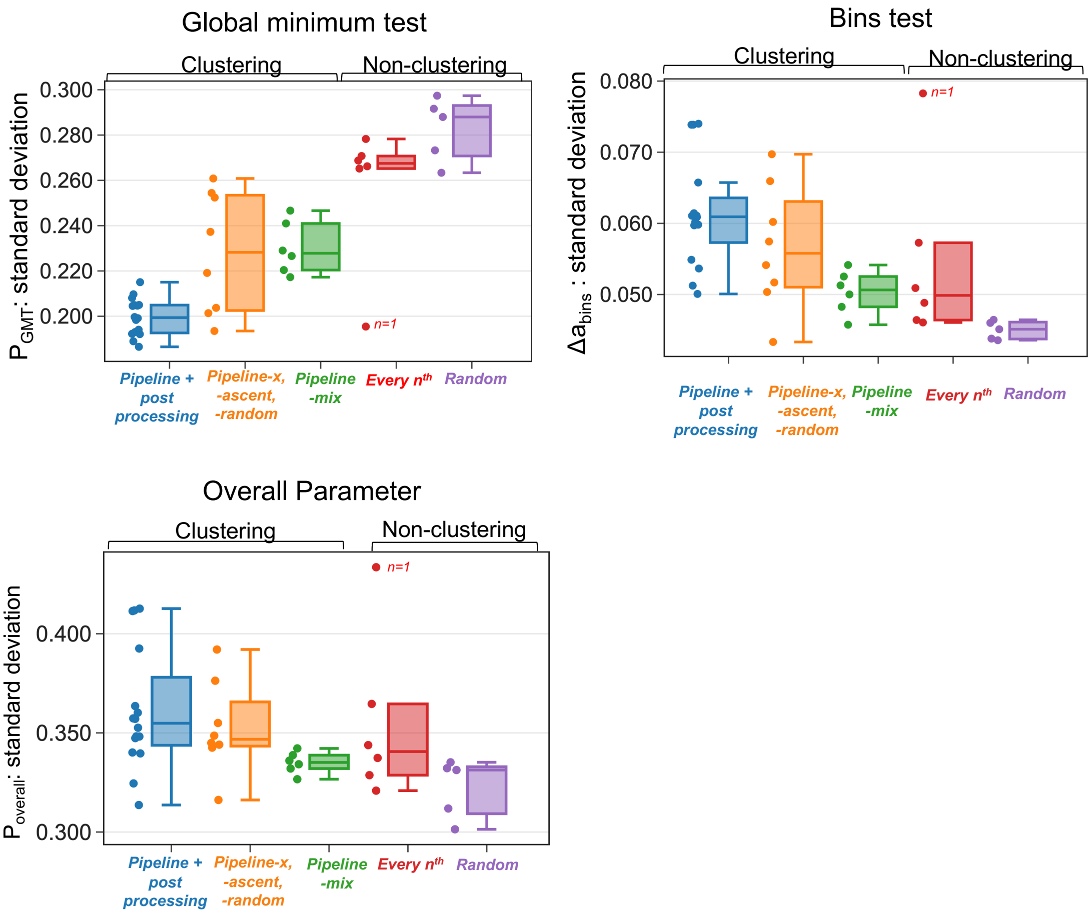

Figure S18. Comparisons between different clustering and non-clustering priority list generation approaches: the standard deviation value for the mean P_{GMT}, Δa_{bins} and $P_{overall}$ in Figure S17. and Figure 10 in the main text.

B. Post-processing procedures

Introducing post-processing procedures only leads to marginal improvements in the global minimum test measurement, mean P_{GMT}. Variations in bins test performance mean Δa_{bins} have been observed, but none of the approaches with post-processing steps outperforms the *pipeline-ascent approach*. Post-processing procedures are not applicable for rigid molecules with less than 3 conformers. The steps involving sequential clustering processes require further developments for radical species if their pseudo structure and actual structure have different atom numbers. For most of the post-processing procedures, only 146 molecules from the DFT data set were considered during evaluation.

Table S2. The performance metrics for the clustering approach *pipeline* + post-processing process approach. The overall parameter ($P_{overall}$), global minimum test measurement (P_{GMT}) and bins test parameter (Δa_{bins}) data are presented below. The data for the *pipeline-x*, *pipeline-ascent* and *ascent approach* are also included for comparisons.

Approach	$P_{overall}$	P_{GMT}	Δa_{bins}
rv5B2_1	0.445	0.157	0.058
rv5B2_5	0.459	0.161	0.060
rv1B3_1	0.461	0.156	0.061
rv1B3_5	0.461	0.156	0.061
rv1B_1	0.464	0.156	0.061
Pipeline-ascent	0.495	0.276	0.044
Pipeline-x	0.468	0.162	0.061
ascent	0.590	0.165	0.085

Table S2 presents the top 5 best-performed clustering approaches with post-processing steps ranked by the overall parameter. Brief descriptions of the post-processing steps in these clustering approach pipelines are given below:

rv5B2_1: The starting point is a priority list generated via the *pipeline-x approach* with $x = 0.8$. When the percentage of re-optimized conformers = 20%, the clustering process is performed with the conformational searching output using the *n_clusters* = x*total number of conformers setting. The percentage of duplicate conformers in the group of re-optimized conformers is deduced via RMSD calculations. If the percentage of duplicate conformers is greater than 20%, x is then set to (1 – %(duplicate conformers)). The $x = 0.8$ setting is used if the percentage of duplicated conformers is less than 20%. ΔG values of the re-optimized conformers are calculated. The list of conformer clusters from the clustering process is sorted according to the clusters-distance array as shown in Figure S19. The new priority list is constructed from the sorted conformer clusters list.

rv5B2_5: The process in rv5B2_1 is repeated for 4 times when the percentage of re-optimized conformers = 20%, 40%, 60% and 80% respectively.

Step 1: get cluster-distance array

The distance to cluster:

1 2 3 4 5 6 ···

Conformer E_{FF}^{1} [74.3, 57.2, 12.6, 422.1, 230.1, 54.9 …]

Conformer E_{FF}^{2} [45.6, 2.2, 102.1, 10.3, 150.3, 78.9 …]

Conformer E_{FF}^{3} […]

…

M x *N* array (M number of clusters x *N* number of conformers)

The re-optimized conformer with the lowest ΔG(DFT): Conformer 2

---

1 2 3 4 5 6 …

Conformer E_{FF}^{2} [45.6, 2.2, 102.1, 10.3, 150.3, 78.9 …]

Step 2

Sort indices of clusters according to the distance value

[2, 4, 1, 6, 3, 5…]

List of conformer clusters

1 2 3 4 5 6

$[[E_{FF}^{1}, E_{FF}^{3}, E_{FF}^{6}], [E_{FF}^{2}, E_{FF}^{5}], [E_{FF}^{7}, E_{FF}^{13}], [E_{FF}^{8}], [E_{FF}^{4}, E_{FF}^{9}], [E_{FF}^{10}] \ldots]$

Step 3

Sort according to [2, 4, 1, 6, 3, 5…]

2 4 1 6 3 5

$[[E_{FF}^{2}, E_{FF}^{5}], [E_{FF}^{8}], [E_{FF}^{1}, E_{FF}^{3}, E_{FF}^{6}], [E_{FF}^{10}], [E_{FF}^{7}, E_{FF}^{13}], [E_{FF}^{4}, E_{FF}^{9}], \ldots]$

Step 4: derive the priority list

$[[E_{FF}^{2}, E_{FF}^{5}], [E_{FF}^{8}], [E_{FF}^{1}, E_{FF}^{3}, E_{FF}^{6}], [E_{FF}^{10}], [E_{FF}^{7}, E_{FF}^{13}], [E_{FF}^{4}, E_{FF}^{9}], \ldots]$

Pick out the duplicate structures

$[E_{FF}^{2}, E_{FF}^{8}, E_{FF}^{1}, E_{FF}^{10}, E_{FF}^{7}, E_{FF}^{4}, \ldots E_{FF}^{5}, E_{FF}^{3}, E_{FF}^{6}, E_{FF}^{13}, E_{FF}^{9}, \ldots]$

Identify the optimized conformers

$[E_{FF}^{2}, E_{FF}^{8}, E_{FF}^{1}, E_{FF}^{10}, E_{FF}^{7}, E_{FF}^{4}, \ldots E_{FF}^{5}, E_{FF}^{3}, E_{FF}^{6}, E_{FF}^{13}, E_{FF}^{9}, \ldots]$

Get the new priority list

$[E_{FF}^{1}, E_{FF}^{2}, E_{FF}^{3}, E_{FF}^{4}, E_{FF}^{8}, E_{FF}^{10}, E_{FF}^{7}, \ldots E_{FF}^{5}, E_{FF}^{6}, E_{FF}^{13}, E_{FF}^{9}, \ldots]$

Re-optimized conformers are sorted using the old priority list

Figure S19. Using the cluster-distance array to sort a list of conformer clusters and derive the new priority list. A 4-step process is followed. The conformer indexes are color-coded to illustrate the sorting process.

rv1B3_1: The starting point is a priority list generated via the *pipeline-x approach* with *x* = 0.8. When the percentage of re-optimized conformers = 20%, a new priority list is generated via the *pipeline-x approach* with the conformational search output SDF. The percentage of duplicate conformers in the

group of re-optimized conformers is deduced via RMSD calculations. If the percentage of duplicated conformers is greater than 20%, x is then set to (1 – %(duplicate conformers)). The x = 0.8 setting is used if the percentage of duplicate conformers is less than 20%. The re-optimization process continues with the new priority list. Conformers that have already been re-optimized are ignored in the new priority list.

rv1B3_5: The process in rv1B3_1 is repeated for 4 times when the percentage of re-optimized conformers = 20%, 40%, 60% and 80% respectively.

rv1B_1: The starting point is a priority list generated via the *pipeline-x approach* with x=0.8. The output from the clustering process for constructing the priority list (*ie* the list of conformer clusters when *n_clusters* = 0.8*total number of conformers) is retained. When the percentage of re-optimized conformers = 20%, ΔG values of the re-optimized conformers are calculated and a new priority list is constructed following the sorting process in Figure S20.

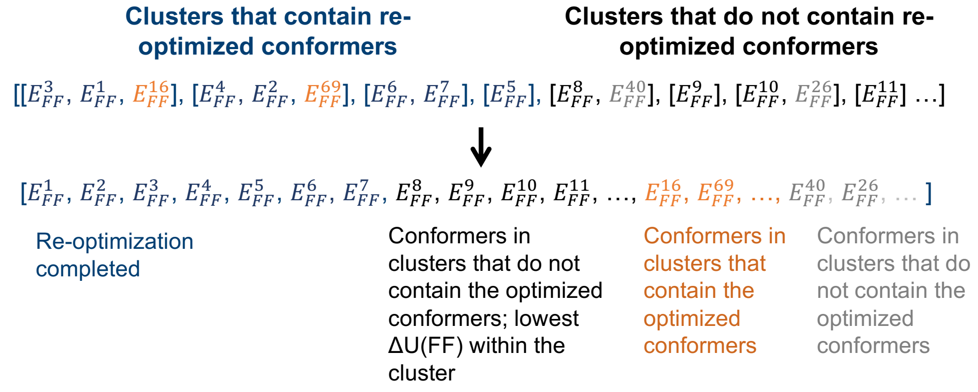

Figure S20. The priority list derivation method in rv1B_1. The conformer indexes are color-coded to illustrate the sorting process.

C. Benchmarking: different levels of theory

The data sets of the 20 Grayson molecules at different levels of theory are gathered or generated to test the generality of the clustering approach pipeline. Please refer to '2. Computational Methodologies B. DFT Calculations' in this document for more details on the calculations. The global minimum test and bins test were performed and the overall parameters were calculated for these DFT data sets (Figure S21).

The 'MMFF → ωB97XD' and 'OPLS3e → M06-2X' data sets produce similar results (*ie* in terms of the overall parameter and mean P_{GMT} vs mean Δa_{bins} plot pattern) as extensive DFT data set of 150 molecules at the ωB97XD/6-311G(d,p)//B3LYP-D3/6-31G(d) level of theory from MMFF structures. The 'MM2 → M06-2X' and 'MMFF → M06-2X' data set shows greater deviations, which may be explained by two reasons. Firstly, the 20 Grayson molecules are more rigid with fewer conformers and more aromatic atoms than other molecules in the DFT data set on average (Figure S22). Secondly, more molecules need to be considered, *ie* a data set of 20 molecules is insufficient to reach convergence. The below procedures were followed to test the hypothesis:

1. Randomly select X% of molecules from the DFT data set (total of 150 molecules) and their priority list for evaluation
2. Perform the tests (the bins test and global minimum test)
3. Repeat the above step 1-2 10 times
4. Calculate the mean and standard deviation for the parameters from the tests

The above procedure has been repeated for X% between 5% to 50%. The result is presented in Figure S23. as the mean and standard deviation of the overall parameter value vs X% plots. At least 30% of the DFT data set or an equivalent of 45 molecules are required to achieve convergence.

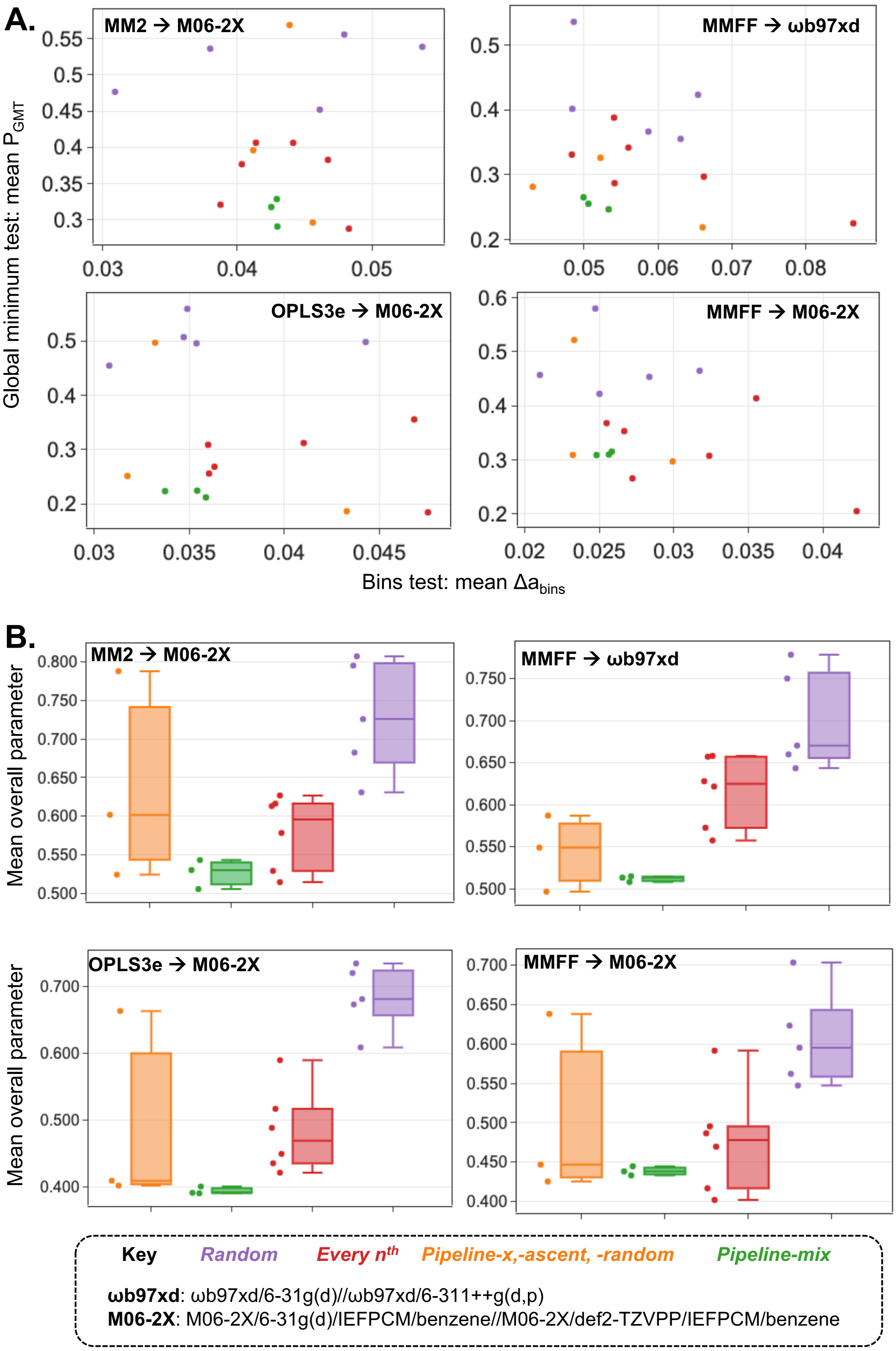

Figure S21. Theory levels benchmarking tests: A. The global minimum test (P_{GMT}) and bins test (Δa_{bins}) results are presented in scatter plots. B. The mean overall parameter data is presented as box plots. The data points on the scatter plots and box plots are colored according to the corresponding priority list generation approach. A reference key is at the bottom of the figure, in which complete descriptions of the theory levels are also given. The tests for the *random approach* have been repeated for 5 times, which contributes to the 5 data points under this category.

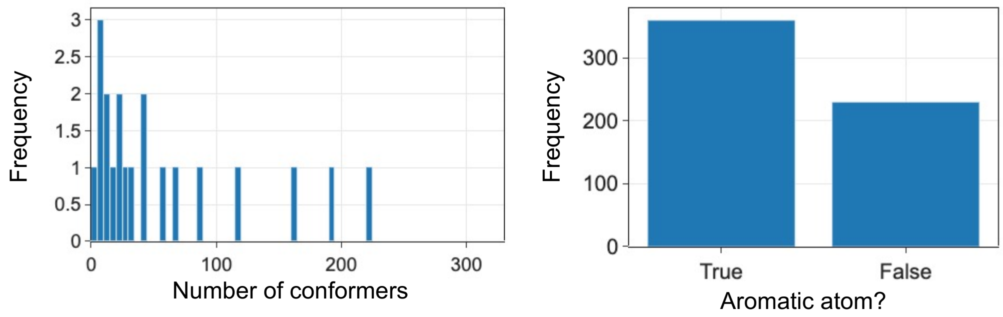

Figure S22. An overview of the 20 Grayson molecules. A histogram of the conformer number is given. The bar chart shows that approximately 2/3 of the non-H atoms in the Grayson molecules are identified as aromatic atoms via RDkit.

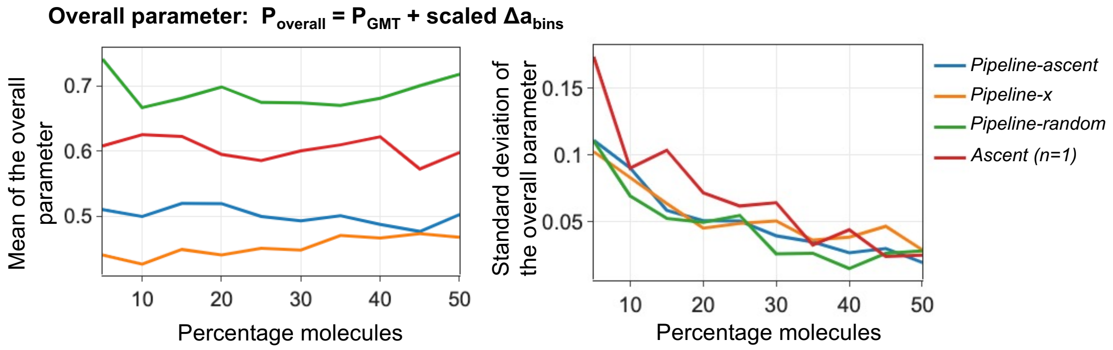

Figure S23. Percentage of molecules required from the DFT data set to reach convergence in the performance parameter. The mean and standard deviation of the overall parameter value vs X% plots are presented.

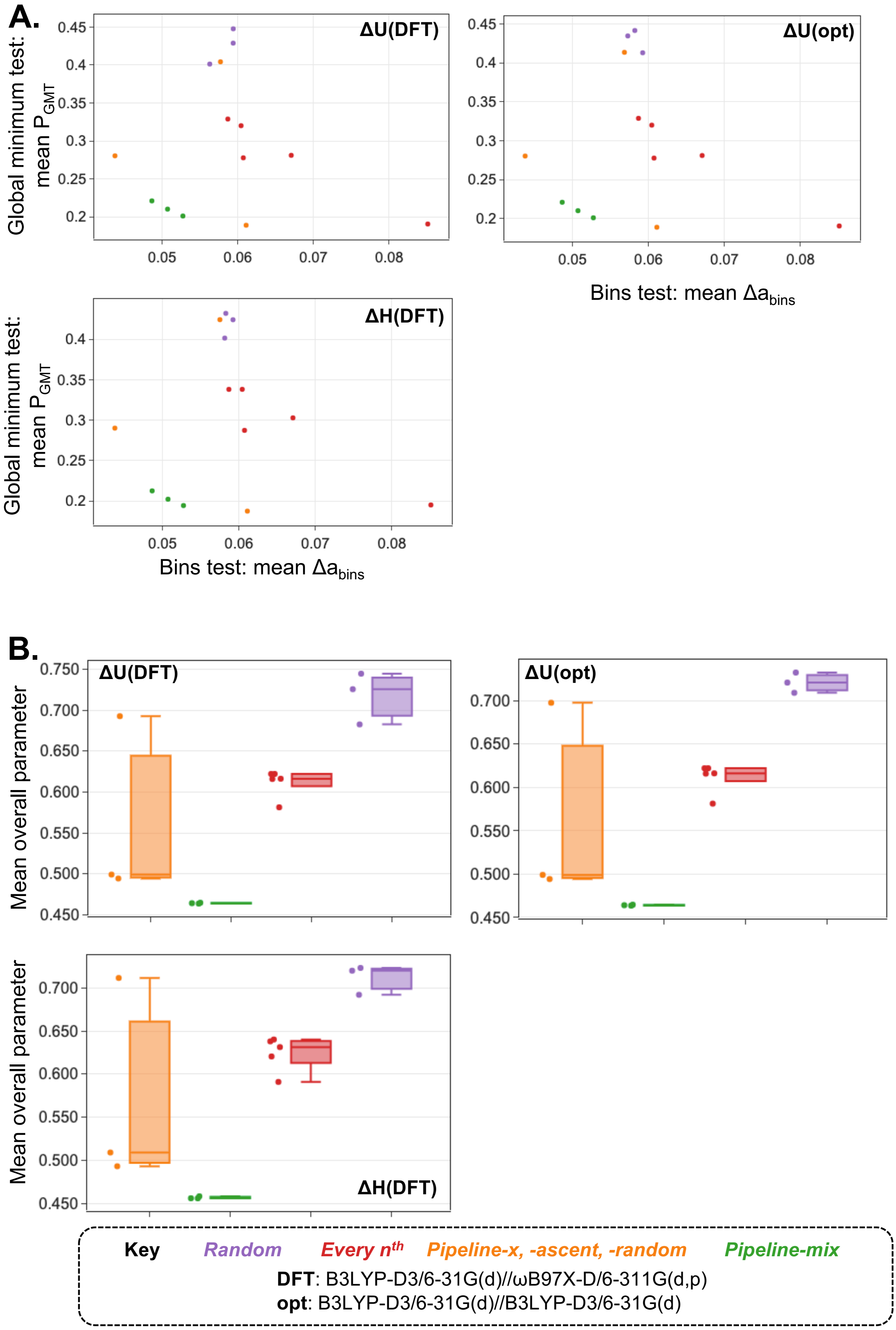

Figure S24. Results with different types of energy values. A. The global minimum test (P_{GMT}) and bins test (Δa_{bins}) results are presented in scatter plots. B. The mean overall parameter data is presented as box plots. The data points on the scatter plots and box plots are colored according to the corresponding priority list generation approach. A reference key is at the bottom of the figure, in which complete descriptions of the theory levels and energy type are also given.

D. Histogram analyses

Duplicate conformers

A DFT conformer is considered a duplicate conformer if its structure already exists in the re-optimized sample during a re-optimization process following a priority list. P_{GMT} values, the number of re-optimized conformers over the total number of conformers, are recorded down when duplicate conformers appear following the priority list from a particular priority list generation approach. Figure S25. presents the histograms of duplicate conformer appearance frequency over the re-optimization process in terms of r_{opt} for the *pipeline-ascent approach*, *pipeline-mix approach*, *ascent approach* and the ideal scenario (*ie* with the DFT lists).

%dup is the number of duplicate conformers over the total number of conformers. %dup for the entire DFT data sets of 150 molecules is 14.9%. %dup can also be calculated for a certain range of P_{GMT}, *ie* in Table S3 and S4.

Table S3: %dup for the *pipeline-ascent* and the *ascent approach* when considering conformers with a r_{opt} less than u in the corresponding duplicate conformer frequency histogram. The percentage difference values are included below.

u	%dup(*pipeline-ascent*)	%dup(*ascent*)	%Difference
0.1	4.12	8.66	52.38
0.2	5.10	10.87	53.09
0.3	5.99	12.27	51.17
0.4	7.36	12.67	41.91
0.5	8.45	12.85	34.29
0.6	9.36	13.19	29.03

Table S4: %dup for the *pipeline-mix* and the *ascent approach* when considering conformers with a r_{opt} less than u in the corresponding duplicate conformer frequency histogram. The setting for the *pipeline-mix approach* is $x = 0.8$ and $Q = 0.2$. The percentage difference values are included below.

u	%dup(*pipeline-mix*)	%dup(*ascent*)	%Difference
0.1	7.49	8.66	13.49
0.2	9.49	10.87	12.65
0.3	8.36	12.27	31.89
0.4	8.33	12.67	34.20
0.5	9.12	12.85	29.04
0.6	9.81	13.19	25.62

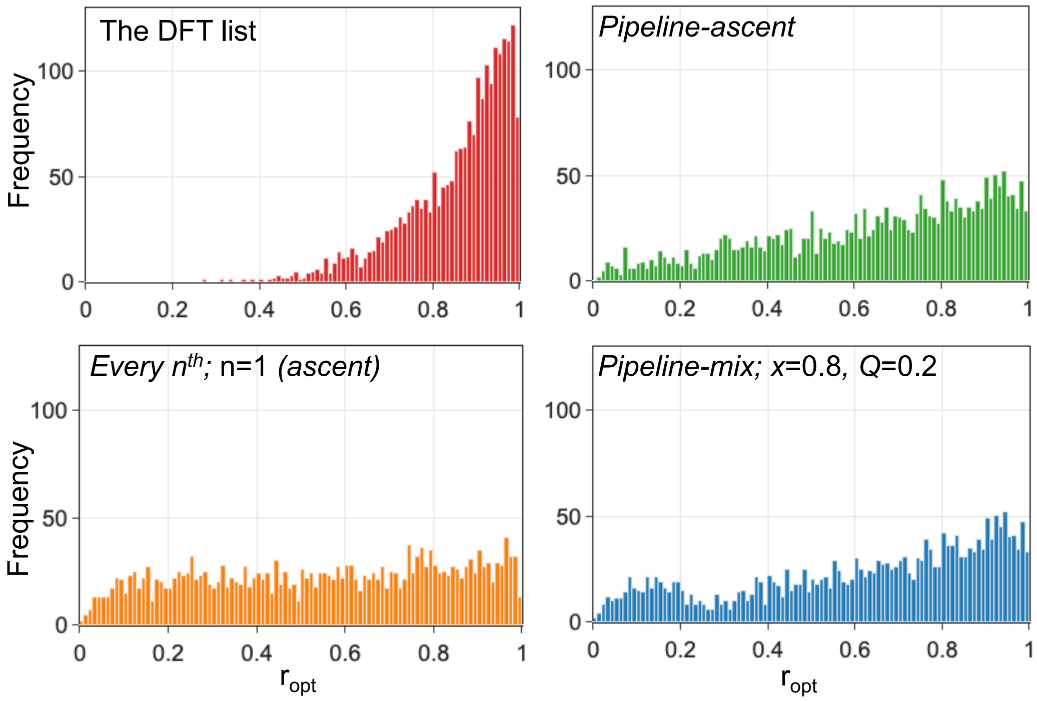

Figure S25. The histograms of duplicate conformers appearance frequency over r_{opt} for the *pipeline-ascent approach*, *pipeline-mix approach*, *ascent* approach and the ideal scenario with the DFT list. The setting for the *pipeline-mix approach* is $x = 0.8$ and $Q = 0.2$.

The above analyses were repeated on a group of molecules from the DFT data set with a sp^3 atom ratio > 0.35. 61/150 molecules satisfy this criterion. For this subgroup, the chance of getting duplicate conformers reduces by up to 59.7% using the priority list from the *pipeline-ascent approach* compared to the *ascent approach* for the first 20% of the re-optimizations (Table S5).

Table S5: Tests on the subgroup of 61 flexible molecules: %dup for the *pipeline-ascent* and the *ascent approach* when considering conformers with a r_{opt} less than u in the corresponding duplicate conformer frequency histogram. The percentage difference values are also included.

u	%dup(*pipeline-ascent*)	%dup(*ascent*)	%Difference
0.1	4.12	8.97	54.10
0.2	4.46	11.09	59.74
0.3	5.31	11.34	53.14
0.4	6.86	12.08	43.24
0.5	8.26	12.04	31.37
0.6	9.10	12.33	26.20

Global minimum parameter distributions

With the default CONFPASS setting (*pipeline-mix*, $x = 0.8$, $Q = 0.2$), the global minimum structure can be obtained in the first 30% of the re-optimizations about 80% of the time on average (Figure S26).

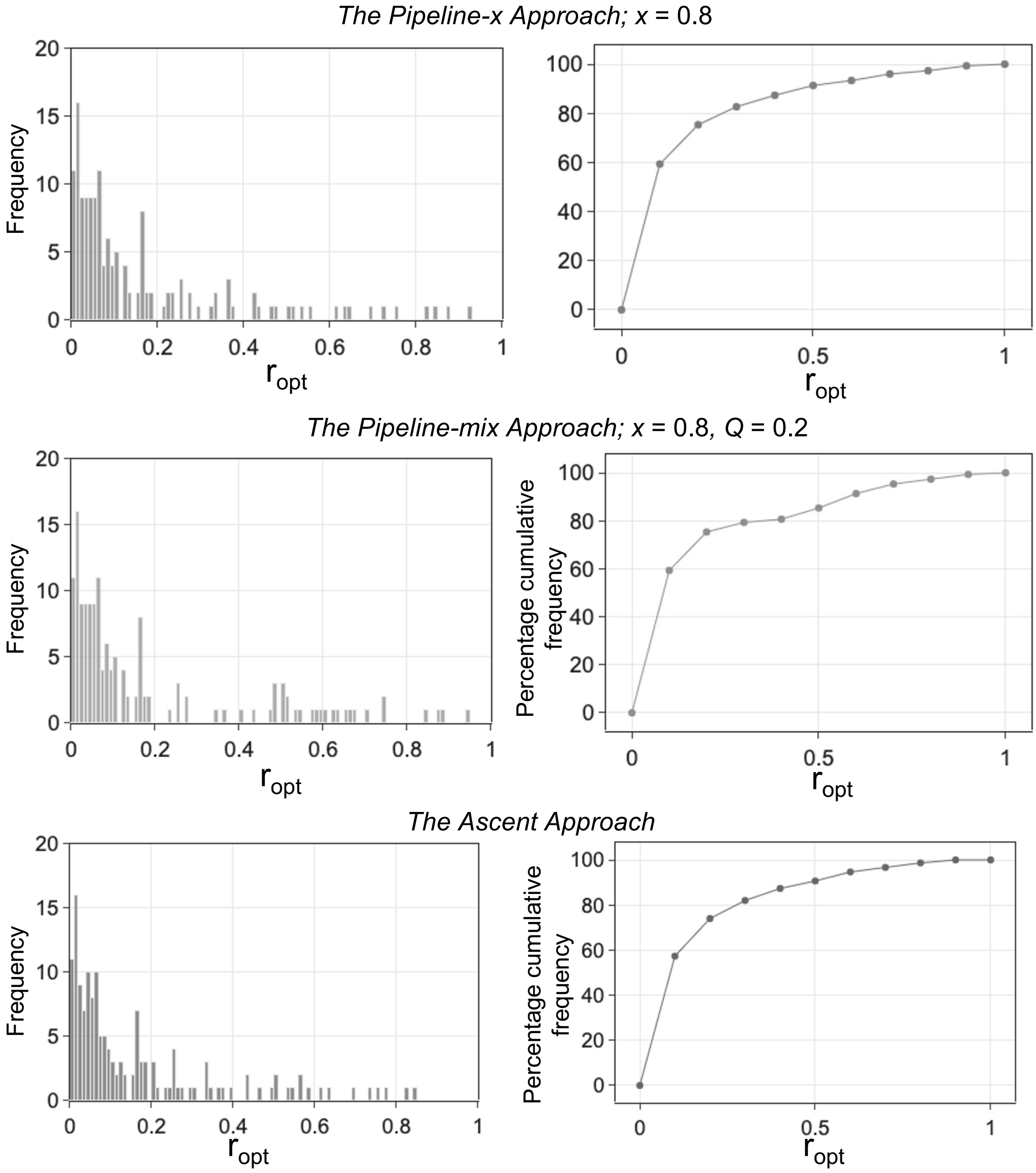

Figure S26. Histogram analyses on the distribution of global minimum test measurement, P_{GMT}, distribution with the *pipeline-x*, *pipeline-mix* and *ascent approach*.

E. Energy cut-off test

Table S6. The effect of changing the energy cut-off in the conformational searching calculations on the CONFPASS workflow

	Total number of conformers	P_{GMT}	Δa_{bins}	r_{opt} when %Conf no longer deviates below 85% using the default LR model
		Energy cut-off = 10 kcal mol^{-1}		
omegacsd_BEXVOP	52	0.60	0.09	0.63
grayson_inoue	59	0.41	0.09	0.44
grayson_gobel	13	0.23	0.00	0.38
		Energy cut-off = 20 kcal mol^{-1}		
omegacsd_BEXVOP	84	0.46	0.09	0.57
grayson_inoue	71	0.49	0.09	0.51
grayson_gobel	52	0.06	0.01	0.27

7. Predict the Completion of the Re-optimization Process

A. Data

1. RMSD calculations

RMSD calculations are performed on optimized structures to identify the duplicate conformers in constructing the χ_{new} vs r_{opt} plots. Previously, in constructing the DFT list, RMSD calculations are performed on every pair of conformers in the conformational searching output file. A slightly different procedure is adopted in the CONFPASS program to speed up the entire process.

Firstly, re-optimized conformers are divided into subgroups. Conformers are sorted in a list according to their ΔG in ascending order. Then, the borders between subgroups are drawn when the difference in ΔG between the neighboring conformer is greater than 0.1 kcal mol^{-1}. RMSD calculations are only performed for conformers within the same subgroup. Tests show that the above procedure gives the same result in identifying the duplicate conformers as following the exhaustive RMSD calculation procedure for 148 molecules in the DFT data set. Slight distinctions are only found for 2 molecules.

2. Input descriptor array

Features of a χ_{new} vs r_{opt} plot were extracted as descriptors:

- Consider the entire χ_{new} data set:
  - Percentage of $\chi_{new} = 0$ (f0)
  - Percentage of $\chi_{new} = 1$ (f1)
  - Percentage of $\chi_{new} \leq 0.1$ (ffrac1)
  - Percentage of $\chi_{new} \leq 0.2$ (ffrac2)
  - Percentage of $\chi_{new} \leq 0.5$ (ffrac5)
- Consider the last 40% of the entire χ_{new} data set:
  - Percentage of $\chi_{new} = 0$ (p0)
  - Percentage of $\chi_{new} = 1$ (p1)
  - Percentage of $\chi_{new} \leq 0.1$ (pfrac1)
  - Percentage of $\chi_{new} \leq 0.2$ (pfrac2)
  - Percentage of $\chi_{new} \leq 0.5$ (pfrac5)
- The highest r_{opt} value at which $\chi_{new} = 1$

A few descriptors were extracted from the conformational searching output of the corresponding molecule:

- No of reoptimized conformers / total number of conformers
- Total number of optimized conformers

B. Model

1. Performance of the local models

We repeated the same training procedure to build a local model with 15 342 data points. The data points come from χ_{new} vs r_{opt} plots from just one set of priority lists, specifically priority lists from the *pipeline-mix* approach ($x = 0.8$ and $Q = 0.2$). The local models did not outperform the global models based on results from the cross-validation tests.

The local support vector classification (SVC) model takes more than 4 hours to train with 12 CPU cores computer. Thus, we did not choose this model for building the global model.

Table S7: Results of the cross-validation tests on ML models for predicting the completion of the DFT re-optimization process with the aim of finding the global minimum structure.

	**Accuracy**	**% False Continue**
RF	88.07%	8.68%
KNN	87.69%	8.32%
GaussianNB	87.75%	4.91%
LR	89.09%	7.63%

2. Hyperparameter tuning tests

Changes in the hyperparameters did not lead to variations in the performance for the global LR model.

Table S8: Hyperparameter tuning cross-validation tests on the global LR model

Max_iter	Random_state	Solver	Penalty	Accuracy	% False Continue
100	0	lbfgs	l2	89.10%	7.60%
200	0	lbfgs	l2	91.44%	2.72%
500	0	lbfgs	l2	88.27%	6.67%
2000	0	lbfgs	l2	88.27%	6.67%
1000	0	lbfgs	l2	89.04%	7.68%
1000	50	lbfgs	l2	89.04%	7.68%
1000	100	lbfgs	l2	89.04%	7.68%
1000	0	newton-cg	l2	89.02%	7.67%
1000	50	newton-cg	l2	89.02%	7.67%
1000	100	newton-cg	l2	89.02%	7.67%
1000	0	saga	l2	89.11%	7.63%
1000	50	saga	l2	89.11%	7.63%
1000	100	saga	l2	89.11%	7.63%

3. Evaluation of the global LR model

Table S9. The performance of the global LR model on descriptors generated based on other priority list generation approaches. The rigorous cross-validation methodology mentioned in the main text was followed (i.e. For each molecule, we took out the relevant data points associated with the molecule, which corresponds to less than 1% of the entire dataset. Models were trained on the filtered dataset.).

Priority list generation method	Input descriptor array parameter	Accuracy	% False Continue
every n^{th}	$n = 1$	89.29%	5.61%
every n^{th}	$n = 2$	74.33%	22.73%
every n^{th}	$n = 3$	74.23%	23.17%
every n^{th}	$n = 4$	71.00%	27.19%
pipeline-x	$x = 0.8$	89.61%	5.19%
pipeline-ascent		83.35%	12.68%
pipeline-mix	$x = 0.8$; $Q = 0.15$	87.43%	7.79%
pipeline-mix	$x = 0.8$; $Q = 0.20$ (default for CONFPASS)	88.27%	6.67%
pipeline-mix	$x = 0.8$; $Q = 0.25$	88.23%	6.62%

C. Percentage Confidence

We performed cross-validation test using the entire dataset (138,078 data points) with the default LR model and recorded down the probability value (p^{LR}) for every prediction. Histograms of p^{LR} for the true predictions, false predictions and the combine are given in Figure S27. Most true predictions have a p^{LR} value close to 1. The percentage of true prediction (%True) values within different p^{LR} ranges were calculated. The mean %True values were computed and plotted against the midpoint of the corresponding p^{LR} range.

The percentage of true predictions can be used as a measurement for confidence, *ie* an equivalent to the percentage confidence (%Conf; mean %True = %Conf; %False = 1 – %True). Using the curve_fit function from scipy.optimize, we fit a sigmoid function on the %Conf vs p^{LR} plot. The sigmoid function can be used to predict the %Conf value given the p^{LR} value of a prediction. For this model, A = 6.29.

$$\%\mathrm{Conf} = \frac{1}{1-e^{-A(p^{LR}-0.5)}} \quad (2)$$

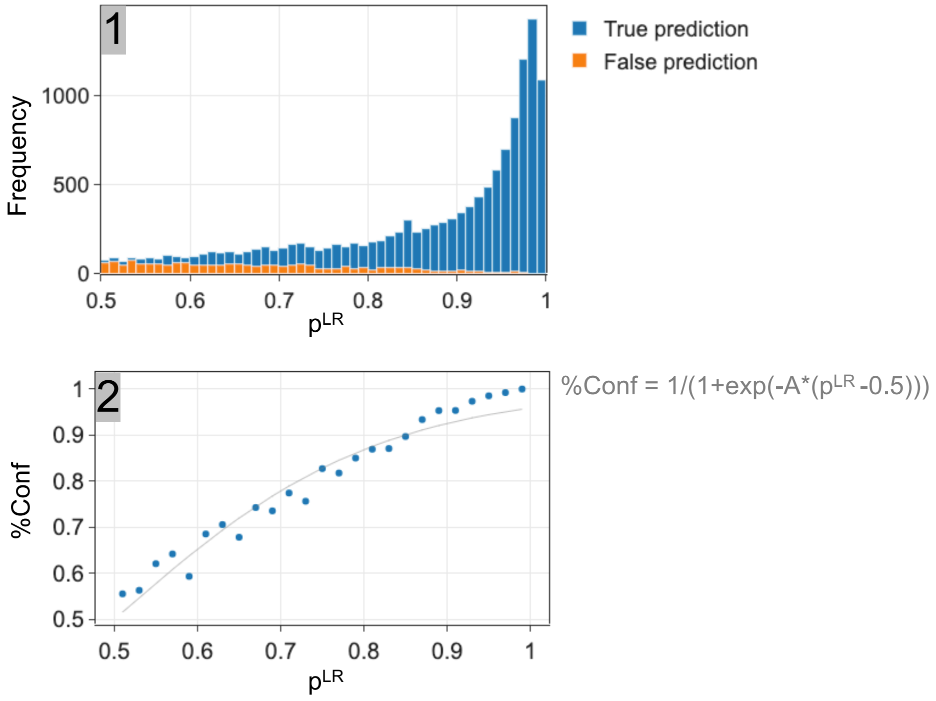

Figure S27. Derivation of the %Conf(p^{LR}) function

D. Evaluation

The breakdown of the results for a molecule (grayson_boscalid) is given below as an example:

Table S10. Predictions on the completion of the re-optimization process for molecule grayson_boscalid with the global LR model following the *pipeline-mix* priority list ($x = 0.8$, $Q = 0.2$). r_{opt} is the number of re-optimized conformers over the total number of conformers of a molecule.

**idx**	$\mathbf{r_{opt}}$	χ_{new}	**Prediction**	**Label**	$\mathbf{p^{LR}}$	**%Conf**
0	0.13	1.00	Continue	Continue	0.78	56.3
1	0.25	1.00	Continue	Stop	0.54	83.7
2	0.38	0.67	Stop	Stop	0.76	88.9
3	0.50	0.50	Stop	Stop	0.83	92.1
4	0.63	0.40	Stop	Stop	0.89	93.7
5	0.75	0.33	Stop	Stop	0.93	94.4
6	0.88	0.29	Stop	Stop	0.95	56.3
7	1.00	0.25	Stop	Stop	0.78	83.7

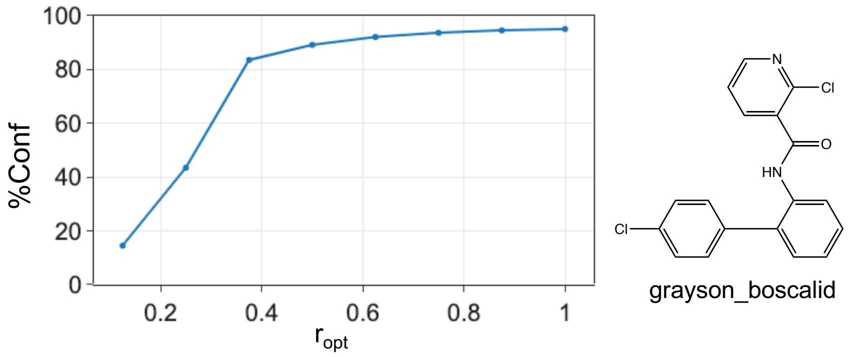

Figure S28. %Conf vs r_{opt} plot for grayson_boscalid

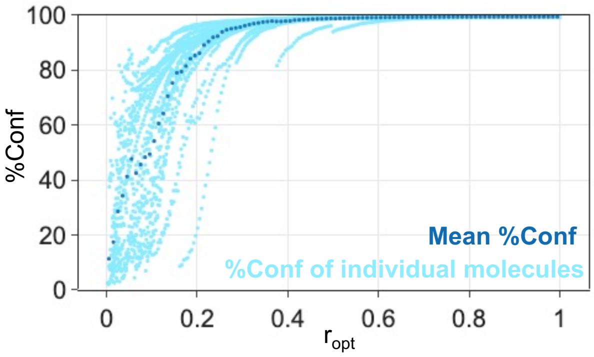

Figure S29. The combined %Conf vs r_{opt} plot and the mean %Conf vs r_{opt} plot for tests with priority lists from the *pipeline-mix* approach (x = 0.8, Q = 0.2) for the flexible molecules with more than 170 conformers. 24/150 molecules in the DFT data set have more than 170 conformers.

E. Boltzmann-weighted property calculations

We further examined the ML models from the perspective of Boltzmann distribution.

$r_{opt,conf=c}$ is the r_{opt} at which %Conf no longer deviates below c in a %Conf vs r_{opt} plot (see Figure S28 for an example). The sum of the Boltzmann distribution population percentage for all the re-optimized conformers with a unique structure (ρ_{total}) was calculated at $r_{opt,conf=c}$.

For c = 60% to c = 90%, We calculate $r_{opt,conf=c}$ and the corresponding ρ_{total} for 150 molecules using the data from the evaluation process. The mean values were calculated, and results are presented in Table S10.

Table S11. Boltzmann-weighted property calculation results: $r_{opt,conf=c}$ and the corresponding ρ_{total} were found for the 150 molecules. The data are from the evaluation of the global LR model. The table present the mean values of the $r_{opt,conf=c}$ and ρ_{total} for c = 60% to c = 90%. The setting for temperature is 298.15 K.

c (%)	Mean $r_{opt,conf=c}$	Mean ρ_{total}
60	0.18	0.67
65	0.20	0.69
70	0.23	0.72
75	0.26	0.74
80	0.30	0.77
85	0.36	0.80
90	0.47	0.86

F. CONFPASS at work

Table S12. CONFPASS at work: applications of CONFPASS in exploring the conformational space of **5** from the Hutchison dataset. Below is the complete version of the data table presented in the main text Figure 16. *v* is the number of re-optimized conformers. **5** has 129 conformers.

v	r_{opt}	Prediction	P^{LR}	%Conf	χ_{new}
2	0.016	Continue	0.52	47.5	0.000
8	0.062	Continue	0.60	34.2	0.268
15	0.116	Continue	0.60	34.5	0.073
21	0.163	Continue	0.58	37.6	1.000
28	0.217	Stop	0.55	57.9	0.000
34	0.264	Stop	0.51	51.4	1.000
41	0.318	Stop	0.64	70.7	0.000
47	0.364	Stop	0.72	79.6	0.000
52	0.403	Stop	0.77	83.8	0.000
53	0.411	Stop	0.78	85.3	0.000
54	0.419	Stop	0.79	85.8	0.000

5

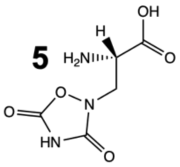

8. Using conformational searching outputs from CREST as the starting point

A. Conformational searching with CREST[10,11]

Conformational searching calculations were performed on a selection of 70 molecules using the default setting (method: GFN2-xTB; conformational searching algorithm: iMTD-GC; energy threshold: 6 kcal mol^{-1}). In the 70 molecules (*ie* the CREST-CS data set; Figure S32), 51 were randomly chosen from the master data set and 19 comes from the work of Grayson *et al.*

We also repeated conformational searching calculations of the 70 molecules with MacroModel for consistent comparisons with the CREST result. The procedure in section 2.4. of this document was followed with the below alternation in the setting. Conformers within an energy window of 25.1 kJ mol^{-1} (i.e an equivalent of 6 kcal mol^{-1}) were saved and redundant structures were eliminated using RMSD at a cut-off of 0.125 Å. On average, more conformer structures were found with CREST compared to MacroModel (Figure S30). The difference in the results may be due to the difference in the MMFF and the GFN2-xTB potential energy surface (PES). For example, semi-empirical methods are better at capturing potential hydrogen bonding interactions than force field methods. Many unique CREST conformer structures feature H bonds that have not been identified with MacroModel as these structures do not correspond to a minimum on the MMFF PES. Secondly, the structural similarity check in the CREST program might not have taken rotations of symmetrical groups, such as methyl groups, into account. For example, conformer 41 and 42 of 'omegacsd_ABHYTZ_het' are visually identical but differ by the rotation of the methyl group (Figure S33).

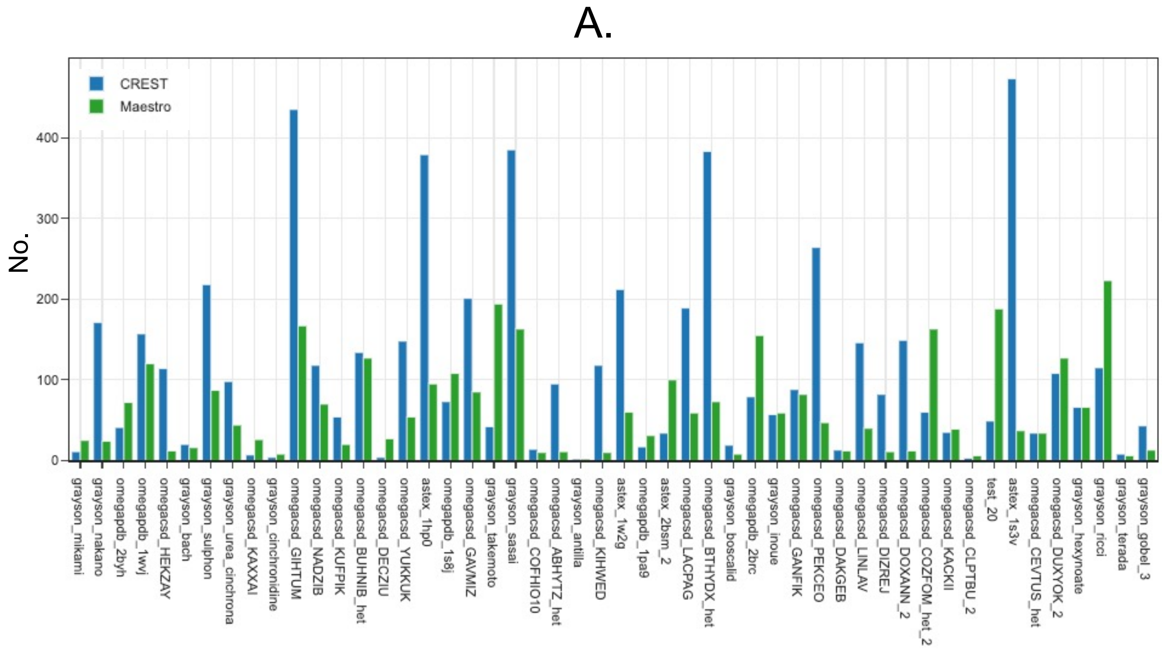

B.

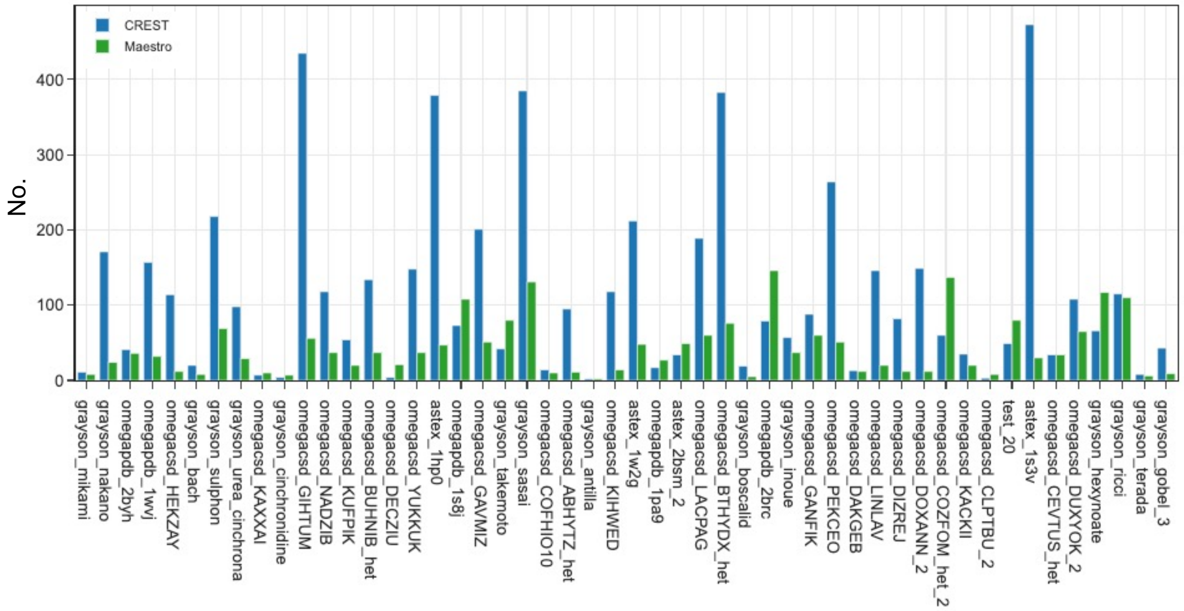

Figure S30. The frequency histogram compares the number of conformers from CREST and MacroModel conformational searches for 50 randomly selected molecules from the CREST-CS data set. A. The setting of conformational searching calculation with MacroModel is the same as the procedures in Section 2.4. B. The setting of conformational searching calculation with MacroModel is aligned with the default setting of CREST.

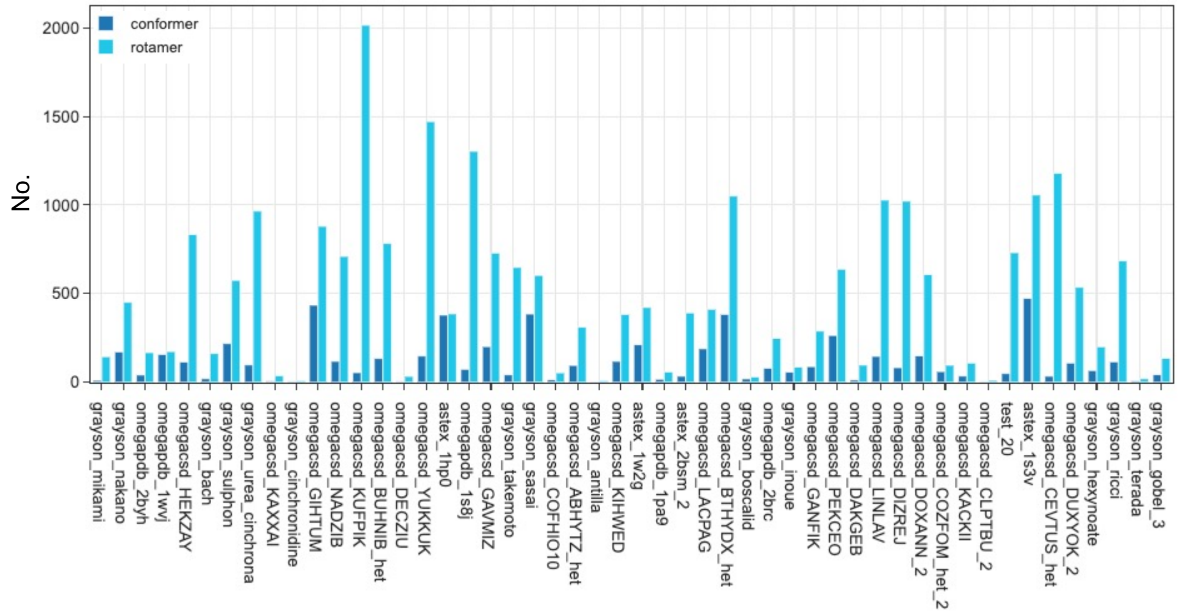

Figure S31. The frequency histogram compares the number of conformers and the number of rotamers identified by CREST conformational searches for 50 randomly selected molecules from the CREST-CS data set.

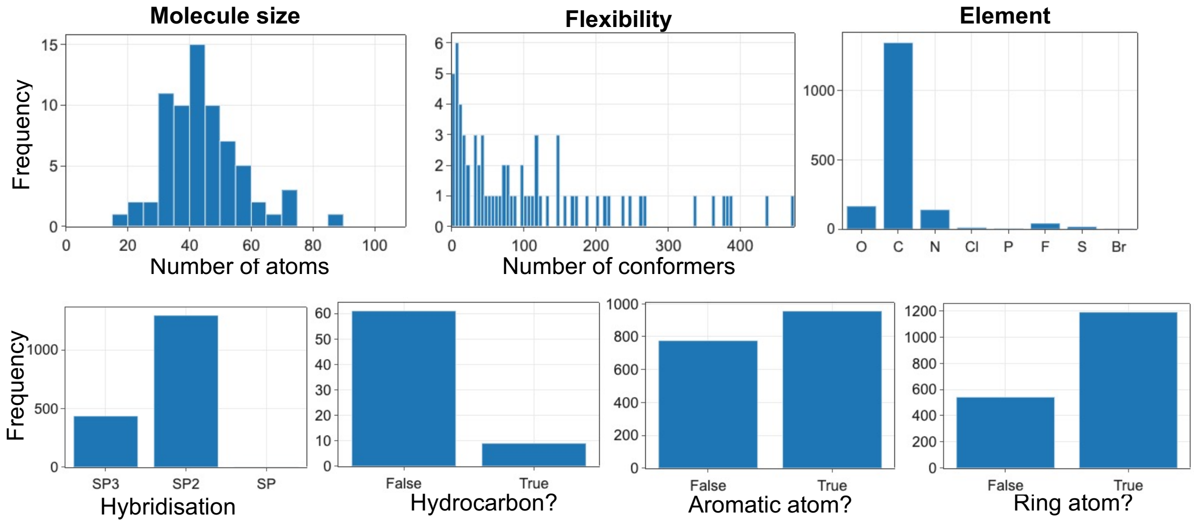

Figure S32. The profile of the 70 CREST-CS data set molecules.

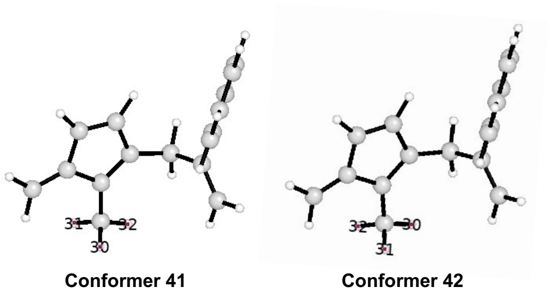

Figure S33. Conformer 41 and 42 of 'omegacsd_ABHYTZ_het': The index of the H atoms in the methyl group is labelled.

B. Re-optimization at the DFT level

We randomly selected 20 molecules from the CREST-CS data set. To evaluate the effectiveness of the CONFPASS pipeline and LR model with our evaluation methods, we followed the procedure in Figure 5 of the main text on the CREST conformational searching results of the 20 molecules. All GFN2-xTB conformer structures from the CREST conformational searches were re-optimized at the DFT level (*ie* the CREST-DFT data set): ωb97xd/6-311g(d,p)//B3LYP-D3/6-31g(d). The conformer clusters at the DFT level and the DFT lists were derived.

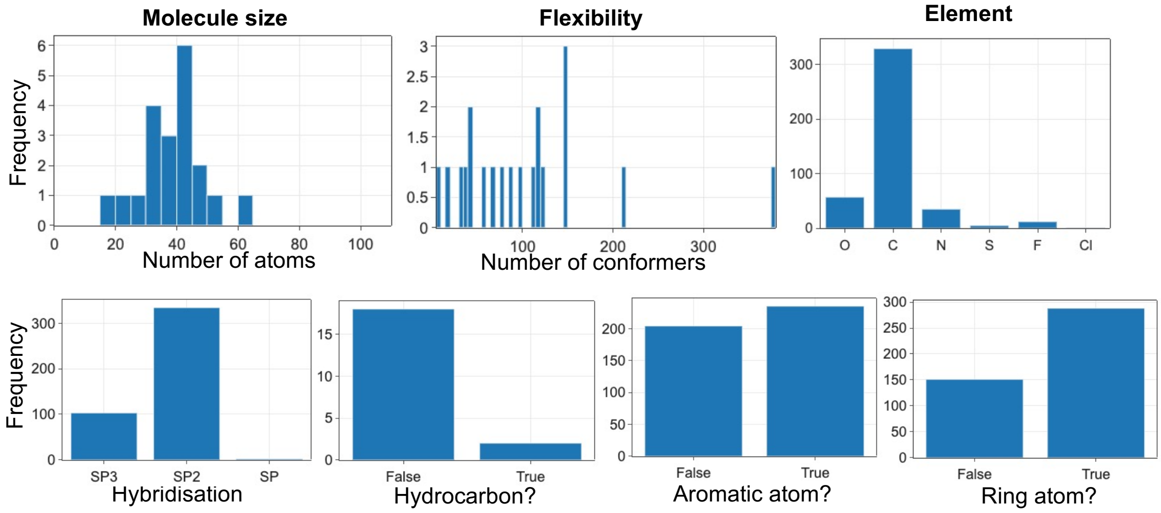

Figure S34. The profile of the 20 chosen CREST data set molecules where DFT re-optimizations were performed on all identified conformers.

C. Performance

All the evaluations below are conducted using Gibbs free energy values with the CREST-DFT data set (GFN2-xTB→ωb97xd/6-311g(d,p)//B3LYP-D3/6-31g(d) optimized conformer structures)

Global minimum test, bins test and overall parameter

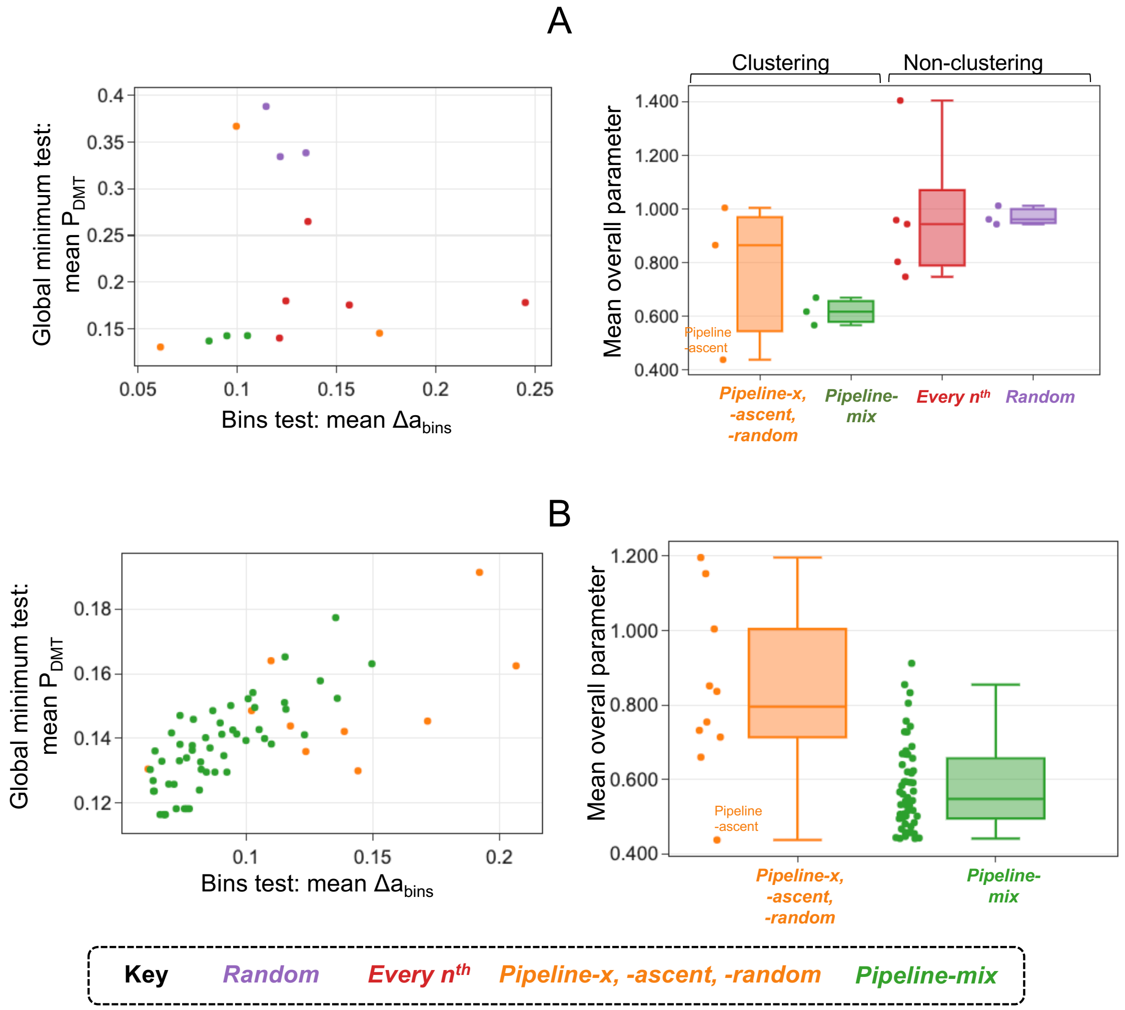

Figure S35. A. Performance of the clustering approach pipeline with CREST conformational searching outputs as the starting point B. Hyperparameter tuning on the x and Q parameter for the *pipeline-x* and *pipeline-mix approach*: The global minimum test (P_{GMT}) and bins test (Δa_{bins}) results are presented in the scatter plot. The mean overall parameter data is presented as the box plot. The data points on the scatter plots and box plots are colored according to the corresponding priority list generation approach. The tests for the *random approach* have been repeated for 5 times, which contributes to the 5 data points under this category.

Hyperparameter tuning tests were conducted on the x and Q parameter for the *pipeline-x* and *pipeline-mix approach*. The *pipeline-mix approach* with $x = 0.2$ and $Q = 0.15$ gives the best (lowest) overall parameter value although this is only marginally better than the *pipeline-ascent approach*.

Duplicate conformers

The works in section 6.D. were repeated with the CREST-DFT data set.

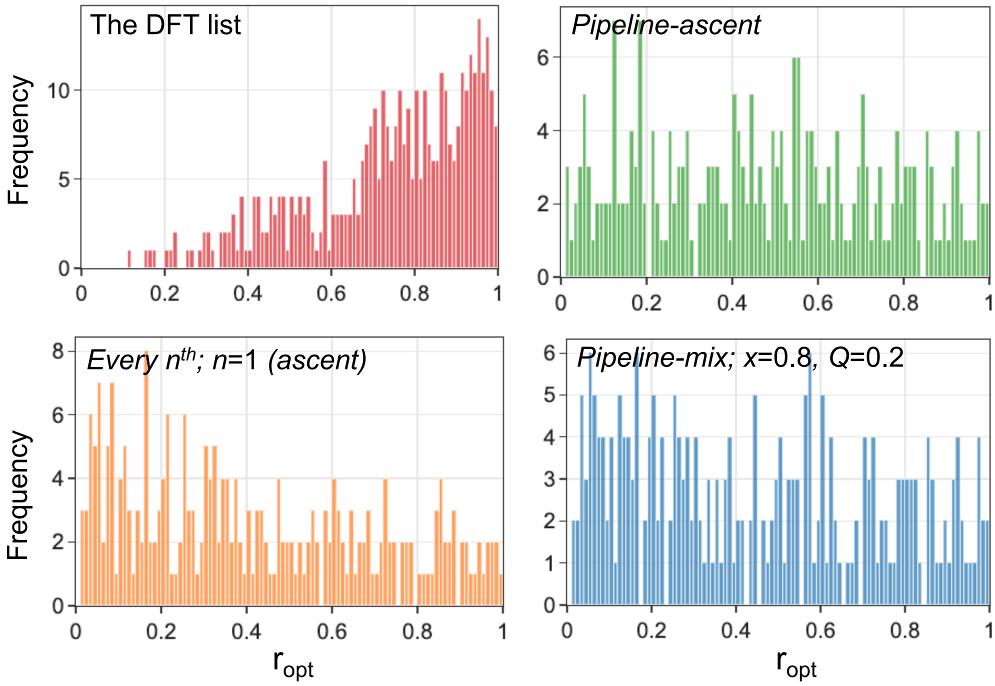

Figure S36. The histograms of duplicate conformers appearance frequency over r_{opt} for the *pipeline-ascent approach*, *pipeline-mix approach*, *ascent approach* and the ideal scenario with the DFT list. The setting for the *pipeline-mix approach* is $x = 0.8$ and $Q = 0.2$.

Evaluation on the ML model for predicting the completion of the re-optimization process

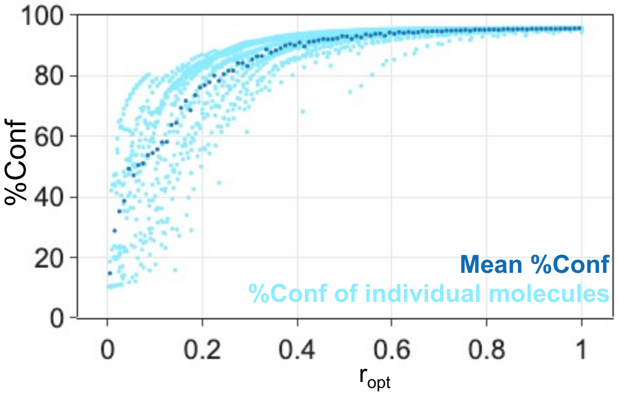

Figure S37. The percentage confidence (%Conf) vs r_{opt} plots for the 20 molecules in the CREST DFT dataset. The predictions and %Conf values come from the global LR model in the CONFPASS package. These data points were separated into 100 bins of equal size according to the r_{opt} values. The mean %Conf was found for each bin. The result has been included in the above plot as dark blue points. The priority list generating method is indicated on the plot.

Boltzmann-weighted property calculations

The same procedure in section 7.F. was followed.

Table S13. Boltzmann-weighted property calculation results: $r_{opt,conf=c}$ and the corresponding ρ_{total} were found for the 150 molecules. The data are from the evaluation of the global LR model. The table present the mean values of the $r_{opt,conf=c}$ and ρ_{total} for c = 60% to c = 90%. The setting for temperature is 298.15 K.

c (%)	Mean $r_{opt,conf=c}$	Mean ρ_{total}
60	0.14	0.61
65	0.17	0.67
70	0.21	0.72
75	0.24	0.76
80	0.28	0.79
85	0.33	0.84
90	0.43	0.90

D. MacroModel vs CREST

11 of the 20 CREST-DFT data set molecules overlap with the DFT data set, which allows us to compare the performance between CREST and MacroModel as the starting point of DFT re-optimization processes. The calculation time of conformational searches with MacroModel and CREST are compared in Figure S38. Both sets of calculations were conducted with computer clusters on 12-core Intel Xeon X5650 (2.67GHz, Westmere) CPUs. Generations of priority lists with CONFPASS were conducted with a regular laptop computer (Macbook Pro with 2 GHz Quad-Core Intel Core i5). It took 3.7 and 3.5 seconds to process and generate priority lists from the 11 SDFs from MacroModel and 11 SDFs from CREST, respectively, with CONFPASS.

The setting for conformational searches with MacroModel: Section 2.A.
The setting for conformational searches with CREST: Section 8.A.

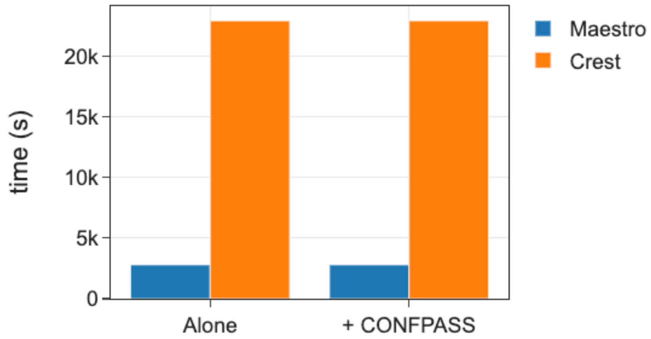

Figure S38. Comparing the calculation time: The sum of the conformational searching calculation time in second of the 11 molecules is presented in the bar chart for MacroModel and CREST.

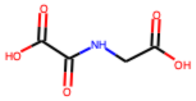

omegapdb_1h2k

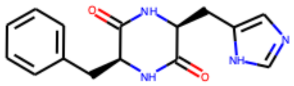

grayson_inoue

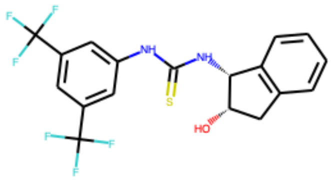

grayson_ricci

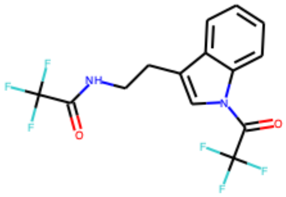

omegacsd_GANFIK

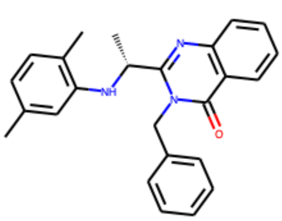

omegacsd_LINLAV

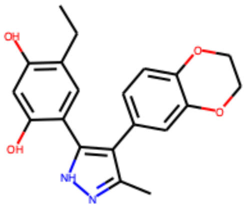

omegapdb_2brc

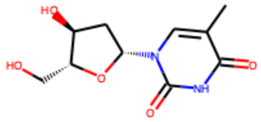

astex_1w2g

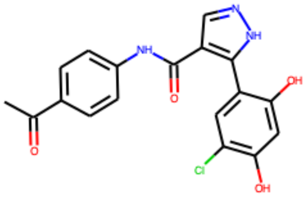

omegapdb_2byh

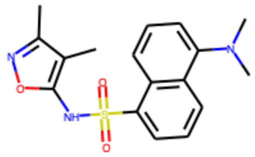

omegacsd_YUKKUK

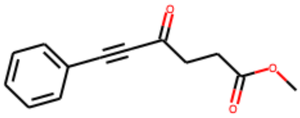

grayson_hexynoate

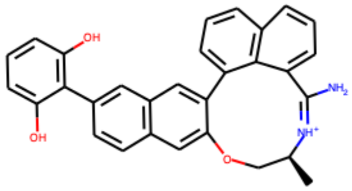

grayson_gobel

Figure S39. Performance comparisons between CREST and MacroModel: the structure of the 11 testing molecules

We have considered situations with and without the use of CONFPASS from the perspective of ΔG of the most stable conformer and Boltzmann distribution population. In most of the cases, similar results were obtained. We also compared the time required for re-optimizations at the DFT level for conformers from CREST and MacroModel (Table S13 and S14).

Table S14. Performance comparisons between CREST and MacroModel (PART 1: performance without CONFPASS, *ie* all re-optimized conformers at the DFT level): DFT structures re-optimized from MacroModel and Crest outputs were considered together when making the below calculations. The sum of the Boltzmann distribution population percentage for all the re-optimized conformers with a unique structure (ρ_{total}) was calculated at r_{opt}=1 considering all re-optimized structures from MacroModel and CREST conformational searches. A low ρ_{total} implies that key low energy structures found with the other conformational searching method have been missed out with the corresponding searching method. ΔG values of the most stable re-optimized conformer compared to the entire set of DFT structures (*ie* all re-optimized structures from MacroModel and CREST) (ΔG_{gl}) are given. In most cases, CREST and MacroModel lead to the same global minimum structure at the DFT level after re-optimization (*ie* ΔG_{gl} = 0 kcal mol^{-1}). The scaled CUP time values required for re-optimizing all conformers from the conformational searching output are given below.

	ρ_{total} at r_{opt}=1		ΔG_{gl} (kcal mol^{-1})		Scaled CPU time	
Molecule Name	MacroModel	CREST	MacroModel	CREST	MacroModel	CREST
omegapdb_1h2k	1.00	1.00	0.0	0.0	1847	314
grayson_inoue	0.99	1.00	0.0	0.0	13605	14135
grayson_ricci	0.99	0.78	0.0	0.0	77169	59567
omegacsd_GANFIK	0.83	0.91	0.0	0.0	17924	23812
omegacsd_LINLAV	0.90	0.96	0.0	0.0	25038	97112
omegapdb_2brc	1.00	0.23	0.0	0.0	39118	58287
astex_1w2g	0.97	0.97	0.0	0.0	6200	30578
omegapdb_2byh	0.96	0.51	0.0	0.0	16000	11648
omegacsd_YUKKUK	0.66	1.00	0.0	0.0	23217	42673
grayson_hexynoate	0.96	0.43	0.0	0.1	8261	7827
grayson_gobel	0.31	1.00	0.6	0.0	8822	34530
				Total	237202	380484
				Ratio	1	1.60

Table S15A. Performance comparisons between CREST and MacroModel (PART 2: performance with CONFPASS, *ie* the incomplete set of re-optimized structures when %Conf no longer deviates below 90% based on the LR model following the priority list from the default setting of CONFPASS). The ρ_{total} and ΔG_{gl} at the corresponding r_{opt} value are reported below. ρ_{total} is the sum of the Boltzmann distribution population percentage for all the re-optimized conformers with a unique structure in the structural data set. ΔG_{gl} is the ΔG of the most stable re-optimized conformer compared to the entire set of DFT structures (*ie* all re-optimized structures from MacroModel and CREST).

	r_{opt} when %Conf no longer deviates below 90%		ρ_{total}		ΔG_{gl} (kcal mol^{-1})	
Molecule Name	MacroModel	CREST	MacroModel	CREST	MacroModel	CREST
omegapdb_1h2k	0.39	0.43	1.00	0.87	0.0	0.0
grayson_inoue	0.53	0.46	1.00	1.00	0.0	0.0
grayson_ricci	0.31	0.34	0.89	0.93	0.0	0.0
omegacsd_GANFIK	0.37	0.41	0.99	0.93	0.0	0.0
omegacsd_LINLAV	0.40	0.41	0.98	0.92	0.0	0.0
omegapdb_2brc	0.45	0.52	0.60	0.76	0.0	0.0

astex_1w2g	0.45	0.29	0.79	0.87	0.0	0.0
omegapdb_2byh	0.50	0.54	0.99	1.00	0.0	0.0
omegacsd_YUKKUK	0.61	0.34	0.95	0.89	0.0	0.0
grayson_hexynoate	0.50	0.41	0.41	0.98	0.6	0.0
grayson_gobel	0.54	0.67	0.72	1.00	0.0	0.0

Table S15B. Performance comparisons between CREST and MacroModel (PART 2: performance with CONFPASS, *ie* the incomplete set of re-optimized structures when %Conf no longer deviates below 90% based on the LR model following the priority list from the default setting of CONFPASS). The scaled CPU time below refers to the time required for re-optimizing the incomplete set of re-optimized structures when %Conf no longer deviates below 90% following the priority list from CONFPASS with the default setting.

	Total number of conformers from conformational searches		The corresponding number of conformers at the r_{opt} reported in Table S14A		Scaled CPU time	
Molecule Name	MacroModel	CREST	MacroModel	CREST	MacroModel	CREST
omegapdb_1h2k	51	7	20	3	666	150
grayson_inoue	59	57	31	26	7162	6155
grayson_ricci	223	115	70	39	25425	20211
omegacsd_GANFIK	82	88	30	36	6624	9552
omegacsd_LINLAV	40	146	16	60	10700	43999
omegapdb_2brc	155	79	69	41	16648	29117
astex_1w2g	60	212	27	61	2921	7634
omegapdb_2byh	72	41	36	22	8730	6593
omegacsd_YUKKUK	54	148	33	50	13001	14442
grayson_hexynoate	66	66	33	27	3121	3424
grayson_gobel	13	43	7	29	5533	24982
				Total	100529	166260
				Ratio	1	1.65

9. Reference

1 F. Pedregosa, V. Gael, A. Gramfort, V. Michel, B. Tririon, O. Grisel, M. Blondel, P. Prettenhofer, R. Weiss, V. Dubourg, J. Vanderplas, A. Passos and D. Cournapeau, *J. Mach. Learn. Res.*, 2011, **12**, 2825–2830.
2 RDKit Open-Source Cheminformatics. https//www.rdkit.org/ (accessed December 18, 2020).
3 Schrödinger Release 2021-2 Maestro. Schrödinger, LLC, New York, NY, 2021.
4 T. A. Halgren, *J. Comput. Chem.*, 1996, **17**, 490–519.
5 T. Lewis-Atwell, P. A. Townsend and M. N. Grayson, *J. Org. Chem.*, 2022, **87**, 5703–5712.
6 F.-L. Zhang, B. Li, K. N. Houk and Y.-F. Wang, *JACS Au*, 2022, **2**, 1032–1042.
7 Z.-L. Li, G.-C. Fang, Q.-S. Gu and X.-Y. Liu, *Chem. Soc. Rev.*, 2020, **49**, 32–48.
8 J. M. Smith, S. J. Harwood and P. S. Baran, *Acc. Chem. Res.*, 2018, **51**, 1807–1817.
9 I. Y. Kanal, J. A. Keith and G. R. Hutchison, *Int. J. Quantum Chem.*, 2018, **118**, e25512.
10 P. Pracht, F. Bohle and S. Grimme, *Phys. Chem. Chem. Phys.*, 2020, **22**, 7169–7192.
11 S. Grimme, F. Bohle, A. Hansen, P. Pracht, S. Spicher and M. Stahn, *J. Phys. Chem. A*, 2021, **125**, 4039–4054.
